# Impact of battery electric vehicle usage on air quality in three Chinese first-tier cities

**DOI:** 10.1038/s41598-023-50745-6

**Published:** 2024-01-02

**Authors:** Wenjing Lyu, Ying Hu, Jin Liu, Kaizhe Chen, Peng Liu, Junjun Deng, Shaojun Zhang

**Affiliations:** 1 Sloan School of Management, Massachusetts Institute of Technology, Cambridge, MA USA; 2 School of Humanities and Social Sciences, Beijing Institute of Technology, Beijing, China; 3 National Engineering Laboratory for Electric Vehicles, Beijing Institute of Technology, Beijing, China; 4 School of Physical Sciences, Beijing Institute of Technology, Beijing, China; 5 Institute of Air Pollution and Control, Tsinghua University, Beijing, China

**Keywords:** Climate sciences, Environmental sciences

## Abstract

China, the world leader in automobile production and sales, confronts the challenge of transportation emissions, which account for roughly 10% of its total carbon emissions. This study, utilizing real-world vehicle data from three major Chinese cities, assesses the impact of Battery Electric Vehicles (BEVs) on air quality. Our analysis reveals that BEVs, when replacing gasoline vehicles in their operational phase, significantly reduce emissions, with reductions ranging from 8.72 to 85.71 kg of CO_2_ per vehicle monthly. The average monthly reduction rate is 9.47%, though this effect is less pronounced during winter. Advanced BEVs, characterized by higher efficiency and newer technology, exhibit greater emission reduction benefits. While private BEVs generally contribute positively to environmental outcomes, taxi BEVs, due to their intensive usage patterns, show less environmental advantage and may sometimes worsen air quality. Looking ahead, we project substantial emission reductions from the replacement of gasoline vehicles with electric alternatives over the next decade. Policymakers are urged to adopt proactive measures, focusing on promoting medium to large electric vehicles and fostering the use of private and ride-hailing electric vehicles.

## Introduction

Industrialized countries, particularly China, are grappling with the challenge of mitigating air pollution amidst rapid urbanization. As per the International Energy Agency, China’s CO_2_ emissions in 2021 accounted for a staggering 33% of the global total, amounting to 11.9 billion tonnes. The transportation sector, a major contributor to China’s carbon footprint, is responsible for about 10% of its total emissions^1,2^. This environmental issue is particularly acute in China’s super-tier-1 cities, where air pollution significantly hinders socioeconomic growth and public health^3,4^.

In response to these challenges, the Chinese government has emphasized the development and adoption of New Energy Vehicles (NEVs), particularly Battery Electric Vehicles (BEVs), as a clean alternative to conventional automobiles^5,6^. By June 2022, out of 312 million civilian vehicles, only 8.104 million were BEVs, representing roughly 2.6% of the total^7,8^. This shift towards electric mobility is part of China’s broader strategy to achieve carbon neutrality, as outlined in the State Council’s Action Plan for Achieving Carbon Peaks by 2030, which targets a 40% share for new energy and clean energy-powered transport vehicles by 2030^9–11^. Globally, policy support has been instrumental in increasing the total number of electric vehicles to about 16.5 million, a threefold increase from 2018^12^. However, the realization of these targets hinges on the usage patterns and frequency of BEV utilization^13^.

Despite the rising global adoption of electric vehicles, the academic community remains divided over the environmental impact of New Energy Vehicles (NEVs). Proponents highlight NEVs’ potential in reducing carbon intensity and enhancing air quality^14^. In contrast, critics caution that BEVs might shift emissions from transportation to electricity production, particularly in regions dependent on non-renewable energy sources^15,16^. Further, there are concerns that subsidies for NEVs could divert resources from other vital environmental initiatives, potentially impeding broader urban environmental progress^17^.

This debate largely centers around different phases of NEV utilization. Advocates often focus on the usage phase and life cycle assessment (LCA) ^18,19^, while detractors draw attention to the energy production phase and its possible diversion of resources from other environmental policies^20^. The environmental footprint of BEVs, including their electricity consumption and resultant greenhouse gas emissions, varies depending on operational conditions and the battery charging/discharging process^21^. Many studies have explored BEV emission reductions at various stages, from charging to driving^22–24^. However, the limited scope of these studies often hampers a comprehensive understanding of BEVs’ impact on air quality.

In our study, we utilize large-scale real-world data to assess the impact of vehicle electrification on air quality, focusing particularly on China’s super-tier-1 cities, which predominantly rely on coal-based power generation. We analyze the relationship between BEV usage patterns—namely mileage and driving frequency—and air quality. This analysis draws upon data from Beijing, Shanghai, and Shenzhen, spanning from January 2019 to October 2020.

Our methodology involves comparing the carbon emissions of BEVs with those of fuel vehicles over comparable distances, while controlling for factors such as trip length, vehicle age, road conditions, and weather conditions. In line with existing literature^25–28^, we hypothesize that BEVs are zero-emission during the driving phase, with their primary source of pollutants being the electrical energy consumed during operation. This hypothesis forms the basis for our exploration into how a shift to BEVs, in place of gasoline vehicles, could potentially improve air quality. We assume that each electric kilometer driven offsets a corresponding distance driven by a gasoline vehicle, allowing for a direct comparison of carbon emissions across the same mileage, irrespective of external and internal factors.

Our findings indicate that replacing fuel vehicles with BEVs yields substantial emission reduction benefits and impacts various air pollutants. The study reveals significant differences in the effects of different BEV models and categories on air quality. Higher-end BEV models and private passenger BEVs are shown to significantly improve air quality. Conversely, applications such as taxi BEV usage demonstrate less favorable environmental outcomes, suggesting a nuanced impact of BEV adoption on urban air quality.

Through this research, we contribute to the understanding of how BEV usage influences air quality, providing insights into the differential impacts of various BEV models and categories. This study not only highlights the emission reduction potential of BEVs but also underscores the importance of considering vehicle usage patterns in evaluating their environmental benefits.

Our analysis aims to provide a nuanced understanding of the interplay between BEV usage and air quality, offering valuable insights for shaping sustainable urban transportation policies in China.

### Emission reduction benefits of BEVs replacement for fuel vehicles

**Figure 1:**
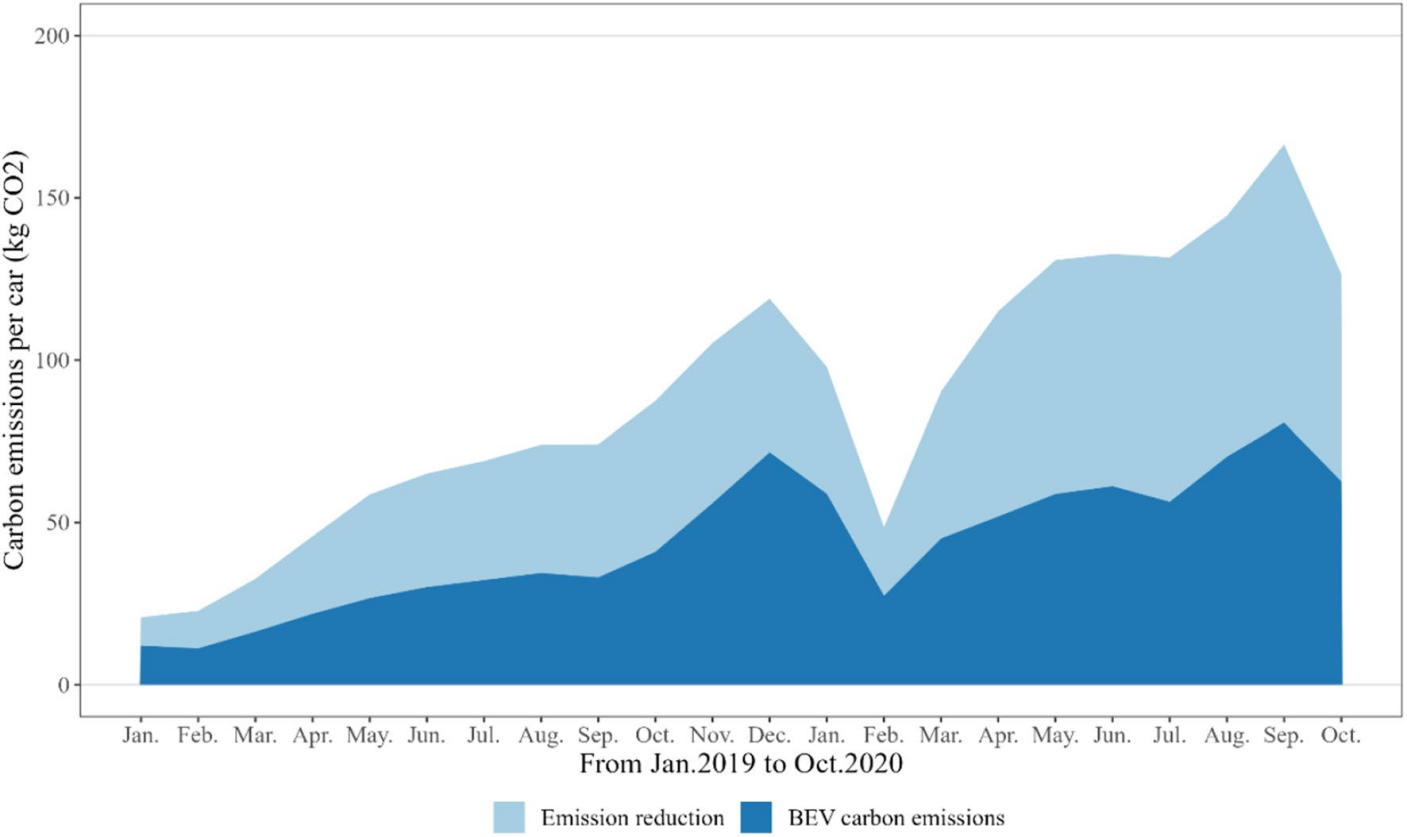
Comparative carbon emissions of gasoline vehicles and BEVs at equivalent mileages. Note: This figure illustrates the carbon emissions during the driving phase for both electric vehicles (BEVs) and gasoline vehicles over the same driving mileage. The monthly emission reduction per vehicle are calculated as the difference in carbon emissions between the gasoline vehicles and BEVs for equivalent mileages. The detailed formula used for this calculation is outlined in the “Models” section, and the specific data utilized can be found in Supplementary Data 2-1.

Utilizing real-world driving data, our study demonstrates a notable carbon reduction effect when Battery Electric Vehicles (BEVs) replace fuel vehicles over equivalent mileages (refer to Fig. 1). We observed a consistent upward trend in emission reductions over the study period. In January 2019, the emission reduction per BEV was approximately 8.72 kg of CO_2_, which escalated to around 63.83 kg of CO_2_ by October 2020, averaging a monthly increase of 9.47% (details provided in Supplementary Data 1).

Initially, the new energy vehicle market in China, including BEVs, was largely dependent on government support. However, diverse support policies have subsequently catalyzed substantial growth in this sector, positioning China as the leading market globally for new energy vehicles^29^. This growth has been further bolstered by improvements in charging infrastructure and the introduction of incentive schemes, leading to increased acceptance among Chinese residents, particularly the younger demographic^3,30^. As a result, there has been a notable shift towards BEVs, replacing conventional gasoline vehicles for similar mileage requirements, thereby contributing significantly to emission reductions in the transportation sector.

In terms of temporal patterns, BEVs exhibit optimal emission reduction performance during the months of May to July and September to November. This pattern correlates with the finding that BEVs achieve their lowest energy consumption at approximately 20 °C, and the impact of temperature on energy consumption is markedly less pronounced at higher driving speeds (around 130 km/h)^31^.

Interestingly, the winter of 2019 did not show a significant variation in CO_2_ emission reductions across the studied regions. However, from January 2020, a marked decline in CO_2_ emissions was observed, reaching a nadir two months later. This trend can likely be attributed to the national response to the initial COVID-19 outbreak, where there was a substantial reduction in intra-city vehicle travel. For instance, in Chongqing, the average daily vehicle mileage decreased by 9% post-outbreak, resulting in a 24% reduction in the carbon emissions of Plug-in Hybrid Electric Vehicles (PHEVs)^32^.

The resumption of work and production, guided by the Central Leading Group for Responding to COVID-19’s ‘Guidance on Actively and Orderly Promoting the Resumption of Work and Production while Effectively Preventing and Controlling COVID-19’, saw a return to pre-pandemic emission reduction trends from April 2020 onwards^33^.

### Differences in emission reductions between BEV models

**Figure 2:**
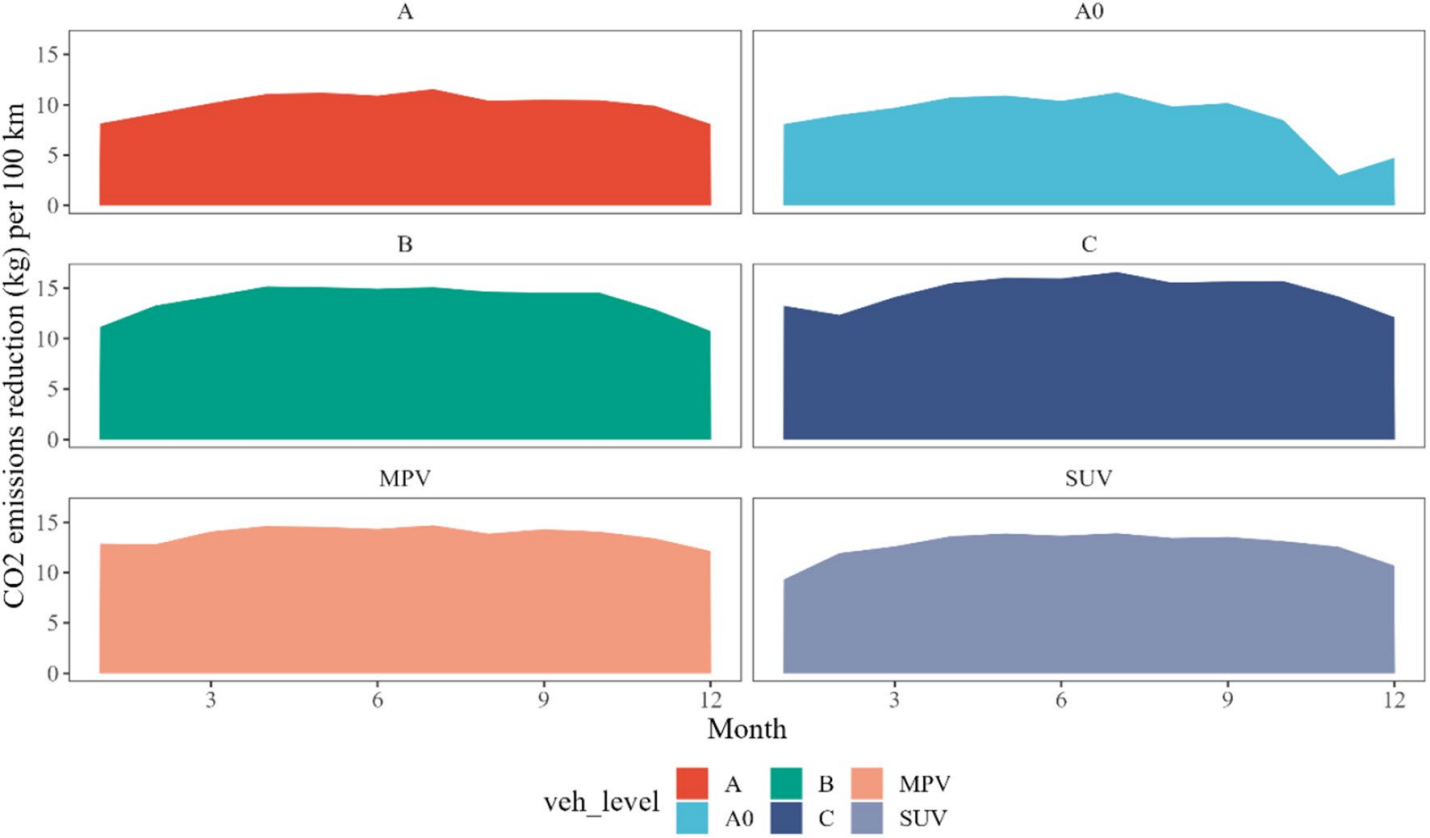
Monthly CO_2_ emission reductions per 100 km for Six BEV Types. Note: This figure displays the CO_2_ emission reductions per 100 km on a monthly basis for six different types of Battery Electric Vehicles (BEVs). The BEV types include A00-type, A0-type, A-type, B-type, C-type, SUV-type, and MPV-type BEVs. The data from January to October represent the total emission reductions for each BEV model across both 2019 and 2020. For November and December, the data shown reflect only the emission reductions for each model in 2019. Detailed source data can be found in Supplementary Data 2-2.

This study delves into the emission reduction differences among various BEV models when they replace equivalent fuel vehicles (see Fig. 2). We found a consistent trend: the larger or heavier the vehicle, the more substantial the carbon emission reduction achieved when it is replaced with a BEV of the same category. This observation can be attributed to the higher energy release and larger displacement of larger vehicles, leading to notable emission reductions when substituted with equivalent BEVs^34^.

Among the evaluated models, C-type BEVs demonstrated the most significant emission reduction benefits. On average, these vehicles yielded an annual reduction of 14.75 kg CO_2_ per 100 km. B-type vehicles followed closely, with an average annual reduction of 13.86 kg CO_2_ per 100 km. In contrast, A0-type BEVs showed the least benefit, with an average annual reduction of only 8.86 kg CO_2_ per 100 km.

Extreme weather conditions were found to increase the carbon emissions across all models, likely due to the use of air conditioning and the diminished performance of batteries in such conditions^35^. Cold temperatures, in particular, have a more pronounced impact on BEVs. The decrease in battery activity and storage capacity in colder climates, coupled with increased rolling resistance and aerodynamic drag, results in heightened energy consumption and power loss. Studies have also noted a significant drop in average EV mileage in cold climates^36^.

Furthermore, our analysis revealed that the sensitivity to temperature variations differs across BEV models. Micro-BEVs, especially those utilizing LiFePO4-based Li-ion batteries, are more susceptible to extreme climatic conditions, especially in winter. These batteries, while resistant to high temperatures, demonstrate poor performance in low temperatures, resulting in a considerable disparity in energy consumption between summer and winter^35^. This leads to higher sensitivity, with A0-type BEVs showing a 66.1% lower emission reduction per 100 km in winter months compared to the average.

### The impact of travelling in BEVs on air quality

**Figure 3:**
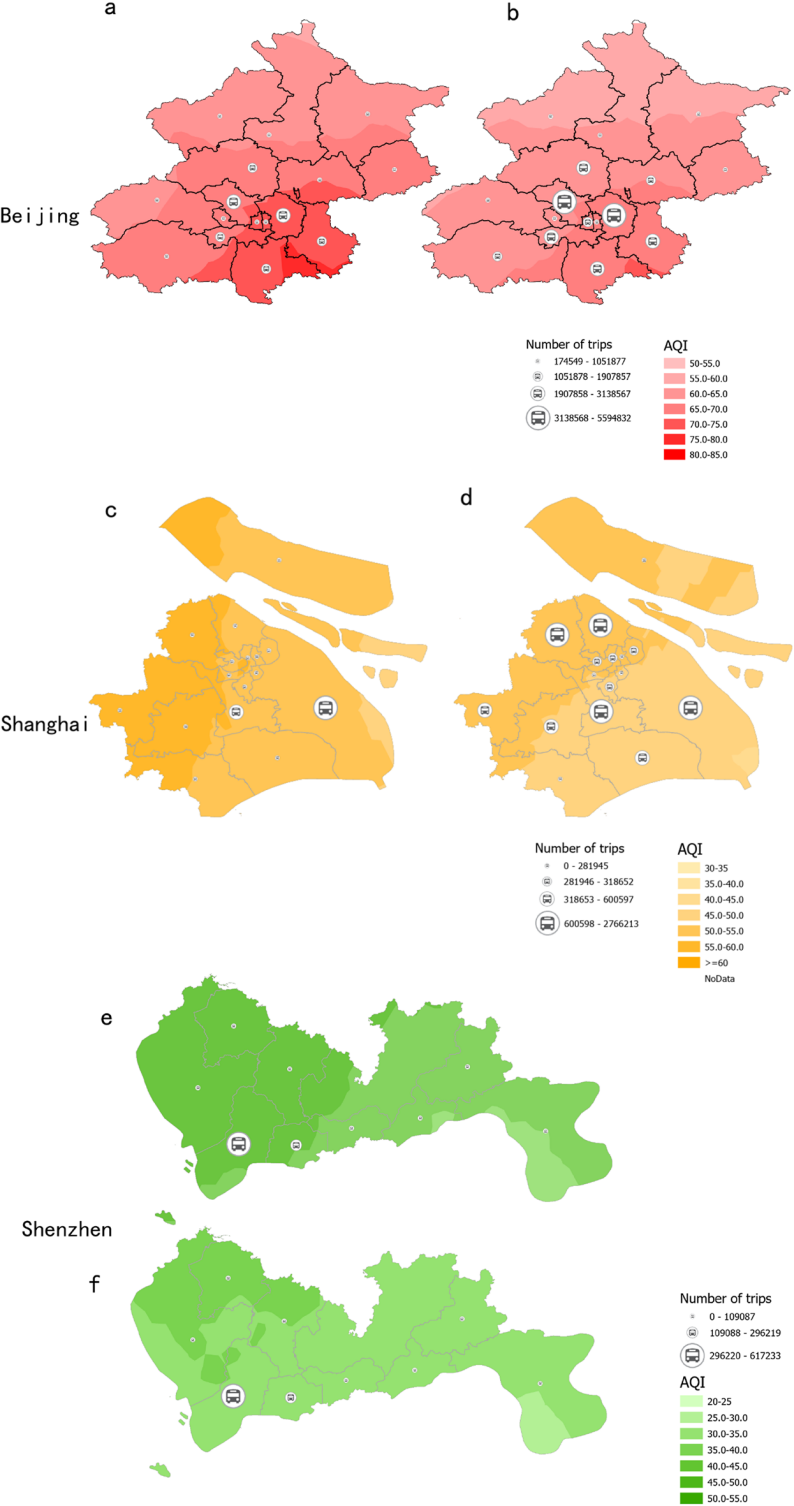
Correlation between air quality and vehicle travel frequency in three first-tier Chinese cities. Note: This figure presents an analysis of vehicle travel data and air quality across various districts in Beijing, Shanghai, and Shenzhen, based on vehicle latitude and longitude information. For Beijing, the data encompasses Changping, Chaoyang, Daxing, Dongcheng, Fangshan, Fengtai, Haidian, Huairou, Mentougou, Miyun, Pinggu, Shijingshan, Shunyi, Xicheng, and Yanqing Districts. In Shanghai, the analysis includes Baoshan, Fengxian, Hongkou, Huangpu, Jiading, Jinshan, Jing’an, Minhang, Pudong New, Putuo, Qingpu, Songjiang, Xuhui, Yangpu, and Changning Districts. For Shenzhen, the focus is on Futian and Nanshan Districts. Air quality levels were evaluated using various indicators from meteorological stations in each district, with the Air Quality Index (AQI) serving as a reference. Higher AQI values indicate more severe air pollution. The data for Shenzhen includes only Futian and Nanshan Districts; thus, the figure specifically illustrates changes in BEV travel frequency in these districts. Detailed data can be found in Supplementary Data 2-3.

Our analysis focuses on how changes in BEV travel frequency affect carbon emissions and air quality in three first-tier Chinese cities (refer to Fig. 3). We observed noticeable regional variations in air quality across Beijing, Shanghai, and Shenzhen, influenced significantly by geographical conditions.

In Beijing, characterized by its ‘back-mountain and facing-sea’ terrain, the northern regions exhibited superior air quality compared to the southern areas. Conversely, Shanghai, with its advantageous positioning along the Pacific Ocean and bordered by major water bodies, demonstrated better overall air quality than Beijing. Notably, the eastern regions of Shanghai showed improved air conditions compared to the west. Shenzhen, located near the Pearl River Estuary and Daya Bay, exhibited the best air quality among the three cities, with notable improvements towards the southeast. This variation in air quality can be partly attributed to the differing power energy structures of these cities; Beijing and Shanghai primarily rely on thermal power generation, while Shenzhen benefits from a significant proportion of nuclear power generation^37^.

Time-based analysis revealed that the frequency of BEV travel in 2020 was substantially higher than in 2019, correlating with an overall improvement in air quality. The Air Quality Index (AQI) decreased in each region during this period. In Beijing, for instance, central districts such as Haidian and Chaoyang saw significant increases in BEV travel frequencies, with corresponding decreases in AQI. The southeastern districts of Daxing and Tongzhou experienced more than a twofold increase in BEV travel frequency, leading to marked improvements in air quality.

Shanghai mirrored this trend; the Pudong New District, for example, reported the most substantial increase in BEV trips and a notable AQI decrease. Other districts, including Minhang, Baoshan, and Jiading, also saw significant upticks in BEV travel and corresponding improvements in air quality. In Shenzhen, districts like Futian and Nanshan demonstrated a decrease and an increase in BEV trips, respectively, both resulting in significant AQI reductions.

These findings suggest a positive correlation between increased BEV usage and improved air quality, indicating that BEVs, by replacing fuel vehicle trips, can effectively contribute to air quality enhancement in urban areas.

Utilizing Eq. (2) detailed in the “Methods” section, we analyzed the impact of BEV travel frequency on air quality in Beijing, Shanghai, and Shenzhen. The primary findings (summarized in Table 1) indicate that several factors influence this impact, including GDP, urban population density, policy, average temperature, humidity, precipitation, sunshine hours, and wind speed.

**Table 1:** Baseline estimates.

	(1)	(2)	(3)	(4)	(5)	(6)	(7)	(8)
	lnAQI	lnAQI	lnPM_2.5_	lnPM_10_	lnCO	lnSO_2_	lnNO_2_	lnO_3_
BEVtrips	− 0.0007***	− 0.0011***	− 0.0005***	− 0.0002	− 0.0007***	− 0.0014***	0.0025***	− 0.0063***
	(0.0002)	(0.0001)	(0.0001)	(0.0001)	(0.0001)	(0.0001)	(0.0001)	(0.0002)
lngdp		3.7261***	2.4949***	4.8514***	0.1419***	4.4983***	0.6094***	8.5795***
		(0.0199)	(0.0268)	(0.0187)	(0.0126)	(0.0137)	(0.0096)	(0.0194)
Industry		7.6482***	4.8892***	12.8716***	-2.4121***	8.8990***	− 5.1767***	19.1043***
		(0.0571)	(0.0713)	(0.0629)	(0.0283)	(0.0366)	(0.0267)	(0.0420)
lnpopula		− 0.0817***	− 0.2044***	0.4304***	0.2349***	0.5667***	− 0.0268***	0.1800***
		(0.0052)	(0.0066)	(0.0059)	(0.0026)	(0.0035)	(0.0024)	(0.0057)
Govern		0.2019***	0.1991***	0.2627***	− 0.0136***	0.1677***	0.0161***	0.2973***
		(0.0006)	(0.0009)	(0.0007)	(0.0003)	(0.0003)	(0.0005)	(0.0007)
lntemp		0.0276***	0.0177***	0.0517***	− 0.0912***	− 0.1274***	− 0.0327***	0.1622***
		(0.0003)	(0.0004)	(0.0003)	(0.0002)	(0.0002)	(0.0002)	(0.0005)
lnhumi		− 1.0840***	− 1.0594***	− 1.4243***	0.0418***	− 1.0669***	0.1149***	− 1.6298***
		(0.0023)	(0.0029)	(0.0023)	(0.0010)	(0.0012)	(0.0010)	(0.0026)
lnprecip		− 0.0336***	− 0.0680***	− 0.0302***	− 0.0037***	− 0.0165***	− 0.0391***	− 0.0360***
		(0.0002)	(0.0003)	(0.0002)	(0.0002)	(0.0003)	(0.0002)	(0.0004)
lnsun		− 0.3120***	− 0.4206***	− 0.2201***	− 0.0500***	− 0.0477***	− 0.0708***	− 0.0964***
		(0.0008)	(0.0008)	(0.0010)	(0.0007)	(0.0015)	(0.0006)	(0.0010)
lnwind		− 0.8004***	− 1.0176***	− 0.8406***	− 0.5517***	− 0.3023***	− 0.3665***	− 1.4846***
		(0.0018)	(0.0023)	(0.0016)	(0.0013)	(0.0018)	(0.0008)	(0.0029)
cons	4.3315***	− 23.4060***	− 10.6200***	− 37.5529***	− 1.8889***	− 39.4803***	0.0840	− 70.2107***
	(0.0012)	(0.1980)	(0.2617)	(0.1941)	(0.1106)	(0.1203)	(0.0948)	(0.1819)
Month	Yes	Yes	Yes	Yes	Yes	Yes	Yes	Yes
City	Yes	Yes	Yes	Yes	Yes	Yes	Yes	Yes
N	1,826,095	1,826,095	1,826,095	1,826,095	1,826,095	1,826,095	1,826,095	1,826,095
R^2^	0.5668	0.7880	0.8201	0.8548	0.9069	0.8904	0.9012	0.8466

Note: The independent variable, BEVtrips, represents the natural logarithm of the monthly travel frequency of battery electric vehicles (BEVs). The unit-of-analysis is at the BEV-month level. Columns (1) and (2) detail the relationship between BEV monthly travel frequency and the Air Quality Index (AQI). Columns (3) through (8) explore the relationship between BEV monthly travel frequency with individual air pollutants: PM_2.5_, PM_10_, CO, SO_2_, NO_2_ and O_3_, respectively. Both month and city fixed effects are controlled in all regressions. The control variables are not included for the regression in Column (1) and are included in the regressions in Columns (2)–(8). Robust standard errors are shown in parentheses. *p < 0.1; **p < 0.05; ***p < 0.01.

After adjusting for these variables, our analysis revealed that a 10% increase in monthly BEV travel frequency correlates with an average AQI decrease of 1.1%. Concurrently, the concentrations of key air pollutants—PM_2.5_, PM_10_, CO, SO_2_ and O_3_ —showed average decreases of 0.5%, 0.2%, 0.7%, 1.4%, and 6.3% respectively. However, this trend is accompanied by a 2.5% average increase in NO_2_ concentrations.

Considering that AQI is a composite indicator reflecting the levels of SO_2_, NO_2_, PM_10_, PM_2.5_, O_3_ and CO, an increase in BEV travel frequency overall promotes environmental benefits. This aligns with the findings of Liang et al., who observed a significant reduction in PM_2.5_ and O_3_ concentrations with increased BEV usage in China^4^.

Therefore, augmenting BEV usage not only reduces the reliance on gasoline vehicles but also effectively mitigates air pollution. However, our results differ slightly from Liang et al.^4^ and Chen et al.^38^ who predicted a decrease in NO_2_ concentrations with increased BEV usage. This discrepancy may stem from our study’s focus on the actual frequency of BEV usage and its direct impact on air pollutants, as opposed to the predicted scenarios for 2030 used in their studies.

The increase in NO_2_ concentrations we observed could be attributed to the electricity consumption of BEVs. Beijing and Shanghai predominantly rely on thermal power generation, which emits significant amounts of nitrogen oxides and particulate matter. Hence, while emissions during BEV driving stages are reduced, the NO_2_ produced by coal power consumption for BEV charging might outweigh these reductions, leading to an overall increase in atmospheric NO_2_ concentrations.

### Air quality impacts of BEVs for different applications

Utilizing the method outlined in Eq. (2), we analyzed the air quality impacts of private BEVs, taxi BEVs, and online ride-hailing BEVs, each detailed in Tables 2, 3, and 4, respectively.

**Table 2:** Heterogeneity analysis: private BEVs.

	(1)	(2)	(3)	(4)	(5)	(6)	(7)
	lnAQI	lnPM_2.5_	lnPM_10_	lnCO	lnSO_2_	lnNO_2_	lnO_3_
BEVtrips	− 0.0007***	− 0.0005***	− 0.0005***	− 0.0006***	− 0.0005***	0.0011***	− 0.0024***
	(0.0001)	(0.0001)	(0.0001)	(0.0000)	(0.0001)	(0.0001)	(0.0001)
lngdp	2.2396***	0.2369***	4.0528***	− 0.3066***	4.2063***	0.1497***	9.1053***
	(0.0225)	(0.0289)	(0.0299)	(0.0142)	(0.0181)	(0.0175)	(0.0308)
Industry	1.4338***	− 2.6928***	6.4481***	− 4.6206***	7.8732***	− 6.5059***	17.0918***
	(0.0714)	(0.0745)	(0.0949)	(0.0330)	(0.0554)	(0.0470)	(0.0928)
lnpopula	− 0.8295***	− 1.1389***	− 0.4523***	0.0691***	0.5647***	− 0.1673***	− 0.4481***
	(0.0172)	(0.0218)	(0.0176)	(0.0090)	(0.0120)	(0.0087)	(0.0244)
Govern	0.1275***	0.0710***	0.2129***	− 0.0377***	0.1805***	− 0.0237***	0.3887***
	(0.0010)	(0.0015)	(0.0010)	(0.0005)	(0.0005)	(0.0009)	(0.0014)
lntemp	− 0.0004	0.0068***	− 0.0244***	− 0.1096***	− 0.1258***	− 0.0294***	0.0413***
	(0.0006)	(0.0006)	(0.0009)	(0.0004)	(0.0004)	(0.0004)	(0.0015)
lnhumi	− 0.8202***	− 0.5984***	− 1.2309***	0.0114***	− 1.0238***	0.0418***	− 1.5934***
	(0.0054)	(0.0069)	(0.0065)	(0.0020)	(0.0022)	(0.0021)	(0.0053)
lnprecip	0.0297***	− 0.0121***	0.0319***	0.0340***	0.0228***	− 0.0493***	0.0690***
	(0.0007)	(0.0008)	(0.0007)	(0.0005)	(0.0004)	(0.0003)	(0.0014)
lnsun	− 0.1772***	− 0.3094***	− 0.0380***	0.0257***	0.1617***	− 0.1250***	− 0.0295***
	(0.0017)	(0.0021)	(0.0022)	(0.0010)	(0.0014)	(0.0009)	(0.0021)
lnwind	− 0.6822***	− 0.7914***	− 0.7085***	− 0.7247***	− 0.5723***	− 0.4304***	− 1.3031***
	(0.0030)	(0.0039)	(0.0039)	(0.0019)	(0.0023)	(0.0011)	(0.0037)
cons	− 5.3942***	15.1315***	− 24.4810***	3.5309***	− 37.5438***	5.8214***	− 70.5363***
	(0.2744)	(0.3442)	(0.3419)	(0.1451)	(0.1869)	(0.1798)	(0.3375)
Month	Yes	Yes	Yes	Yes	Yes	Yes	Yes
City	Yes	Yes	Yes	Yes	Yes	Yes	Yes
N	1,369,978	1,369,978	1,369,978	1,369,978	1,369,978	1,369,978	1,369,978
R^2^	0.8609	0.8868	0.9119	0.9568	0.9592	0.9529	0.9483

Note: The independent variable, BEVtrips, represents the natural logarithm of the monthly travel frequency of battery electric vehicles (BEVs). The unit-of-analysis is at the BEV-month level. Column (1) details the relationship between private BEV monthly travel frequency and the Air Quality Index (AQI). Columns (2) through (7) explore the relationship between private BEV monthly travel frequency with individual air pollutants: PM_2.5_, PM_10_, CO, SO_2_, NO_2_ and O_3_, respectively. All regressions account for both month and city fixed effects, and incorporate control variables to ensure robustness and accuracy in capturing the true impact of private BEV travel frequency on air quality. Robust standard errors are shown in parentheses. *p < 0.1; **p < 0.05; ***p < 0.01.

**Table 3:** Heterogeneity analysis: Taxi BEVs.

	(1)	(2)	(3)	(4)	(5)	(6)	(7)
	lnAQI	lnPM_2.5_	lnPM_10_	lnCO	lnSO_2_	lnNO_2_	lnO_3_
BEVtrips	0.0150***	0.0228***	0.0125***	0.0008***	0.0018***	− 0.0055***	0.0131***
	(0.0004)	(0.0006)	(0.0004)	(0.0001)	(0.0001)	(0.0002)	(0.0004)
lngdp	− 2.6019***	− 6.6081***	3.1029***	− 3.1101***	− 1.3691***	6.5408***	− 7.2754***
	(0.1360)	(0.1996)	(0.1321)	(0.0551)	(0.0497)	(0.0277)	(0.1391)
Industry	− 5.3412***	− 9.8655***	5.4159***	− 4.1792***	− 1.6209***	20.8273***	− 21.8003***
	(0.4803)	(0.7101)	(0.4368)	(0.2124)	(0.1471)	(0.2066)	(0.4907)
lnpopula	− 0.3223***	− 0.1100***	− 0.4848***	0.3256***	0.6120***	0.1280***	− 0.5266***
	(0.0135)	(0.0203)	(0.0107)	(0.0063)	(0.0084)	(0.0053)	(0.0151)
Govern	0.3492***	0.3886***	− 0.0020	0.0575***	0.3134***	− 0.0222***	0.3243***
	(0.0053)	(0.0083)	(0.0052)	(0.0027)	(0.0046)	(0.0018)	(0.0063)
lntemp	− 0.1765***	− 0.2899***	0.3122***	− 0.2365***	− 0.3426***	− 0.1296***	0.1806***
	(0.0058)	(0.0090)	(0.0058)	(0.0026)	(0.0040)	(0.0021)	(0.0058)
lnhumi	− 0.6927***	− 0.5579***	− 1.7540***	0.4433***	− 0.4003***	0.1591***	− 1.7778***
	(0.0176)	(0.0255)	(0.0139)	(0.0065)	(0.0109)	(0.0058)	(0.0170)
lnprecip	− 0.1594***	− 0.2129***	− 0.1294***	− 0.0742***	− 0.0550***	− 0.0999***	− 0.0726***
	(0.0012)	(0.0018)	(0.0010)	(0.0006)	(0.0007)	(0.0004)	(0.0014)
lnsun	− 0.4536***	− 0.5445***	− 0.5846***	− 0.0400***	− 0.0429***	− 0.1473***	− 0.2963***
	(0.0034)	(0.0051)	(0.0031)	(0.0016)	(0.0035)	(0.0014)	(0.0047)
lnwind	− 0.4782***	− 0.9116***	− 0.1040***	− 0.3735***	0.0470***	− 0.4298***	− 0.8958***
	(0.0071)	(0.0105)	(0.0060)	(0.0026)	(0.0036)	(0.0026)	(0.0062)
cons	38.0254***	73.0959***	− 10.8924***	25.1756***	12.1242***	− 61.1721***	89.7092***
	(1.3607)	(2.0013)	(1.3013)	(0.5405)	(0.4501)	(0.3131)	(1.3780)
Month	Yes	Yes	Yes	Yes	Yes	Yes	Yes
City	Yes	Yes	Yes	Yes	Yes	Yes	Yes
N	141,762	141,762	141,762	141,762	141,762	141,762	141,762
R^2^	0.9565	0.9508	0.9763	0.9399	0.9730	0.9872	0.9536

Note: The independent variable, BEVtrips, represents the natural logarithm of the monthly travel frequency of battery electric vehicles (BEVs). The unit-of-analysis is at the BEV-month level. Column (1) details the relationship between taxi BEV monthly travel frequency and the Air Quality Index (AQI). Columns (2) through (7) explore the relationship between taxi BEV monthly travel frequency with individual air pollutants: PM_2.5_, PM_10_, CO, SO_2_, NO_2_ and O_3_, respectively. All regressions account for both month and city fixed effects, and incorporate control variables to ensure robustness and accuracy in capturing the true impact of taxi BEV travel frequency on air quality. Robust standard errors are shown in parentheses. *p < 0.1; **p < 0.05; ***p < 0.01.

**Table 4:** Heterogeneity analysis: Ride-hailing BEVs.

	(1)	(2)	(3)	(4)	(5)	(6)	(7)
	lnAQI	lnPM_2.5_	lnPM_10_	lnCO	lnSO2	lnNO2	lnO3
BEVtrips	− 0.0004	0.0003	0.0017***	0.0008***	0.0005**	0.0028***	− 0.0064***
	(0.0003)	(0.0004)	(0.0003)	(0.0002)	(0.0002)	(0.0002)	(0.0004)
lngdp	3.6317***	2.3301***	4.7422***	− 0.3675***	3.8677***	1.4354***	6.4401***
	(0.0382)	(0.0521)	(0.0335)	(0.0190)	(0.0228)	(0.0187)	(0.0597)
Industry	9.1720***	7.5611***	13.3100***	− 2.3952***	7.9436***	− 3.2861***	16.2161***
	(0.0908)	(0.1179)	(0.0938)	(0.0399)	(0.0577)	(0.0484)	(0.1104)
lnpopula	− 0.0749***	− 0.0476***	0.3284***	0.1646***	0.3346***	− 0.0236***	0.1474***
	(0.0067)	(0.0080)	(0.0079)	(0.0030)	(0.0034)	(0.0029)	(0.0075)
Govern	0.2161***	0.2212***	0.2788***	− 0.0062***	0.1586***	0.0335***	0.2611***
	(0.0011)	(0.0015)	(0.0014)	(0.0006)	(0.0006)	(0.0008)	(0.0014)
lntemp	0.0285***	0.0127***	0.0751***	− 0.0880***	− 0.1256***	− 0.0358***	0.1897***
	(0.0005)	(0.0006)	(0.0004)	(0.0002)	(0.0003)	(0.0003)	(0.0007)
lnhumi	− 1.0294***	− 1.0735***	− 1.3501***	0.1357***	− 0.9685***	0.0937***	− 1.4838***
	(0.0038)	(0.0042)	(0.0038)	(0.0020)	(0.0030)	(0.0019)	(0.0051)
lnprecip	− 0.0608***	− 0.0927***	− 0.0555***	− 0.0236***	− 0.0387***	− 0.0391***	− 0.0765***
	(0.0004)	(0.0005)	(0.0004)	(0.0002)	(0.0004)	(0.0003)	(0.0006)
lnsun	− 0.3474***	− 0.4372***	− 0.2783***	− 0.0839***	− 0.1597***	− 0.0778***	− 0.1144***
	(0.0010)	(0.0014)	(0.0012)	(0.0012)	(0.0025)	(0.0008)	(0.0019)
lnwind	− 0.7400***	− 1.0350***	− 0.7522***	− 0.4088***	− 0.0364***	− 0.3628***	− 1.4249***
	(0.0034)	(0.0039)	(0.0031)	(0.0017)	(0.0025)	(0.0016)	(0.0063)
cons	− 23.2430***	− 10.9216***	− 36.6064***	2.9428***	− 32.1257***	− 7.1975***	− 51.2195***
	(0.3833)	(0.5135)	(0.3533)	(0.1675)	(0.1927)	(0.1734)	(0.5713)
Month	Yes	Yes	Yes	Yes	Yes	Yes	Yes
City	Yes	Yes	Yes	Yes	Yes	Yes	Yes
N	314,355	314,355	314,355	314,355	314,355	314,355	314,355
R^2^	0.8097	0.8285	0.8706	0.8210	0.8181	0.8765	0.7389

Note: The independent variable, BEVtrips, represents the natural logarithm of the monthly travel frequency of battery electric vehicles (BEVs). The unit-of-analysis is at the BEV-month level. Column (1) details the relationship between ride-hailing BEV monthly travel frequency and the Air Quality Index (AQI). Columns (2) through (7) explore the relationship between ride-hailing BEV monthly travel frequency with individual air pollutants: PM_2.5_, PM_10_, CO, SO_2_, NO_2_ and O_3_, respectively. All regressions account for both month and city fixed effects, and incorporate control variables to ensure robustness and accuracy in capturing the true impact of ride-hailing BEV travel frequency on air quality. Robust standard errors are shown in parentheses. *p < 0.1; **p < 0.05; ***p < 0.01.

Table 2 indicates that private BEV travel substantially benefits air quality. A negative correlation was found between the amount of private BEV travel frequency and the AQI index, accompanied by a decrease in of PM_2.5_, PM_10_, CO, SO_2_ and O_3_ levels, but an increase in NO_2_ levels, aligning with the overall sample results.

Conversely, Table 3 shows that taxi BEVs do not significantly impact environmental improvement and may even contribute to a decline in air quality. This is evidenced by increased levels of PM_2.5_, PM_10_, CO, SO_2_ and O_3_, with a decrease in NO_2_. The inefficiency of taxi BEVs in air quality improvement is attributed to their high energy consumption, particularly at lower temperatures and during nighttime charging^23^. As the power grid in the examined super-first-tier cities is predominantly coal-based, the electrification of taxi BEVs leads to increased emissions of greenhouse gases and pollutants^39,40^. Additionally, the unique operating conditions of taxi BEVs, such as extensive outdoor travel, frequent braking and throttling, and high charging demands, result in increased electricity consumption and accelerated battery aging, further diminishing efficiency and exacerbating air quality issues.

In contrast, Table 4 highlights that travel in online ride-hailing BEVs significantly reduces emissions, leading to improved air quality. Although there is an increase in PM2.5, PM10, CO, SO_2_, and NO_2_, the reduction in O_3_ is noteworthy. Despite similar operating conditions to electric taxis, online ride-hailing cars are comparatively more efficient, consuming 36% less fuel and producing 44% fewer hydrocarbons^41^. Thus, while they contribute positively to air quality, they also generate additional pollutants.

## Discussion

This study evaluated the impact of Battery Electric Vehicles (BEVs) on air quality in China’s three first-tier cities, utilizing real-world vehicle data and carbon emission modeling. Our analysis reveals that BEVs, when replacing fuel vehicles for equivalent mileages, generally contribute to better emission reductions. However, this effect varies across different cities, months, and vehicle models, with a notable reduction in efficacy during winter. Larger vehicles, such as B, C, and MPV models, exhibit higher carbon efficiency over 100 km compared to smaller vehicles (e.g., A0, A) and show less sensitivity to climatic changes.

The study also found a significant decrease in total vehicle mileage (fuel vehicles + BEVs) in 2020, largely attributable to COVID-19 related restrictions^42^. Interestingly, BEVs experienced a smaller change in total travel mileage but an increase in travel frequency, possibly influenced by rising fuel prices due to the Russia-Ukraine conflict^43^, making electric vehicles a more attractive travel option^44^.

Further analysis using a two-way fixed-effect model on air quality and BEV trip frequency revealed that increased BEV usage significantly improves air quality by reducing levels of PM_2.5_, PM_10_, CO, SO_2_ and O_3_. Environmental benefits vary across BEV classes, with A0-, A-, B-, and SUV-type BEVs making a substantial impact on reducing AQI and improving air quality. In contrast, A00-, C-, and MPV-type BEVs show less pronounced benefits due to lower emissions, fewer trips, and higher energy consumption for longer ranges.

The study also differentiates the environmental impacts of BEVs based on their usage. Private passenger BEVs substantially improve air quality, while taxi BEVs have lesser environmental benefits due to their operational characteristics. Online ride-hailing BEVs, having lower energy consumption per unit than taxi BEVs, offer slightly better environmental advantages.

Based on our findings, we propose several strategic recommendations to enhance the environmental efficacy of Battery Electric Vehicles (BEVs). We advocate for the prioritization of larger BEVs, as A-class and higher models demonstrate more significant carbon emission reduction benefits over their entire lifecycle compared to smaller BEVs. Additionally, the continuous monitoring of pollutants, especially NO2, is crucial to understand and mitigate the potential environmental impacts of widespread BEV adoption. Advancements in battery technology are also imperative, with the development of new-generation solid fuel cells and low-temperature-resistant ternary lithium batteries essential for maintaining emission efficiency in cold weather. Ternary lithium batteries, functional down to -30℃, exhibit superior low-temperature discharge performance, and the use of iron phosphate lithium batteries, with less than 15% mileage degradation in winter, could significantly enhance EV performance in extreme conditions. Furthermore, promoting online ride-hailing services can offer a more sustainable alternative to traditional taxi BEVs, covering wider areas and providing cost-effective, time-efficient commuting options^41^. Implementing energy-efficient air conditioning and electric power systems is pivotal for reducing winter energy consumption and enhancing EV efficiency, addressing the increased energy demands of EVs during colder months. Finally, policymakers should encourage more eco-friendly travel methods during winter, such as buses, subways, shared bicycles, trolley buses, and carpooling, which are particularly suitable for short-distance journeys and in areas with challenging road conditions. These recommendations collectively aim to maximize the environmental benefits of BEVs, contributing to sustainable urban transportation solutions and addressing the challenges posed by climate variability and technological limitations. These recommendations aim to maximize the environmental benefits of BEVs, contributing to sustainable urban transportation solutions and addressing the challenges posed by climate variability and technological limitations.

However, this study does not account for specific conditions such as extreme winter temperatures, which may increase power consumption, indicating a need for further research. Future studies should delve deeper into the interplay between BEVs, carbon emission reduction, air quality, and the substitution patterns between BEVs and fuel vehicles.

## Methods

### Data

#### BEVs driving data and air quality data

In this paper, we collected available data from the open lab of the National Big Data Alliance of New Energy Vehicles. We cleaned the data and kept all normal driving segment data. The rules followed were as follows: (1) remove all PHEV vehicles; (2) remove samples with abnormal energy consumption where the post-driving battery percentage difference SOC ≥ 0, which is the remaining power of the battery; (3) remove samples with an average speed greater than 200 km/h in the driving segments; (4) remove samples with a driving range less than 0.1 km in the driving segments.

Each driving segment in this study database contained the number of each type of electric vehicles, the time of the start and end of the drive, the start and end mileage, the start and end SOC, the duration of the drive, the average speed, the maximum speed, the longitude and latitude of the start of the drive, the longitude and latitude of the end of the drive, the distance difference, the type and use of the vehicle, the battery material and capacity, the maximum range and the net weight of the vehicle.

In 2016, the National Monitoring and Management Centre for New Energy Vehicles was established in China. It serves as the national big data platform for EVs. The centre has the only datasets in the world that contain real-time operating data of nationwide EVs. The coverage of the platform can reach up to 80% in Beijing and Shanghai^45^. In recent years, Shenzhen, as a super-first-tier city in China, has also vigorously promoted new energy vehicles. By the end of 2020, the number of new energy vehicles in Shenzhen reached 480,000, accounting for about 14% of the city’s motor vehicle ownership^46^. Our data sample in this paper consisted of panel data for three super-tier-1 cities in China (Beijing, Shanghai and Shenzhen) from January 2019 to October 2020. In the empirical model analysis, we classified the BEVs data sample by city and month to obtain the total number of trips per vehicle per month as one observation. If a vehicle did not make a trip in a month, no observation was recorded for that month for that vehicle. In total, there were 1,826,095 observations for 196,387 vehicles in the three cities, including 1,288,697 trips for 132,644 vehicles in Beijing, 239,742 trips for 22,240 vehicles in Shanghai and 297,656 trips for 41,503 vehicles in Shenzhen. The detailed data are shown in Supplementary Data 1. In addition to BEVs data, air quality data and meteorological data for each district and county in the three cities were obtained based on publicly available data in terms of year, quarter and month.

AQI, CO_2_, SO_2_, O_3_, PM_2.5_, PM_10_ and other data indicating air quality for the three cities were obtained from the real-time national urban air quality release platform of the China General Environmental Monitoring Station at https://air.cnemc.cn:18007/; GDP per capita, share of secondary industry in GDP and urban population density are taken from the China Urban Statistical Yearbook for previous years; implementation of policies restricting the driving and purchase of conventional gasoline engines from the official websites of municipalities and public security bureaus, etc^47^. Average temperature, humidity, precipitation, sunshine hours, wind speed and other meteorological data for the three cities were taken from the National Climatic Data Centre (NCDC) at https://ngdc.noaa.gov/.

#### Factors leading to carbon emission in fuel vehicles

As the BEVs considered in this study were mainly sedans, the fuel for the corresponding car model of the gasoline vehicles was mainly petrol. Therefore, the carbon emission factor for gasoline vehicles driving was taken as the carbon emission factor for petrol. The carbon emission factors of fuel vehicles, ${\text{E}}_{{{\text{co}}_{2} }}$^23,27^ was calculated as follows:

1 $$ {\text{E}}_{{{\text{co}}_{2} }} = {\text{CQ}}_{{\text{g}}} \times {\text{CF}}_{{{\text{co}}_{2} }} $$

where, ${\text{E}}_{{{\text{co}}_{2} }}$ denotes CO_2_ emissions per litre of gasoline in kg CO_2_, ${\text{CQ}}_{{\text{g}}}$ denote default gasoline net calorific value in KJ/kg. According to the General Rules for Calculating Comprehensive Energy Consumption (GBT2589-2020) issued by the State Administration of Market Administration of China and the State Standardization Administration, the net calorific value of gasoline is 43,124 kJ/kg^48^. ${\text{CF}}_{{{\text{co}}_{2} }}$ is the default value of the effective CO_2_ emission factor for petrol in KG/TJ. We referred to the IPCC guidelines for fuel carbon emission factors to obtain a default value of 74 100 kg/TJ for the effective CO_2_ emission factor of petrol^49^. Therefore, 3.1954884 kg CO_2_ is emitted per kilogram of gasoline. According to information from PacificCar.com, there are three gasoline brands in China, 90, 93 and 97, which have an average density of 0.720 g/mL, 0.725 g/mL and 0.737 g/mL, respectively^50^. We took the average of the densities of the three types of gasoline as the density of gasoline, i.e., 0.7273333 g/mL. Therefore, each litre of gasoline emitted 2.3241852 kg CO_2_, i.e., the carbon emission factor of the gasoline vehicles was 2.3241852 kg CO_2_/L.

### Models

#### Carbon emission model for the driving phase of BEVs

We developed a model for calculating the carbon emissions of BEVs during their driving phase based on real vehicle data from three cities: Beijing, Shanghai and Shenzhen. As BEVs do not produce carbon emissions during driving, all references to BEVs carbon emissions in the text refer to the carbon emissions from electricity production corresponding to the energy consumed while driving. The model was primarily divided into two parts: carbon emissions from fuel vehicles and those from BEVs. The emission savings per kilometre of a BEVs were calculated by taking the difference between the emissions calculated in Eq. (2) with the corresponding gasoline vehicle emissions^23^, represented as:

2 $$ \Delta {\text{CE}} = {\text{F}}_{{{\text{co}}_{2} }} \times {\text{C}}_{{\text{f}}} - {\text{E}}_{{{\text{co}}_{2} }} \times {\text{C}}_{{\text{e}}} $$

where, ΔCE denotes emission savings per kilometre of a BEVs in kg CO_2_; ${\text{F}}_{{{\text{co}}_{2} }}$ denotes carbon emission factors of fuel vehicles in kg CO_2_/L; ${{\text{C}}}_{{\text{f}}}$ denotes fuel consumption of fuel-powered vehicles in L/100 km; ${{\text{E}}}_{{{\text{co}}}_{2}}$ denotes electric carbon emission factor in kg CO_2_/(kW·h) and ${\text{C}}_{{\text{e}}}$ denotes actual grid energy consumption in kW·h/100 km.

#### Air quality model for the driving phase of BEVs

Based on the analysis (Supplementary information 1, 2 and 4), the air quality model of BEVs travel was constructed as follows:

3 $$ \ln {\text{AQI}}_{{{\text{c}},{\text{t}}}} = {\beta \text{*BEVtrips}}_{{{\text{c}},{\text{t}}}} + {\gamma \text{Control}}_{{{\text{c}},{\text{t}}}} + {\upmu }_{{\text{c}}} + {\upmu }_{{\text{t}}} + {\upvarepsilon }_{{{\text{c}},{\text{t}}}} $$

The dependent variable $\ln {\text{AQI}}_{{{\text{c}},{\text{t}}}}$ represents the natural logarithm of the Air Quality Index (AQI) for city *c* in month *t*, which represents the level of air pollution in the city. Contaminants for AQI calculations and evaluation include sulfur dioxide (SO_2_), nitrogen dioxide (NO_2_), suction particulate matter (PM_10_), fine particulate matter (PM_2.5_), carbon monoxide (CO) and ozone (O_3_). To more comprehensively reflect the emission reduction effect of BEV trips replacing fuel vehicle trips, we also included CO, SO_2_, O_3_, PM_2.5_ and PM_10_ as dependent variables, and recorded their logarithmic values as lnCO, lnSO_2_, lnO_3_, lnPM_2.5_, and lnPM_10_.

The independent variable ${{\text{BEVtrips}}}_{{\text{c}},{\text{t}}}$ is the log of BEV's monthly trips. The more the fuel vehicles are driven, the more emissions they produce^28^. Assuming a constant total monthly travel demand, more BEVs trips per month meant fewer fuel vehicle trips per month and stronger air quality improvement. ${{\text{Control}}}_{{\text{c}},{\text{t}}}$ are control variables affecting air pollution, including GDP (lngpd), share of secondary industry in GDP (Industry), urban population density (lnpopula), a dummy variable Govern for whether policies restricting driving and purchasing of conventional gasoline engines are implemented, average temperature (lntemp), humidity (lnhumi), precipitation (lnprecip), sunshine hours (lnsun), and wind speed (lnwind). The classical environmental Kuznets curve (EKC) theory postulates that the relationship between air pollution and economic growth is a significant inverted U-shaped curve^51^. In general, the higher the GDP per capita, the higher the vehicle stock and associated carbon emissions^52,53^. Therefore, GDP (lngpd) was included in the model. The secondary sector contributed the most to China's energy consumption and emissions^54^; hence, the higher the proportion of the secondary sector, the more serious the air pollution. Therefore, the share of the secondary sector in the GDP (Industry) was used to control the impact of the secondary sector^55^. Urban population concentrations and activities can contribute to air pollution^56^, so we controlled for the possible effects of urban population concentrations using the urban population density (lnpopula), which was the urban population divided by the area of the urban administrative district. Vehicle emissions are a significant source of urban air pollution, and the Chinese government has implemented policies to restrict the driving and purchasing of conventional gasoline engines^57^ hich restricted vehicle trips. We added a dummy variable (Govern) for whether the city implements these two restrictive policies. Air pollution is most likely related to natural climatic conditions, such as local dispersion and the deposition of pollutants. Hence, we used annual average temperature (lntemp), humidity (lnhumi), precipitation (lnprecip), sunshine duration (lnsun) and wind speed (lnwind) to control the effects of different natural climatic factors on air pollution. ${\upmu }_{{\text{c}}}$ are city fixed effects. ${\upmu }_{{\text{t}}}$ are city fixed effects. Robust standard errors ${\upvarepsilon }_{{{\text{c}},{\text{t}}}}$ are clustered at the city-month level.

### Fuel consumption by fuel vehicles

The fuel mileage of gasoline vehicles is based on the weighted average of the sales volume and fuel mileage of gasoline vehicles sold each year, and the annual sales volume and fuel consumption information are obtained from the owner's home website (www.16888.com). The price for getting on the website ranges from 30,000 yuan to 650,000 yuan, and fuel consumption spans from 6 L/100 km to 18 L/100 km, which is a good representation. The formula for calculating the 100 km fuel consumption for fuel vehicles is as follows:

4 $$ {\text{C}}_{{\text{f}}} = \frac{{\mathop \sum \nolimits_{{{\text{i}} = 1}}^{{\text{n}}} \left( {{\text{Q}}_{{\text{i}}} \times {\text{C}}_{{{\text{f}}_{{\text{i}}} }} } \right)}}{{\mathop \sum \nolimits_{{{\text{i}} = 1}}^{{\text{n}}} {\text{Q}}_{{\text{i}}} }} $$

where ${\text{C}}_{{\text{f}}}$ is the fuel consumption of a gasoline vehicle in L/100 km per 100 km, ${\text{Q}}_{{\text{i}}}$ is the annual sales of fuel vehicles in units, *f* denotes the model, *i* is the annual sales ranking and n takes the value according to different models. For convenience, this paper compares gasoline vehicle models and BEVs models as follows: according to the length, wheelbase and displacement of vehicles, BEVs models can be divided into A00-, A0-, A-, B-, C-, SUV- and MPV-type vehicles. Amongst them, gasoline vehicle models of micro car, small car, medium car and medium and large car correspond to A00-, A0-, A-, B- and C-type models of BEV, respectively; gasoline vehicle SUV models correspond to BEV's SUV models; gasoline vehicle MPV models correspond to BEV MPV models and gasoline vehicle sedan models correspond to BEV's A00-, A0-, A-, B- and C-type models.

### BEV energy consumption

The 100 km energy consumption of BEVs was calculated based on the driving segment and charging segment data of the vehicle, and the actual grid energy consumption of each vehicle was obtained based on the changes in the driving range and battery power of BEVs, as shown in Eq. (5) ^58^:

5 $$ {\text{C}}_{{\text{e}}} = 100 \times \frac{{\mathop \sum \nolimits_{{{\text{i}} = 1}}^{{\text{n}}} \left( {{\text{soc}}_{{{\text{i}}1}} - {\text{soc}}_{{{\text{i}}0}} } \right) \times {\text{P}}_{{\text{i}}} }}{{\mathop \sum \nolimits_{{{\text{i}} = 1}}^{{\text{m}}} {\text{S}}_{{\text{j}}} }} $$

where, ${\text{C}}_{{\text{e}}}$ is the power consumption in kW·h/100 km over a period of time. We calculated the total power consumption in travelling 100 km for each vehicle for one month; ${\text{soc}}_{{{\text{i}}1}}$ was the remaining power at the end of the ith charging segment; ${\text{soc}}_{{{\text{i}}0}}$ was the remaining power at the beginning of the ith charging segment; ${\text{P}}_{{\text{i}}}$ is the battery capacity in kW·h corresponding to the *i*th charging segment and ${\text{S}}_{{\text{j}}}$ is the mileage in km of the *j*th driving segment.

### Electric carbon emission factors

We calculated the local carbon emission factor for electricity based on the regional energy mix, i.e. the carbon dioxide emissions generated per 1 kW·h of electricity output from the grid in each region. In this paper, we refer to the BEV energy conversion method and the reference value of the National Standard of the People's Republic of China (GB/T 37340-2019) to calculate the carbon emission factor of electricity. The specific calculation method is shown in Eq. (6):

6 $$ {\text{E}}_{{{\text{co}}_{2} }} = \frac{{{\text{T}}_{{\text{E}}} \times {\text{T}}_{{\text{C}}} \times {{\varphi }}}}{{{\text{t}}_{{\text{M}}} \times {\text{i}}_{{{\text{c}}h}} \times \left( {1 - {\text{i}}_{{{\text{tr}}}} } \right)}} $$

where ${\text{E}}_{{{\text{co}}_{2} }} { }$ was the electricity CO_2_ emission factor in kg CO_2_/(kW h). ${\text{T}}_{{\text{E}}}$ was the standard coal consumption for thermal power supply in kg/(kW h), which was 0.3064 kg/(kW h) and 0.3055 kg/(kW h) in 2019 and 2020, respectively^59^. ${\text{T}}_{{\text{C}}}$ was the CO_2_ emission factor for fuel coal, using the parameter value of 3.09; ${{\varphi }}$ was the thermal power ratio, i.e. the share of thermal power generation in total power generation, which is the main source of power generation in China. According to the China Energy Statistics Yearbook, China emits more than 90% of greenhouse gases from coal and thermal power, which is 10 times more than other technologies (hydropower, nuclear and wind)^60^. Because carbon emissions from power generation are mainly produced by thermal power generation, we assumed in this paper that other energy generation does not produce carbon emissions. According to the National Bureau of Statistics, the national electricity generation in 2019 and 2020 was 750,342,428 million kW h and 777,960 million kW h, respectively, while thermal power generation was 5,220,150 million kW/h and 5,330,250 million kW h, making the share of thermal power generation in 2019 and 2020 69.57% and 71.04%, respectively^61^. ${\text{t}}_{{\text{M}}}$ was the discount factor between fuel coal and standard coal; the parameter value used was 1.07. ${\text{i}}_{{{\text{c}}h}}$ was the charge efficiency, i.e. the ratio of the electrical energy input to the power battery to the electrical energy from the grid, based on the initial charge/discharge efficiency of BEVs batteries calculated by Yang et al.^62^, which is 98%. ${\text{i}}_{{{\text{tr}}}}$ was line loss rate, which is the percentage of power supply, and the amount of power supply according to the data released by the National Energy Administration of China. The integrated line loss rates of the grid in 2019 and 2020 were 5.93% and 5.62%, respectively^61^. According to Eq. (4), the carbon emission factors for electricity in 2019 and 2020 were 0.6818506 kg/(kW h) and 0.6776147 kg/(kW h), respectively.

### Supplementary Information

Supplementary Information.

## Data Availability

The data supporting the findings of this study are under the custodianship of the corresponding author, Jin Liu. These data are subject to access restrictions as they were used under a specific license for this study and are not publicly available. However, access to the data can be granted upon reasonable request and with the express permission of Jin Liu.
