# Supplementary material for: Impact of battery electric vehicle usage on air quality in three Chinese first-tier cities
Source: Sci Rep. 2024 Jan 2;14:21. doi: 10.1038/s41598-023-50745-6 (PMC10761960; doi:10.1038/s41598-023-50745-6)
Supplement: Supplementary file 1 — Supplementary Information. [file 41598_2023_50745_MOESM1_ESM.zip › Supplementary material/Supplementary Information.docx]

**Influence of Travel in Battery Electric Vehicles on Air Quality in Three China's first-tier cities**

**Supplementary information 1: Literature review**

Previous studies have analysed the emission reduction benefits of electric vehicles from different research methods, research perspectives and research scenarios. These studies have considered the different stages of EVs, including the production stage, use stage, and recycling stage. Supplementary Table 1 summarises the literature review of research related to EV emission reduction in Nature Journal. In terms of research methodology, most of the articles used predictive models to analyse the benefits of EV emission reduction under different scenarios. Some articles used a mixed analysis approach, combining descriptive analysis and empirical models, to examine the historical and current emission reduction benefits of EVs, and to explore the temporal and regional differences in the emission reduction benefits of EVs, etc. In terms of research perspectives, there are mainly a whole life cycle perspective and a use phase perspective, both of which have their own advantages, disadvantages and applicability. In researching carbon emission models, some studies have analysed the emission reduction benefits of electric vehicles from a whole life cycle perspective, comparing the differences in emission reductions with those of fuel vehicles^1,2,3,4^. For example, Abdul-Manan et al. compared the emission reduction benefits of two-wheeled electric vehicles and four-wheeled electric vehicles, which are similar in weight, size, etc., compared to fuel vehicles, but use three different power sources, namely diesel, petrol and electricity, by using a life-cycle model^4^. However, LCAs only provide a vehicle-level perspective, but fail to examine the large-scale impacts of vehicle electrification^5^. There is also literature that focuses on the differences in emission reductions between electric and fuel vehicles during the driving phase^6,7^. For example, Jenn used 13 million charging data from charging network providers from 2014-2018, and combined data from multinational companies such as Uber and Lyft containing 1.4 million trips from San Francisco, Los Angeles and San Diego from 2017-2018, to consider scenarios where electric vehicles are used in ride-hailing services, by calculating the difference in carbon emissions during the driving phase. found that the potential environmental and emissions reduction benefits of electric vehicles for ride-hailing services in California are approximately three times greater than those of regular vehicles^6^. However, these studies were either limited to small actual data and sample sizes or to a single vehicle type, making it difficult to reveal the overall characteristics and heterogeneous effects of the relationship between driving and air quality. In terms of research scenarios, including the analysis of different EV types and the analysis of different levels of fleet electrification, these research scenarios refine the variability and reflect the uncertainty of the emission reduction benefits of EVs.

From the perspective of research content, compared with Liang et al.'s study^1^, although this study also focuses on the electrification scenario of representative cities in the economically developed regions of Jing-Jin-Ji, Yangtze River Delta, and Pearl River Delta in China, it uses micro-level data, including detailed and real micro information such as the number of vehicles, driving distance, driving time, battery capacity, etc. The study considers the emission reduction benefits of multiple types of electric vehicles in different driving stages, and also expands the research on the environmental benefits of electric vehicles replacing fuel vehicles for travel, explores the relationship between pollutant emissions and actual air quality, and focuses the analysis on the usage stage of electric vehicles rather than the entire life cycle. Although Jenn^6^ also used micro-level data to examine the emission reduction benefits of electric vehicles replacing gasoline vehicles during driving, this study differs from Jenn's research in that it uses large-scale, real-world data with a more extensive and diverse sample. This study not only examines ride-hailing trips but also the emission reduction characteristics of private passenger cars and rental passenger cars, with a more comprehensive perspective. It also investigates the impact of electric vehicle trips on various air pollutants and their actual impact on air quality. In terms of research content, this study first calculates the emission reduction benefits of replacing gasoline vehicles and then focuses on the impact of trips on air quality, extending the discussion to the specific effects of trips on different air pollutants. It analyzes the differentiated impact of trips of different models and categories on air quality, providing the emission reduction effects and environmental benefits of electric vehicle trips in various scenarios, which has significant policy implications.

Overall, as shown in Supplementary Table 1, the literature review demonstrates that there are diverse perspectives on emissions reduction research of electric vehicles. However, due to the limited number and level of detail of samples, it is difficult to reveal the actual emissions reduction benefits of electric vehicles and their impact on air quality. The operational electricity consumption and related greenhouse gas emissions depend on operating conditions and the battery charging/discharging process12. To reduce individual biases with limited data resources, reveal the relationship between travel characteristics and air quality, and explore the heterogeneity of travel effects, this study analyzes the usage patterns of battery electric vehicles (BEVs) using large-scale real-world data resources to assess the emissions reduction benefits of replacing traditional fuel vehicles. Based on actual vehicle data from January 2019 to October 2020 in three cities, Beijing, Shanghai, and Shenzhen, the carbon emissions reduction benefits and environmental benefits of electric vehicle driving are calculated. Since carbon emissions are not produced during driving, the carbon emissions produced by electricity generation corresponding to the energy consumption during driving are used as the carbon emissions of the electric vehicle. By comparing the carbon emissions of electric vehicles and traditional fuel vehicles with the same driving mileage, the impact of internal and external factors such as travel time, vehicle age, road conditions, and weather are eliminated.

**Supplementary Table 1.** Review of relevant literature

| **Article Name** | **Author-Journal-Year** | **Region** | **Research type** | **Alternate scenarios** | **Analysis of scenarios** |
| --- | --- | --- | --- | --- | --- |
| Air quality and health benefits from fleet electrification in China | Xinyu Liang et al.-Nature sustainability-2019 | China | Predictive simulations | Full life cycle | Evaluate the air quality impacts from multiple scenarios by considering various BEVand PHEV penetration levels in Beijing，Shanghai and Guangzhou |
| Emissions benefits of electric vehicles in Uber and Lyft ride-hailing services | Alan Jenn-Nature energy-2020 | Los Angeles & San Diego | Mixed methods（Descriptive analysis，Empirical analysis & Predictive simulations） | Driving phase | Analyze the carbon emission differences between electric ride-hailing and regular fuel ride-hailing vehicles with similar mileage and characteristics in the driving stage |
| Electrification of light-duty vehicle fleet alone will not meet mitigation targets | Alexandre Milovanoff et al.-Nature climate change-2020 | United States | Predictive simulations | Full life cycle | Predict the proportion of electric vehicles in the total road fleet in the United States under five scenarios, and calculate the corresponding environmental benefits from a full life cycle perspective |
| Decarbonization scenarios and carbon reduction potential for China’s road transportation by 2060 | Quanying Lu et al.-Nature urban sustainability-2022 | China | Mixed methods（Empirical analysis & Predictive simulations） | Full life cycle | Calculate and predict the penetration rate of NEV stock in different types of vehicles under different scenarios, and study the carbon emissions of the transportation sector under the three vehicle electrification scenarios |
| Gross polluters and vehicle emissions reduction | Matteo Böhm et al.-Nature sustainability-2022 | London,Rome & Florence | Predictive simulations | Driving phase | Study how the electrification of a certain share of vehicles would change the emissions on the roads of the London,Rome and Florence |
| Electrifying passenger road transport in India requires near-term electricity grid decarbonisation | Amir F. N. Abdul-Manan et al.-Nature communications-2022 | India | Mixed methods（Descriptive analysis，Empirical analysis & Predictive simulations） | Full life cycle | Compare the benefits of emission reduction by using a life-cycle model for two- and four-wheelers that are similar in weight, size, etc., but use three different power sources (diesel, gasoline, electricity) |

**Supplementary information 2：Research hypothesis**

**Research hypothesis 1：BEVs replacement for fuel vehicles can effectively reduce urban air pollution.**

Specifically in relation to emissions reduction, most studies concluded that the promotion of new energy vehicles can reduce pollutant emissions and improve environmental quality, and pointed out that BEVs play an important role in relieving the pressure of oil shortages, reducing vehicle emissions and the sustainable development of the transport industry^8^. Electric vehicles use cleaner energy and emit almost zero emissions while driving, making them an important solution to energy consumption and pollution problems in the transport sector in many countries. A number of studies have explored the emission reduction benefits of electric vehicle applications from both an empirical and policy perspective. For example, Alexandre Milovanoff et al^9^. found that if the US deployed over 350 million BEVs on the road in 2050 (90% of the fleet), CO_2_ emissions from light vehicles can be controlled to a level consistent with the prevention of global warming by more than 2℃. Liang et al. ^1^ the benefits of the development on air quality and human health in China and key regional urban agglomerations are systematically revealed, and the reduction of PM_2.5_ concentrations by different electrification scenarios is quantitatively assessed. Kunlun Wang and Leven J. Zheng^10^ using the China Promotion Project as a natural experiment to explore the causal effect of promoting new energy vehicles on reducing carbon intensity, it was found that the adoption of new energy vehicles reduced carbon intensity and had a significant emission reduction advantage. Wang et al.^11^ evaluated the effectiveness of the new energy vehicle policy, pointing out from an economic perspective that the economic benefits of the promotion of new energy vehicles far outweigh the costs, and the promotion of new energy vehicles can significantly reduce PM2.5 concentration and reduce internal medical diseases and deaths.

**Research hypothesis 2：The magnitude of air pollution improvement varies between types and uses of BEVs replacing fuel vehicles.**

As there are significant differences in the operating power, travel functions and characteristics of BEVs for different uses, they produce different carbon emissions and therefore may have different levels of improvement in air pollution. In addition, the size of the BEVs model directly affects the use, frequency of use, carbon emission power and total volume of BEVs. In general, the larger the vehicle, the heavier it generally is, the greater its displacement and the more energy it releases per unit of time from the engine, so the more significant the emission reduction effect of the same type of BEVs replacement^12^. Therefore, this study will discuss the differential impact of BEVs on air pollution for different vehicle models (A, B, C, SUV, MPV) separately. In addition, compared to private passenger cars, rental passenger cars spend a lot of time outdoors and have a high charging demand, regardless of the season and day and night, as well as a high driving demand^13,14^, moreover, frequent stops and multiple starts of the vehicle not only consume a lot of electricity, but also accelerate the ageing of the battery, further reducing efficiency and increasing energy consumption, thus instead increasing air pollutants and worsening air quality. Some studies have shown an increase in CO_2_, PM, SO_2_ and NO_x_ emissions after the electrification of taxis under the existing grid structure^14^. In contrast, although the travel characteristics of a for-hire passenger car are similar, a for-hire passenger car consumes 36% more fuel and produces 44% more hydrocarbons than a for-hire passenger car^15^. Consequently, there may also be differences in the magnitude of air pollution improvements for different uses (private cars, taxis, internet taxis) of BEVs in place of fuel car trips.

**Research hypothesis 3：Regional heterogeneity in the environmental benefits of BEVs replacement for fuel vehicles.**

Although BEVs offer significant emission reductions, they rely on electricity for their exercise, the production of which also consumes large amounts of fossil fuels. On the one hand, the electricity used for BEVs in China is dominated by coal, and the high pollution from coal-fired power plants may offset the potential environmental benefits^16^. On the other hand, the distribution characteristics of the grid make BEVs emissions significantly spatially heterogeneous^17^. This is due to differences in the composition of electricity in different regions, with the higher the share of coal generation, the higher the carbon emissions per unit of electricity generated, leading to possible differences in the environmental benefits of BEVs in different regions under similar driving characteristics. In addition, there is a positive correlation between the intermediary centrality of cities and road emissions, with more economically developed places also tending to have higher traffic volumes and consequently more polluting emissions^7^. It is thus necessary to explore whether there are differences in the substitution of BEVs for fuel vehicles under different geographical areas.

In recent years, people's recognition and frequency of use of new energy vehicles have increased significantly. Some researchs and studies have shown that in China, the performance of electric vehicles in terms of mileage, number of trips and other aspects is not significantly different or even better than gasoline vehicles. We compared an article about the actual operation of all cars (Most are fuel vehicles) in China^18^, a survey report about the actual operation of electric vehicles in China^19^, and our research data on the driving data of fuel and electric vehicles in Beijing, Shanghai and Shenzhen. The commuting distances and per vehicle AVKT in three cities are shown in Supplementary Table 2. As we can see, the annual total mileage of new energy vehicles in our research data is similar to that of other fuel models, and the average mileage is even higher, avoiding the range anxiety problem of electric vehicles replacing fuel vehicles. In addition, compared with the actual operation of electric vehicles surveyed by Autohome, our research data is basically consistent. The above information indicates to a certain extent that electric vehicles have good alternatives.

**Supplementary Table 2.** The commuting distances and per vehicle AVKT in three cities

| Region | Commuting distance (one way, km) | | | Per vehicle AVKT (km) | | |
| --- | --- | --- | --- | --- | --- | --- |
|  | Shiqi Ou et al. | Survey report | Our study | Shiqi Ou et al. | Survey report | Our study |
| Beijing | 13.58 | 22.17 | 22.03 | 12090.07 | 23725.00 | 11870.52 |
| Shanghai | 13.47 | 22.17 | 35.28 | 12222.88 | 23725.00 | 23180.32 |
| Shenzhen | 11.30 | 22.17 | 60.42 | 13568.76 | 23725.00 | 9716.08 |

**Supplementary information 3: Study variables**

The descriptive statistics of the relevant variables involved in the model are shown in Supplementary Table 3. In the sample of this study, the average monthly number of trips made by BEVs was approximately 38, with at least one trip per day, and the highest monthly trip was 2,067. In terms of mileage travelled, BEVs travelled an average of 503 km per month and an average of approximately 17 km per day. During the study period, the average value of the Air Quality Index (AQI) for the three cities was 59.92, which was generally good, but the highest value was 86.79 and the lowest value was 18.45, which was a large difference. Other variables related to air quality such as PM_2.5_, PM_10_, CO, SO_2_, NO_2_ and O_3_ show similar distribution characteristics.

**Supplementary Table 3.** Descriptive statistics for each variable

| variable | N | mean | p50 | sd | min | max |
| --- | --- | --- | --- | --- | --- | --- |
| AQI | 1826095 | 59.92 | 64.22 | 15.53 | 18.45 | 86.79 |
| PM_2.5_ | 1826095 | 35.47 | 35.68 | 12.02 | 7.518 | 63.23 |
| PM_10_ | 1826095 | 54.86 | 57.77 | 16.89 | 16.79 | 103.2 |
| CO | 1826095 | 638.7 | 621.4 | 128.9 | 405.9 | 941.4 |
| SO_2_ | 1826095 | 4.355 | 3.991 | 1.535 | 2.581 | 8.986 |
| NO_2_ | 1826095 | 30.90 | 26.39 | 9.408 | 17.03 | 63.89 |
| O_3_ | 1826095 | 66.64 | 63.68 | 26.23 | 22.91 | 116.9 |
| BEVtrips | 1826095 | 36.63 | 26 | 37.78 | 1 | 2067 |
| Mileage | 1826095 | 934.5 | 503 | 1605 | 0.100 | 652372 |
| lngdp | 1826095 | 9.813 | 9.893 | 0.553 | 8.654 | 10.56 |
| Industry | 1826095 | 0.199 | 0.155 | 0.0874 | 0.122 | 0.401 |
| lnpopula | 1826095 | 7.623 | 7.197 | 0.687 | 7.196 | 9.086 |
| Govern | 1826095 | 0.517 | 1 | 0.500 | 0 | 1 |
| lntemp | 1826095 | 2.524 | 3.020 | 1.088 | -0.693 | 3.418 |
| lnhumi | 1826095 | 4.045 | 4.060 | 0.261 | 3.367 | 4.454 |
| lnprecip | 1826095 | 3.672 | 3.936 | 1.436 | 0 | 6.064 |
| lnsun | 1826095 | 5.242 | 5.280 | 0.271 | 3.789 | 5.641 |
| lnwind | 1826095 | 1.036 | 1.014 | 0.235 | 0.658 | 1.718 |

In China, models are differentiated according to their displacement, and generally the A00, A0, A, B and C classes are referred to as micro cars, small cars, compact cars, medium cars and medium to large cars respectively, and SUVs and MPVs are referred to as sport utility vehicles and multi-purpose vehicles respectively, and the vehicle weight, wheelbase, width, length and representative models of each model are shown in Supplementary Table 4. According to formula (1), the 100 km of different models Fuel consumption is calculated as shown in Supplementary Table 5.

**Supplementary Table 4.** Characteristics of the different models and representative models

| Type | Weight/kg | Wheelbase/mm | Width/mm | Hatchback conductor/mm | Three-compartment conductor/mm |
| --- | --- | --- | --- | --- | --- |
| A00-type | 850-1050 | 2300-2450 | 1600-1675 | 3400-3700 |  |
| A0-type | 1050-1250 | 2450-2600 | 1675-1750 | 3700-4000 | 4100-4400 |
| A-type | 1250-1450 | 2600-2750 | 1750-1825 | 4000-4300 | 4400-4700 |
| B-type | 1450-1650 | 2750-2900 | 1825-1900 |  | 4700-5000 |
| C-type | 1650-1850 | 2825-2975 | 1860-1940 |  | 4900-5100 |
| D-type | 1850-2050 | 2900-3050 | 1900-1975 |  | 5000-5300 |
| SUV-type | 1000-3000 | 2620-2950 | 1700-2150 |  |  |
| MPV-type | 1200-3000 | 2850-3400 | 1650-2100 |  |  |

**Supplementary Table 5.** Fuel consumption per 100 km for different models

| Fuel car models | BEV models | 2019.1-2019.12 | 2020.1-2020.10 |
| --- | --- | --- | --- |
| Mini cars | A00 | None | None |
| Small cars | A0 | 7.92229753 | 7.99227886 |
| Compact cars | A | 9.22495720 | 9.21497064 |
| Medium-sized cars | B | 10.85407354 | 10.93105325 |
| Medium and large cars | C | 11.80073636 | 11.63294297 |
| SUV | SUV | 10.55719048 | 10.56834258 |
| MPV | MPV | 10.84547650 | 11.07811325 |

**Supplementary Information 4: Environmental benefits of electric vehicle replacement for fuel vehicle travel in three cities**

For the environmental benefits of replacing fuel car trips with EVs, we built an empirical model to verify why increased EV trips reduce pollutant emissions. We think that if the pollutants generated by EV are lower than those emitted by gasoline vehicles in the same mileage, then EV travel is more beneficial, thus demonstrating that there is indeed a causal relationship between EV travel and the environment. Therefore, we calculate the difference between fuel vehicle carbon emissions and electric vehicle carbon emissions for the same mileage, presenting a picture of different pollutants for the three cities in total, for different regions and for different vehicle models. We calculate fuel vehicle carbon emissions by referring to the IPCC's gasoline carbon emission factor and the 100km fuel consumption from the Car Owners' Home website; the reference PRC national standard (GB/T 37340-2019) calculates the carbon emissions of electric vehicles and find that the CO_2_ emissions and energy consumption from electric vehicle trips are much lower than those from fuel vehicles at the same mileage, which also validates our hypothesis. We then looked specifically at how much less various air pollutants would be produced by a fuel car at the same mileage.

In order to rule out the impact of the coronavirus pandemic and avoid the impact of missing months in 2020, we calculated the total emission reductions for 2019 based on the equations and parameters in the COPERT model specification, specifically for the hot phase, cold phase and evaporation phase.

**Supplementary Information 4.1: COPERT Model Introduction**

COPERT Model originated from a study of road motor vehicle emission factors carried out by the European Commission for the Environment (European Environment Agency, EEA). The COPERT model can calculate emissions from the road transport sector for both conventional (CO_2_, CO, NO_x_, VOC, PM) and non-conventional pollutants (N_2_O, NH_3_, SO_2_, etc.), as well as the energy consumption of road transport. Depending on the calculation method, the emission calculations for the above pollutants can be divided into two groups. The first group is based on different traffic conditions and engine conditions with corresponding specific emission factors (including the calculation of CO, NO_x_, VOC, PM); the other group is dependent on energy consumption (including CO_2_ and SO_2_), which is calculated in the same way as the first group. The other group of pollutant emissions is dependent on energy consumption (including CO_2_ and SO_2_), which is calculated in a similar way to the first group, based on a specific energy consumption factor.

The COPERT model has been widely used by scholars in China and abroad for the study of emissions from the road traffic sector. In addition to the use of the COPERT model in European countries to study road traffic energy consumption and pollutant emissions, many scholars have used the COPERT model to study pollutant emissions from the road traffic sector in China. For example, Lang et al.^20^ used the COPERT model to analyze the historical trend of air pollutant emissions from motor vehicles in China from 1999-2011; Susan C. Anenberg et al. used the COPERT model to calculate and predict air pollutants for diesel vehicles in 2015 and 2040 in the EU28, China, USA, India, Brazil and other countries^21^; the COPERT model was applied to calculate the Chinese motor vehicle emission factors, and by comparing the Chinese motor vehicle emission factors obtained from the COPERT model, MOBILE model and bench testing, it was found that the motor vehicle emission factors obtained from the COPERT model were closer to the actual Chinese motor vehicle emission situation.

**Supplementary Information 4.2: COPERT basic idea of the model**

In summary, the COPERT model considers three different sources of pollutant emissions from motor vehicles: namely, engine emissions during the heat stabilisation phase (known as thermal emissions); emissions during the warm-up phase (known as cold start emissions); and fuel evaporation emissions. There is a substantial difference in the emission performance of vehicles under hot stable and cold emission conditions, so it is essential to distinguish between hot stable and cold emissions. The cold emission phase emits most pollutants at concentrations several times higher than the hot emission phase, so the cold emission phase calculation process needs to account for additional emissions. The calculation of emissions of non-methane hydrocarbons due to fuel evaporation also has a different calculation mechanism to that of hot and cold emissions. The total motor vehicle emissions calculation can be expressed through equation 2-1.

$E_{\mathrm{total}}=E_{hot}+E_{cold}+E_{\mathrm{evaporation}}$ (2-1)

Where $E_{\mathrm{total}}$ indicates total emissions of a pollutant; $E_{hot}$ indicates emissions during the thermal stabilisation phase; $E_{cold}$ indicates cold start emissions during the warm-up phase; $E_{\mathrm{evaporation}}$ indicates fuel evaporation (non-methane VOC emissions caused by petrol light duty vehicles only).

**4.2.1 Thermal stabilisation phase emissions**

Heat stabilisation phase emissions, i.e. emissions produced under engine heat stabilisation and exhaust aftertreatment conditions. Thermal emissions depend on a number of factors, including the speed (or type of road) at which the motor vehicle is driven, the age of the vehicle, the engine type, technology and weight. They are calculated using the following formula:

$E_{hot;i,j,k}=N_{j}\times{VKT}_{j,k}\times e_{hot;i,j,k}$ (2-2)

Where $E_{hot;i,j,k}$ denotes the thermal emissions of pollutant i, k denotes the type of road and j denotes the type of motor vehicle; $N_{j}$ denotes the number of motor vehicles; ${VKT}_{j,k}$ denotes the average annual mileage of motor vehicles; $e_{hot;i,j,k}$ denotes the emission factor of the pollutant.

The COPERT model classifies motor vehicles into five main categories: Passenger Cars, Light Duty Vehicles, Heavy Duty Vehicles, Buses and Motorcycles. All vehicles in this study are Passenger Cars. The breakdown is based on the type of fuel, displacement or weight, and the emission standards met. The vehicle types and corresponding displacements in this sample are: A00 models <1.0L; A0 models 1.0-1.3L, A models 1.3-1.6L, all set in the CC <1.41 range; B models 1.6-2.4L, set in the 1.41 < CC <2.01 range; C models: 2.3-3.0L; SUVs and MPVs: indeterminate, set in the CC >2.01 interval.

Due to data limitations, only data on the distribution of mileage travelled on national roads and motorways could be found, so this study only considers the scenario of electric vehicles travelling on national roads and motorways instead of fuel vehicles, assuming that all vehicles only travel on national roads and motorways. The proportion of total mileage travelled on national roads and highways in the three cities was then used as the proportion of actual mileage travelled on national roads and highways by the sample vehicles.

The COPERT model provides emission factors for CO, NO_x_, VOC, PM and energy consumption factors for different engine displacements, fuel types and emission control standards, with driving speed as the independent variable, and a large number of experimental measurements have been carried out to fit the corresponding emission factor calculation formulae for each type of motor vehicle. The COPERT model provides CO, NO_x_, VOC, PM emission factors and energy consumption factors for the corresponding conditions, and we use the COPERT model The software defaults to the Euro V standard, which corresponds to the Chinese National V standard, and the specific calculation results are shown in the Supplementary Data 5.

**4.2.2 Cold start emissions**

Cold start emissions are the emissions produced during the warm-up phase when a motor vehicle is started. In principle, all motor vehicle types produce cold start emissions. However, given the availability of data and the reasonableness of the results, the COPERT model can only measure cold start emissions for Passenger Cars and Light Duty Vehicles, and ignores the effect of vehicle age. Cold start emissions are related to driving habits (the average length of each journey, which affects how often the vehicle is started) and the ambient temperature (which affects the time and distance to start and warm up). The specific formula is as follows:

$E_{cold;i.j}=\beta_{j}\times N_{j}\times{VKT}_{j}\times e_{hot;i,j}\times\left( e^{cold}/e^{hot} | i,j-1 \right)$ (2-3)

Where $E_{cold;i.j}$ denotes emissions during the cold start phase, i denotes a different pollutant and j denotes the motor vehicle type; $\beta_{j}$ denotes the proportion of miles travelled with a cold engine or catalyst operating below the starting ignition temperature; $N_{j}$ denotes the number of motor vehicles; ${VKT}_{j}$ denotes motor vehicle miles travelled; $e_{hot;i,j}$ denotes the thermal emission factor; $e^{cold}/e^{hot}$ denotes the cold emissions to thermal emission factor ratio, which is related to motor vehicle type j. We also use the default Euro V standard of the COPERT model software, which corresponds to the Chinese National V standard, and the specific calculation results are shown in the Supplementary Data 5.

The parameter $\beta$ is related to the ambient temperature (average monthly temperature) and the average distance per journey. The specific formula for the parameter $\beta$ is as follows:

$\beta=0.6474-0.02545\times I_{\mathrm{trip}}-（0.00974-0.000385\times I_{\mathrm{trip}}）\times t_{a}$ (2-4)

Where $I_{\mathrm{trip}}$ is the average distance travelled per leg; $t_{a}$ is the average monthly ambient temperature.

**4.2.3 Fuel evaporation emissions**

There are three main sources of fuel evaporation: daily emissions (Diurnal emissions), heat soak emissions (Hot soak emissions) and running losses. Due to a lack of data, the COPERT model is limited to calculating non-methane VOC emissions from petrol light duty vehicles. Because evaporative emissions are temperature sensitive, the model measures them on a monthly basis.

Daily emissions cause evaporative emissions, which are caused by the expansion of vapour in the fuel tank evaporating as the ambient temperature rises during the day. In the absence of an evaporation control system, some fuel vapour is vented to the atmosphere. At night, when the temperature drops, the fuel vapour is mixed with fresh air and drawn into the tank through the exhaust port. This leads to a reduction in the concentration of hydrocarbons in the vapour space above the liquid petrol, which subsequently leads to additional evaporation.

Hot soak emissions are emissions caused when the engine is switched off. The heat from the engine and exhaust system raises the temperature of the non-flowing fuel system, causing fuel evaporation. The float is the main source of heat soak evaporation.

Running losses are the evaporation of petrol from the fuel tank during the running phase of the vehicle. Running losses are more severe in high ambient temperature conditions. Under the combined effect of high ambient temperatures and high temperature exhaust systems, heated fuel flows back from the engine to the fuel tank, generating significant fuel evaporation emissions.

The formula for calculating evaporative fuel emissions is as follows:

$E_{\mathrm{vapour};VOC;j}=365\times N_{j}\times(e^{d}+S^{c}+S^{\mathrm{fi}})+R$ (2-5)

Where $E_{\mathrm{vapour};VOC;j}$ denotes VOC emissions due to fuel evaporation; $N_{j}$ denotes the number of motor vehicles of type j; $e^{d}$ denotes the daily evaporation emission factor, which is related to ambient temperature, temperature difference and fuel vapour pressure; $S^{c}$ denotes the heat soak emission factor for motor vehicles; $S^{\mathrm{fi}}$ denotes the heat soak emission factor for motor vehicles fitted with fuel injectors; and $R$ denotes the running loss.

$$S^{c}=(1-q)\times(p\times x\times e^{s,hot}+w\times x\times e^{s,warm})$$

$$S^{fi}=q\times e^{fi}\times x$$

$$R=M_{j}\times(p\times e^{r,hot}+w\times e^{r,warm})$$

$$x=M_{j}/(365\times I_{\mathrm{trip}})$$

Where $q$ denotes the proportion of petrol-powered vehicles equipped with fuel injection; $p$ denotes the proportion of trips completed when the engine is hot (depending on the average monthly ambient temperature); $w$ denotes the proportion of trips completed when the engine is cold or warm (shorter trips) or when the catalyst is below its ignition temperature; $x$ denotes the average number of trips per day in a year for a vehicle; $e^{s,hot}$ denotes the average emission factor for hot soak emissions (depending on the fuel volatility RVP); $e^{s,warm}$denotes the average emission factor for cold soak and warm soak emissions (depending on fuel volatility RVP and average monthly ambient temperature); $e^{fi}$ denotes the average emission factor for hot and warm soak emissions from petrol-powered vehicles equipped with fuel injection; $e^{r,hot}$ denotes the average emission factor for hot running losses from petrol vehicles (depending on fuel volatility RVP and average monthly ambient temperature); $e^{r,warm}$ denotes the average emission factor for hot running losses from petrol-powered vehicles (depending on fuel volatility RVP and average monthly ambient temperature); $M_{j}$ denotes the average emission factor for hot running Average emission factor for losses from the thermal operation of petrol-powered vehicles (depending on fuel volatility RVP and average monthly ambient temperature); denotes total annual mileage of petrol-powered vehicles in category j.

As mentioned earlier, evaporation losses depend on the technology used, fuel characteristics and average ambient temperature. Supplementary Table 6 shows the basic emission factors necessary to apply the method to uncontrolled and controlled vehicles (based mainly on CONCAWE 1987.1990a, Heine 1987, US EPA 1990) ^22^. The RVP of petrol vapour pressure (RVP) varies from season to season, in China the RVP is 85 KPa in winter and spring (1 November to 30 April) and 65 KPa in summer and autumn (1 May to 31 October) ^23^. The proportion w of strokes completed by cold and warm engines is related to the β parameter used in the calculation of cold start emissions: both depend on the ambient temperature. In the absence of better data, w and β are assumed to be approximate. Again we use the default Euro V standard of the COPERT modelling software, which corresponds to the Chinese National V standard, and the results of the calculations are shown in the Supplementary Data 5.

**Supplementary Table 6.** Summary of emission factors used to estimate evaporative emissions from gasoline vehicles (RVP in kPa, temperature in ℃)

| Emission factors (in units) | Uncontrolled vehicles (conventional vehicles) | Vehicles equipped with automotive carbon canisters (control vehicles, post Euro 1 standard vehicles) |
| --- | --- | --- |
| Daytime emissions $e^{d}$（g/day） | $9.1\times\exp\left( 0.0158\left( \mathrm{RVP}-61.2 \right)+0.0574\left( t_{a,min}-22.5 \right)+0.0614\times\left( t_{a,min}-11.7 \right) \right)$ | $0.2\times Vehicles without control$ |
| Warm leach discharge $e^{s,warm}$  (g/time) | $\exp\left( -1.644+0.01993RVP+0.07521t_{a} \right)$ | $0.2\times\exp\left( -2.41+0.02302RVP+0.09408t_{a} \right)$ |
| Thermal leaching emissions $e^{s,h\mathrm{ot}}$  (g/time) | $3.0042\times exp\left( 0.02RVP \right)$ | $0.3\times\exp\left( -2.41+0.02302RVP+0.09408t_{a} \right)$ |
| Warm and hot soak emissions from fuel injected vehicles $e^{fi}$  (g/time) | 0.7 | None |
| Warm loss (g/km) $e^{r,warm}$ | $0.1\times exp\left( -5.967+0.04259RVP+0.1773t_{a} \right)$ | $0.1\times Vehicles without control$ |
| Hot loss (g/km) $e^{r,hot}$ | $0.136\times exp\left( -5.967+0.04259RVP+0.1773t_{a} \right)$ | $0.1\times Vehicles without control$ |

Notes: $t_{a}=\left( t_{a,max}+t_{a,min} \right)/2$ $t_{a,rise}=t_{a,max}-t_{a,min}$

**4.2.4 Emissions based on fuel consumption**

In the COPERT model, CO_2_ and SO_2_ emissions are measured on the basis of energy consumption. The formula for calculating CO_2_ emissions is as follows:

$E_{{CO}_{2},j}=44.011\times\frac{{FC}_{j,m}}{12.011+{1.008r}_{H:C,m}}-\frac{E_{j,m}^{CO}}{28.011}-\frac{E_{j,m}^{VOC}}{13.85}-\frac{E_{j,m}^{PM}}{12.011}$ (2-6)

where:$E_{{CO}_{2},j}$ denotes CO_2_ emissions from motor vehicle category j; ${FC}_{j,m}$denotes energy consumption of motor vehicle category j, m denotes fuel type (gasoline, diesel); $r_{H:C,m}$ denotes hydrogen to carbon ratio of fuel m (gasoline about 1.8, diesel about 2.0); $E_{j,m}^{CO},{, E}_{j,m}^{VOC}$ and $E_{j,m}^{PM}$ denotes CO, VOC and PM emissions from motor vehicle category j, respectively.

SO_2_ emissions are calculated as follows:

$E_{{SO}_{2,j}}=2\times K_{S,m}\times{FC}_{j,m}$ (2-7)

Where $E_{{SO}_{2,j}}$ denotes SO_2_ emissions from motor vehicle category j; $K_{S,m}$ denotes the content of fuel m.

**Supplementary Information 5: Calculation and prediction of overall environmental benefits with sample electric vehicles**

**5.1 Calculating the actual pollutant reduction benefits**

According to the electricity consumption during the driving stage of electric vehicles, the pollution generated by power plants is calculated. Then, the results of the four major pollutants calculated by the COPERT model are subtracted from the results of the four major pollutants generated by the power plants to obtain the pollution reduction benefits of the three cities' real electric vehicle samples during travel. Power plants are classified according to the type of energy used, including: 1) thermal power plants, which use the heat generated by burning fuels such as coal, petroleum and natural gas to generate electricity; 2) hydroelectric power plants, which use the potential energy of water by guiding it to flow downstream to drive a turbine and generator; 3) nuclear power plants, which use the heat generated by nuclear fuel slow fission in a reactor to produce steam (replacing boilers in thermal power plants) to drive a turbine and generator; and 4) wind power plants, which use the rotation of large blades mounted on towers driven by wind to generate electricity. A collection of several, dozens, or even hundreds of wind turbines is called a wind farm.Among the various types of power plants mentioned above, only thermal power plants burn fossil fuels and produce carbon dioxide. China relies heavily on thermal power generation, and the fuels used in thermal power plants are mostly coal, with a small proportion of natural gas and oil. Therefore, we use coal-fired power generation as a reference to calculate the emission reduction benefits of energy conservation. According to expert statistics, data from 2006 shows that saving 1 kWh of electricity can save 0.4 kilograms of standard coal and reduce emissions of 0.272 kilograms of carbon dust, 0.997 kilograms of carbon dioxide (CO_2_), 0.03 kilograms of sulfur dioxide (SO_2_), and 0.015 kilograms of nitrogen oxides (NO_X_)^24^. However, with technological improvements, according to the China Statistical Yearbook 2019, the energy processing and conversion efficiency table uses equivalent values to calculate the standard coal coefficient for electricity conversion, with a conversion of 0.1229 kilograms of standard coal per kilowatt hour^25^.Therefore, based on a similar proportion, it can be estimated that for every 1 kWh of electricity saved in 2019, 0.1229 kg of standard coal could be saved, and the heat generated by 1 kg of standard coal is 29,270 kJ^26^.According to a study by Chen et al.^27^, emission factors based on power plant types and 2019 Power Generation by Source are shown in Supplementary Table 7 and 8. According to data from the "China Energy Statistical Yearbook 2020", in 2019, natural gas accounted for 3.1% and coal accounted for 64.7% of China's power generation sources^28^. Therefore, saving 1 kWh of electricity can reduce 0.0500/0.0008 grams of PM_2.5_, 1.22945/0.3756 grams of carbon dioxide (CO_2_), 0.5250/0.0060 grams of sulfur dioxide (SO_2_), and 0.2630/0.0629 grams of nitrogen oxides (NO_X_).

**Supplementary Table 7.** Emission factor by power plant type

| Plant type | NOx(mg/kWh) | SO_2_(mg/kWh) | CO_2_(g/kWh) | PM_2.5_(mg/kWh) |
| --- | --- | --- | --- | --- |
| Coal | 263 | 525 | 1229.45 | 50 |
| Natural gas | 62.9 | 6.0 | 375.6 | 0.8 |

**Supplementary Table 8.** 2019 Power Generation by Source Unit：%

| Country | Petroleum | Natural Gas | Coal | Nuclear Power | Hydropower | Renewable Energy | Others |
| --- | --- | --- | --- | --- | --- | --- | --- |
| China | 0.1 | 3.1 | 64.7 | 4.6 | 16.9 | 9.8 | 0.8 |
| USA | 0.5 | 38.6 | 24.0 | 19.3 | 6.2 | 11.1 | 0.3 |
| Japan | 4.3 | 35.0 | 31.5 | 6.3 | 7.1 | 11.7 | 4.1 |
| Italy | 3.6 | 44.6 | 10.5 | — | 15.9 | 23.8 | 1.6 |

The COPERT macro emission model was used in this study to investigate the benefits of electric vehicle (EV) use in Beijing, Shanghai, and Shenzhen as a replacement for fuel vehicles (FVs). Therefore, sample EV data from this study were used to predict the overall EV use in these three cities. As there were no pure EV and FV usage and ownership data for each city, this study collected the EV and motor vehicle ownership data for each city. For pure EV ownership, the estimated value was obtained by multiplying the percentage of pure EVs in the national EV fleet (310/381=0.813648294 in 2019) by the EV ownership for each city. For FV ownership, the motor vehicle ownership for each city was subtracted from the EV ownership. To further ensure the robustness of the data, the following cleaning exercise was carried out on the sample data and the cleaned data was fed into the COPERT model: (1) remove samples with less than 50 days of travel per year and (2)remove samples with an average daily distance of less than 10 km on days of travel. Assuming that EVs and FVs have the same driving range, the average emission reduction benefit per kilometer of EV travel in 2019 and 2020 was calculated based on the sample EV data, and the total emission reduction benefit for EV use in these three cities in 2019 and 2020 was calculated based on the average distance data provided in a previous study^29^. The specific results are shown in Supplementary Table 10. Due to the current use of battery electric vehicles (BEVs), the three cities have reduced their energy consumption by 34273.03895 TJ, 408.3982845 tons of PM_2.5_, 2863893.098 tons of CO_2_, and 144.5439396 tons of NO_X_. However, it will also increase SO_2_ emissions by 518.7291681 tons.

**Supplementary Table 9.** Distribution of the private passenger vehicle samples, commuting distances and per vehicle AVKT

| Area | Provincial region | Samples | Commuting distance(one way,km) | Per vehicle AVKT(km) |
| --- | --- | --- | --- | --- |
| North China | Beijing | 5659 | 13.58 | 12090.07 |
| East China | Shanghai | 3,700 | 13.47 | 12222.88 |
| South China | Guangdong | 13900 | 11.30 | 13568.76 |

**Supplementary Table 10.** Estimated emissions of individual pollutants in the three cities

|  | Beijing | Shanghai | Shenzhen | Total |
| --- | --- | --- | --- | --- |
| Number of registered motor vehicles (in 10,000s) | 636.5 | 422.6 | 349.9 | 1409 |
| Number of registered battery electric vehicles (in 10,000s) | 24.99 | 17.65616798 | 29.5191601 | 72.16043307 |
| Number of registered gasoline vehicles (in 10,000s) | 605.7925 | 400.9 | 313.62 | 1320.3125 |
| Average annual mileage (in kilometers) | 12090.07 | 12222.88 | 13568.76 | 37881.71 |
| Annual total mileage (in 10,000s of kilometers) | 7695329.555 | 5165389.088 | 4747709.124 | 17608427.77 |
| Number of sample BEVs | 79442 | 14603 | 4785 | 98830 |
| Average annual mileage of sample BEVs (in kilometers) | 5791.7739 | 10308.203 | 7256.1963 | 23356.1732 |
| Total annual mileage of sample BEVs (in 10,000s of kilometers) | 46011.01022 | 15053.06884 | 3472.08993 | 64536.16899 |
| Energy saving and emission reduction of sample BEVs (in TJ) | 1737.055417 | 586.9727356 | 128.6867946 | 2452.714947 |
| Reduction of PM_2.5_ emissions by sample BEVs (in tons) | 20.80781498 | 6.874282683 | 1.544507487 | 29.22660515 |
| Reduction of CO_2_ emissions by sample BEVs (in tons) | 145699.4953 | 48410.8905 | 10841.19199 | 204951.5778 |
| Reduction of SO_2_ emissions by sample BEVs (in tons) | -27.04676259 | -8.019134277 | -2.056425018 | -37.12232189 |
| Reduction of NO_X_ emissions by sample BEVs (in tons) | 7.745540622 | 2.371963816 | 0.226634629 | 10.34413907 |
| Energy saving and emission reduction per 10,000 kilometers (in TJ) | 0.037753038 | 0.03899356 | 0.037063209 | 0.038005277 |
| Reduction of PM_2.5_ emissions per 10,000 kilometers (in tons) | 0.000452236 | 0.00045667 | 0.000444835 | 0.000452872 |
| CO_2_ emission reduction (in tons) | 3.166622394 | 3.216014688 | 3.122382256 | 3.175763003 |
| SO_2_ emission reduction per 10,000 kilometers (in tons) | -0.000587832 | -0.000532724 | -0.000592273 | -0.000575217 |
| NO_X_ emission reduction per 10,000 kilometers (in tons) | 0.000168341 | 0.000157573 | 0.000065273 | 0.000160284 |
| Total energy consumption and emission reduction of the city (TJ) | 11404.12317 | 8415.169758 | 14845.23838 | 34273.03895 |
| Total PM_2.5_ emission reduction of the city (in tons) | 136.6075501 | 98.55356516 | 178.1735405 | 408.3982845 |
| Total CO_2_ emission reduction of the city (in tons) | 956546.9092 | 694045.6293 | 1250633.989 | 2863893.098 |
| Total SO_2_ emission reduction of the city (in tons) | -177.5675139 | -114.9667986 | -237.2280675 | -518.7291681 |
| Total NO_X_ emission reduction of the city (in tons) | 50.85105426 | 34.00580123 | 26.14444703 | 144.5439396 |

**5.2 Calculate the predicted pollutant reduction benefits.**

We predict that the proportion of electric vehicles (EVs) will significantly increase by 2030. In October 2021, the State Council issued a notice on the Action Plan for Carbon Peak by 2030, which clearly states that the proportion of new energy and clean energy-powered transportation vehicles will reach about 40% by 2030^30^. Based on this, we predict that by 2030, the proportion of EVs in China's three super-first-tier cities will reach 40%, and the number of motor vehicles will increase by 20% compared to 2019. Assuming that the annual mileage per motor vehicle remains the same as in 2019, we calculate the pollutants that can be reduced by using EVs for transportation. The specific results are shown in Supplementary Table 11. Due to the widespread use of battery electric vehicles (BEVs) in the future, the three cities of Beijing, Shanghai, and Shenzhen will collectively reduce their energy consumption by 973,701.193 TJ, 11,602.64479 tons of PM_2.5_, 81,363,550.24 tons of CO_2_, and 4,106.510853 tons of NO_x_. However, there will also be an increase of 14,737.15857 tons of SO_2_.

**Supplementary Table 11.** Estimation of pollutant emissions for the three cities in 2030

|  | Beijing | Shanghai | Shenzhen | Total |
| --- | --- | --- | --- | --- |
| Prediction of pure electric vehicle ownership in 2030 / 10,000 units | 305.52 | 202.848 | 167.952 | 676.32 |
| Total energy consumption and emission reduction in 2030 (TJ) | 139450.5932 | 96680.11525 | 84463.36098 | 973701.193 |
| Total PM_2.5_ emission reduction in urban areas in 2030 (tons) | 1670.448802 | 1132.26118 | 1013.734889 | 11602.64479 |
| Total CO_2_ emission reduction in urban areas in 2030 (tons) | 11696737.39 | 7973744.245 | 7115598.108 | 81363550.24 |
| Total SO_2_ emission reduction in urban areas in 2030 (tons) | -2171.310742 | -1320.829366 | -1349.731098 | -14737.15857 |
| Total NO_X_ emission reduction in urban areas in 2030 (tons) | 621.8110392 | 390.6854974 | 148.7512569 | 4106.510853 |

**Supplementary information 6: Air quality impacts of different levels of BEVs travel**

Supplementary Table 12shows that the environmental benefits of A0-, A-, B- and SUV-type electric vehicles (EVs) are significant, leading to substantial reduction in the AQI and improved air quality. However, the environmental benefits of A00-type EVs may not be significant due to their small emissions. As the weight of the EV increases, the energy consumption per 100 km also increases, and the corresponding battery capacity and range also increase, leading to an increase in carbon emissions with the increase in the weight of the vehicle^1^. Studies have shown that replacing only a large number of non-EVs with EVs can significantly reduce pollutant concentrations^31^. The impact of C-type EV travel is not significant because the number of trips is low. MPV-type EVs may significantly reduce their actual driving range due to their unique long-range requirements, as energy consumption during high-speed cruising is much higher than that during city commuting, requiring more electricity consumption^32^.

In contrast, the energy consumption during the high-speed cruising of gasoline vehicles is much lower than that for short-distance travel, so the MPV-type EV may actually increase emissions during long-distance trips, making the environmental benefits insignificant. In terms of the local sources of PM_2.5_ in the air, vehicle exhaust emissions are the largest source, accounting for approximately 22%, while coal combustion is the second-largest source of PM_2.5_, accounting for about 17% of the total PM_2.5_^33^, and coal combustion is the main source of EV energy consumption.

A-, B- and SUV-type EVs have a higher number of trips (Supplementary Table 11), so they can significantly reduce the content of PM_2.5_ in the air, while the relationship between A00- and C-type EV travel and the content of PM_2.5_ in the air is insignificant, possibly because the number of trips in these two types of EVs is relatively low in the study sample, and PM_2.5_ is mainly generated during driving. The A00- and MPV-type vehicle models may increase the content of PM_2.5_ in the air with increasing monthly travel times due to emissions and energy consumption issues.

The impact of different vehicle types on the content of PM_10_ in the air is similar to that of PM_2.5_ because their sources are similar (Supplementary Table 13). The impact of various vehicle types on the levels of CO and NO_2_ in the air is the same (Supplementary Table 14 and 16). Overall, the larger the vehicle type, the more significant the impact; the larger the vehicle volume, the more apparent the displacement benefit it brings compared to the same type of fuel vehicle. Therefore, the more trips made by BEVs in the A, B, C, SUV and SUV categories, the greater the reduction in SO_2_ in the air (Supplementary Table 15), while the smaller size of the A00 and A0 models and their thermal power generation also produce some SO_2_ emissions, leading to an overall increase in SO_2_ in the air. O_3_ mainly comes from motor vehicle exhaust and chemical production, etc., so the more trips made by all types of BEVs, the greater the reduction in SO_2_ in the air. O_3_ levels in the air are significantly reduced (Supplementary Table 17), and this environmental benefit is not significant due to the long-distance energy consumption limitations of MPVs.

**Supplementary Table 12.** Heterogeneity analysis: Different classes of BEVs

|  | (1) | (2) | (3) | (4) | (5) | (6) | (7) |
| --- | --- | --- | --- | --- | --- | --- | --- |
|  | A00 | A0 | A | B | C | SUV | MPV |
|  | lnAQI | lnAQI | lnAQI | lnAQI | lnAQI | lnAQI | lnAQI |
| BEVtrips | 0.0001 | -0.0017^***^ | -0.0016^***^ | -0.0049^***^ | 0.0001 | -0.0012^***^ | -0.0000 |
|  | (0.0002) | (0.0004) | (0.0002) | (0.0004) | (0.0006) | (0.0002) | (0.0004) |
| lngdp | 3.4545^***^ | 0.8662^***^ | 3.7384^***^ | 5.6672^***^ | 3.4553^***^ | 2.2824^***^ | 5.1442^***^ |
|  | (0.0860) | (0.0476) | (0.0406) | (0.2882) | (0.0964) | (0.0266) | (0.0589) |
| Industry | 5.6091^***^ | -2.0855^***^ | 6.0367^***^ | 10.4700^***^ | 7.9594^***^ | 2.1596^***^ | 15.1845^***^ |
|  | (0.2409) | (0.1371) | (0.1174) | (0.5871) | (0.2560) | (0.0745) | (0.1662) |
| lnpopula | -0.2205^***^ | -0.8707^***^ | -0.2867^***^ | -0.4471^***^ | 0.0723^**^ | -0.9717^***^ | 0.2677^***^ |
|  | (0.0517) | (0.0645) | (0.0099) | (0.0545) | (0.0361) | (0.0236) | (0.0103) |
| Govern | 0.1661^***^ | 0.0999^***^ | 0.2088^***^ | 0.2187^***^ | 0.1718^***^ | 0.1169^***^ | 0.2636^***^ |
|  | (0.0046) | (0.0020) | (0.0010) | (0.0074) | (0.0043) | (0.0014) | (0.0015) |
| lntemp | 0.0162^***^ | 0.0038^***^ | 0.0230^***^ | -0.0194^***^ | 0.0452^***^ | -0.0015^**^ | 0.0295^***^ |
|  | (0.0022) | (0.0014) | (0.0005) | (0.0023) | (0.0023) | (0.0006) | (0.0006) |
| lnhumi | -0.7401^***^ | -0.7683^***^ | -1.1256^***^ | -1.2598^***^ | -1.1198^***^ | -0.7255^***^ | -1.1617^***^ |
|  | (0.0257) | (0.0161) | (0.0036) | (0.0199) | (0.0145) | (0.0070) | (0.0042) |
| lnprecip | 0.0049^**^ | 0.0380^***^ | -0.0318^***^ | 0.0036 | -0.0176^***^ | 0.0282^***^ | -0.0704^***^ |
|  | (0.0021) | (0.0017) | (0.0003) | (0.0024) | (0.0018) | (0.0009) | (0.0004) |
| lnsun | -0.2046^***^ | -0.1408^***^ | -0.3323^***^ | -0.3423^***^ | -0.2643^***^ | -0.1573^***^ | -0.4057^***^ |
|  | (0.0087) | (0.0048) | (0.0011) | (0.0072) | (0.0047) | (0.0024) | (0.0019) |
| lnwind | -0.6838^***^ | -0.6637^***^ | -0.8209^***^ | -0.9653^***^ | -0.8452^***^ | -0.6549^***^ | -0.7184^***^ |
|  | (0.0156) | (0.0093) | (0.0031) | (0.0140) | (0.0093) | (0.0040) | (0.0045) |
| cons | -21.5516^***^ | 7.7442^***^ | -21.4061^***^ | -37.5944^***^ | -22.3921^***^ | -5.2152^***^ | -40.0980^***^ |
|  | (0.9003) | (0.6242) | (0.3959) | (2.7395) | (0.9682) | (0.3551) | (0.6042) |
| Month | Yes | Yes | Yes | Yes | Yes | Yes | Yes |
| City | Yes | Yes | Yes | Yes | Yes | Yes | Yes |
| N | 102966 | 84634 | 648337 | 92009 | 55612 | 567098 | 266663 |
| R^2^ | 0.8812 | 0.7571 | 0.8002 | 0.9017 | 0.7690 | 0.8434 | 0.8103 |

Note: The independent variable BEVtrips is the natural logarithm of the monthly travel frequency of electric vehicles. A BEVs’s monthly total outflow frequency is observation data. If a BEVs does not travel in a certain month, no observation data exists for it in the month. Each column represents one separate regression. Columns (1) to (8) report the results of A00, A0, B, C, SUV, MPV electric vehicles on the AQI. ‘Yes’ denotes that the corresponding fixed effects are controlled for. The month fixed effects and city-pair fixed effects are controlled for in the regressions indicated in all columns. The control variables are controlled for in the regressions indicated in all columns. Standard errors below the estimates are shown in parentheses. ^*^*P* < 0.1; ^**^*P* < 0.05; ^***^*P* < 0.01.

**Supplementary information 7：Heterogeneity regression analysis results**

**Supplementary Table 13.** Heterogeneity analysis：PM_2.5_

|  | (1) | (2) | (3) | (4) | (5) | (6) | (7) |
| --- | --- | --- | --- | --- | --- | --- | --- |
|  | A00 | A0 | A | B | C | SUV | MPV |
|  | lnPM_2.5_ | lnPM_2.5_ | lnPM_2.5_ | lnPM_2.5_ | lnPM_2.5_ | lnPM_2.5_ | lnPM_2.5_ |
| BEVtrips | 0.0007^**^ | -0.0002 | -0.0004^**^ | -0.0056^***^ | -0.0001 | -0.0010^***^ | 0.0009 |
|  | (0.0003) | (0.0005) | (0.0002) | (0.0005) | (0.0008) | (0.0002) | (0.0006) |
| lngdp | 1.3615^***^ | -0.9893^***^ | 2.9908^***^ | 4.7553^***^ | 1.2718^***^ | 0.0816^**^ | 4.4869^***^ |
|  | (0.1228) | (0.0526) | (0.0560) | (0.4147) | (0.1319) | (0.0368) | (0.0825) |
| Industry | 1.9469^***^ | -5.0790^***^ | 2.3394^***^ | 6.2247^***^ | 3.5282^***^ | -2.2030^***^ | 15.0630^***^ |
|  | (0.3301) | (0.1389) | (0.1516) | (0.7040) | (0.3289) | (0.0921) | (0.1997) |
| lnpopula | -0.3305^***^ | -1.1439^***^ | -0.5889^***^ | -0.8847^***^ | -0.0868^*^ | -1.3472^***^ | 0.3519^***^ |
|  | (0.0665) | (0.0859) | (0.0141) | (0.0712) | (0.0460) | (0.0306) | (0.0110) |
| Govern | 0.1284^***^ | 0.0280^***^ | 0.2096^***^ | 0.1959^***^ | 0.1471^***^ | 0.0566^***^ | 0.2895^***^ |
|  | (0.0065) | (0.0030) | (0.0014) | (0.0105) | (0.0062) | (0.0020) | (0.0020) |
| lntemp | 0.0141^***^ | 0.0117^***^ | 0.0092^***^ | -0.0461^***^ | 0.0466^***^ | 0.0028^***^ | 0.0139^***^ |
|  | (0.0026) | (0.0015) | (0.0006) | (0.0031) | (0.0029) | (0.0007) | (0.0008) |
| lnhumi | -0.6351^***^ | -0.5206^***^ | -1.1143^***^ | -1.2097^***^ | -0.9811^***^ | -0.4888^***^ | -1.2321^***^ |
|  | (0.0309) | (0.0219) | (0.0044) | (0.0271) | (0.0182) | (0.0092) | (0.0045) |
| lnprecip | -0.0218^***^ | 0.0069^***^ | -0.0608^***^ | -0.0023 | -0.0612^***^ | -0.0099^***^ | -0.1023^***^ |
|  | (0.0027) | (0.0020) | (0.0004) | (0.0034) | (0.0023) | (0.0011) | (0.0005) |
| lnsun | -0.3200^***^ | -0.2632^***^ | -0.4342^***^ | -0.3618^***^ | -0.4152^***^ | -0.2786^***^ | -0.4978^***^ |
|  | (0.0105) | (0.0059) | (0.0013) | (0.0091) | (0.0057) | (0.0029) | (0.0026) |
| lnwind | -0.8819^***^ | -0.7782^***^ | -1.0452^***^ | -1.2782^***^ | -0.9854^***^ | -0.7666^***^ | -0.9921^***^ |
|  | (0.0186) | (0.0127) | (0.0039) | (0.0178) | (0.0116) | (0.0054) | (0.0057) |
| cons | -1.3660 | 26.4147^***^ | -11.3370^***^ | -25.6521^***^ | -0.7750 | 17.5474^***^ | -34.2574^***^ |
|  | (1.2815) | (0.7461) | (0.5195) | (3.8428) | (1.3283) | (0.4832) | (0.8126) |
| Month | Yes | Yes | Yes | Yes | Yes | Yes | Yes |
| City | Yes | Yes | Yes | Yes | Yes | Yes | Yes |
| N | 102966 | 84634 | 648337 | 92009 | 55612 | 567098 | 266663 |
| R^2^ | 0.9083 | 0.8201 | 0.8297 | 0.9198 | 0.7927 | 0.8720 | 0.8339 |

**Supplementary Table 14.** Heterogeneity analysis：PM_10_

|  | (1) | (2) | (3) | (4) | (5) | (6) | (7) |
| --- | --- | --- | --- | --- | --- | --- | --- |
|  | A00 | A0 | A | B | C | SUV | MPV |
|  | lnPM_10_ | lnPM_10_ | lnPM_10_ | lnPM_10_ | lnPM_10_ | lnPM_10_ | lnPM_10_ |
| BEVtrips | 0.0002 | -0.0016^***^ | -0.0016^***^ | -0.0042^***^ | 0.0003 | -0.0003 | 0.0036^***^ |
|  | (0.0003) | (0.0003) | (0.0002) | (0.0004) | (0.0006) | (0.0002) | (0.0005) |
| lngdp | 6.0527^***^ | 1.6241^***^ | 4.1063^***^ | 6.3203^***^ | 5.7165^***^ | 4.3365^***^ | 6.1004^***^ |
|  | (0.0733) | (0.0709) | (0.0420) | (0.2615) | (0.0873) | (0.0287) | (0.0511) |
| Industry | 11.4988^***^ | -1.1661^***^ | 11.0581^***^ | 15.7380^***^ | 15.1790^***^ | 7.3857^***^ | 20.7080^***^ |
|  | (0.2284) | (0.2158) | (0.1227) | (0.7786) | (0.3059) | (0.0825) | (0.1902) |
| lnpopula | 0.0219 | -0.4708^***^ | 0.3759^***^ | 0.3007^***^ | 0.5615^***^ | -0.5502^***^ | 0.7446^***^ |
|  | (0.0469) | (0.0607) | (0.0103) | (0.0588) | (0.0389) | (0.0229) | (0.0146) |
| Govern | 0.2508^***^ | 0.1903^***^ | 0.2666^***^ | 0.3228^***^ | 0.2810^***^ | 0.2042^***^ | 0.3398^***^ |
|  | (0.0045) | (0.0021) | (0.0012) | (0.0085) | (0.0056) | (0.0013) | (0.0021) |
| lntemp | -0.0099^***^ | -0.0273^***^ | 0.0578^***^ | 0.0407^***^ | 0.0623^***^ | -0.0215^***^ | 0.0665^***^ |
|  | (0.0031) | (0.0021) | (0.0005) | (0.0023) | (0.0023) | (0.0010) | (0.0005) |
| lnhumi | -0.9970^***^ | -1.2915^***^ | -1.4796^***^ | -1.6524^***^ | -1.5495^***^ | -1.1341^***^ | -1.4696^***^ |
|  | (0.0303) | (0.0187) | (0.0035) | (0.0169) | (0.0152) | (0.0081) | (0.0044) |
| lnprecip | 0.0115^***^ | 0.0406^***^ | -0.0311^***^ | -0.0017 | -0.0074^***^ | 0.0310^***^ | -0.0676^***^ |
|  | (0.0021) | (0.0018) | (0.0003) | (0.0020) | (0.0016) | (0.0008) | (0.0004) |
| lnsun | -0.0424^***^ | 0.0160^**^ | -0.2540^***^ | -0.3645^***^ | -0.1882^***^ | -0.0215^***^ | -0.3434^***^ |
|  | (0.0107) | (0.0063) | (0.0014) | (0.0102) | (0.0066) | (0.0031) | (0.0022) |
| lnwind | -0.6411^***^ | -0.7356^***^ | -0.8719^***^ | -0.9139^***^ | -0.8274^***^ | -0.6773^***^ | -0.7181^***^ |
|  | (0.0203) | (0.0115) | (0.0027) | (0.0148) | (0.0084) | (0.0052) | (0.0037) |
| cons | -47.5262^***^ | -0.9294 | -29.8519^***^ | -48.4106^***^ | -46.5748^***^ | -26.8352^***^ | -53.0094^***^ |
|  | (0.8097) | (0.8603) | (0.4131) | (2.6254) | (0.9064) | (0.3578) | (0.5771) |
| Month | Yes | Yes | Yes | Yes | Yes | Yes | Yes |
| City | Yes | Yes | Yes | Yes | Yes | Yes | Yes |
| N | 102966 | 84634 | 648337 | 92009 | 55612 | 567098 | 266663 |
| R^2^ | 0.9291 | 0.8565 | 0.8647 | 0.9417 | 0.8449 | 0.8946 | 0.8681 |

**Supplementary Table 15.** Heterogeneity analysis：CO

|  | (1) | (2) | (3) | (4) | (5) | (6) | (7) |
| --- | --- | --- | --- | --- | --- | --- | --- |
|  | A00 | A0 | A | B | C | SUV | MPV |
|  | lnCO | lnCO | lnCO | lnCO | lnCO | lnCO | lnCO |
| BEVtrips | -0.0003^*^ | -0.0009^***^ | -0.0003^***^ | -0.0018^***^ | -0.0017^***^ | -0.0013^***^ | -0.0016^***^ |
|  | (0.0002) | (0.0002) | (0.0001) | (0.0002) | (0.0003) | (0.0001) | (0.0002) |
| lngdp | -0.2946^***^ | -0.4618^***^ | 0.5951^***^ | 1.6754^***^ | -0.6226^***^ | -0.5405^***^ | 0.8803^***^ |
|  | (0.0431) | (0.0231) | (0.0319) | (0.2640) | (0.0471) | (0.0149) | (0.0394) |
| Industry | -3.5421^***^ | -5.6969^***^ | -2.2033^***^ | 0.5860 | -3.3560^***^ | -4.6984^***^ | 0.9125^***^ |
|  | (0.0971) | (0.0657) | (0.0528) | (0.5696) | (0.1282) | (0.0354) | (0.0860) |
| lnpopula | 0.3687^***^ | 0.0022 | 0.0998^***^ | -0.1154^***^ | 0.3765^***^ | 0.0621^***^ | 0.2713^***^ |
|  | (0.0225) | (0.0286) | (0.0056) | (0.0267) | (0.0174) | (0.0105) | (0.0056) |
| Govern | -0.0186^***^ | -0.0454^***^ | -0.0033^***^ | 0.0207^***^ | -0.0373^***^ | -0.0392^***^ | 0.0186^***^ |
|  | (0.0018) | (0.0009) | (0.0006) | (0.0054) | (0.0022) | (0.0006) | (0.0008) |
| lntemp | -0.0879^***^ | -0.1060^***^ | -0.0942^***^ | -0.1106^***^ | -0.0871^***^ | -0.1113^***^ | -0.0837^***^ |
|  | (0.0014) | (0.0008) | (0.0003) | (0.0013) | (0.0013) | (0.0005) | (0.0003) |
| lnhumi | 0.0014 | 0.0950^***^ | 0.0298^***^ | 0.0335^***^ | -0.0099^*^ | 0.0255^***^ | 0.0595^***^ |
|  | (0.0077) | (0.0068) | (0.0016) | (0.0093) | (0.0056) | (0.0027) | (0.0023) |
| lnprecip | -0.0171^***^ | 0.0373^***^ | -0.0019^***^ | -0.0148^***^ | -0.0022^**^ | 0.0270^***^ | -0.0235^***^ |
|  | (0.0011) | (0.0011) | (0.0003) | (0.0014) | (0.0009) | (0.0006) | (0.0002) |
| lnsun | -0.0725^***^ | 0.0418^***^ | -0.0841^***^ | -0.2310^***^ | -0.0022 | 0.0144^***^ | -0.1158^***^ |
|  | (0.0031) | (0.0019) | (0.0015) | (0.0106) | (0.0036) | (0.0012) | (0.0019) |
| lnwind | -0.6682^***^ | -0.6893^***^ | -0.5031^***^ | -0.3260^***^ | -0.6728^***^ | -0.7083^***^ | -0.3941^***^ |
|  | (0.0067) | (0.0056) | (0.0023) | (0.0165) | (0.0076) | (0.0026) | (0.0025) |
| cons | 1.5252^***^ | 5.2401^***^ | -4.7595^***^ | -12.8148^***^ | 4.0919^***^ | 5.6911^***^ | -9.3762^***^ |
|  | (0.4125) | (0.2819) | (0.2638) | (2.4013) | (0.4331) | (0.1651) | (0.3537) |
| Month | Yes | Yes | Yes | Yes | Yes | Yes | Yes |
| City | Yes | Yes | Yes | Yes | Yes | Yes | Yes |
| N | 102966 | 84634 | 648337 | 92009 | 55612 | 567098 | 266663 |
| R^2^ | 0.9542 | 0.9297 | 0.9067 | 0.9604 | 0.8872 | 0.9412 | 0.8537 |

**Supplementary Table 16.** Heterogeneity analysis：SO_2_

|  | (1) | (2) | (3) | (4) | (5) | (6) | (7) |
| --- | --- | --- | --- | --- | --- | --- | --- |
|  | A00 | A0 | A | B | C | SUV | MPV |
|  | lnSO_2_ | lnSO_2_ | lnSO_2_ | lnSO_2_ | lnSO_2_ | lnSO_2_ | lnSO_2_ |
| BEVtrips | 0.0005^***^ | 0.0011^***^ | -0.0006^***^ | -0.0020^***^ | -0.0021^***^ | -0.0005^***^ | -0.0036^***^ |
|  | (0.0002) | (0.0003) | (0.0001) | (0.0003) | (0.0004) | (0.0001) | (0.0003) |
| lngdp | 3.6791^***^ | 4.4595^***^ | 5.1502^***^ | 6.1588^***^ | 3.9518^***^ | 3.8634^***^ | 4.8611^***^ |
|  | (0.0361) | (0.0376) | (0.0340) | (0.2784) | (0.0553) | (0.0166) | (0.0458) |
| Industry | 6.0310^***^ | 8.9986^***^ | 10.9201^***^ | 11.0330^***^ | 5.8294^***^ | 7.1149^***^ | 10.3821^***^ |
|  | (0.0769) | (0.1185) | (0.0800) | (0.7231) | (0.1145) | (0.0473) | (0.1123) |
| lnpopula | 0.6840^***^ | 0.5062^***^ | 0.5138^***^ | -0.2222^***^ | 0.3764^***^ | 0.5350^***^ | 0.4317^***^ |
|  | (0.0450) | (0.0461) | (0.0063) | (0.0375) | (0.0207) | (0.0128) | (0.0053) |
| Govern | 0.2075^***^ | 0.1693^***^ | 0.1571^***^ | 0.0825^***^ | 0.1431^***^ | 0.1857^***^ | 0.1709^***^ |
|  | (0.0018) | (0.0013) | (0.0005) | (0.0062) | (0.0028) | (0.0006) | (0.0008) |
| lntemp | -0.1138^***^ | -0.1175^***^ | -0.1280^***^ | -0.1532^***^ | -0.1307^***^ | -0.1314^***^ | -0.1116^***^ |
|  | (0.0018) | (0.0009) | (0.0004) | (0.0021) | (0.0016) | (0.0006) | (0.0005) |
| lnhumi | -1.0376^***^ | -0.9094^***^ | -1.0897^***^ | -1.1067^***^ | -1.0752^***^ | -1.0019^***^ | -1.0714^***^ |
|  | (0.0076) | (0.0079) | (0.0021) | (0.0123) | (0.0061) | (0.0024) | (0.0033) |
| lnprecip | -0.0012 | 0.0270^***^ | -0.0241^***^ | -0.0603^***^ | -0.0035^***^ | 0.0186^***^ | -0.0383^***^ |
|  | (0.0010) | (0.0009) | (0.0005) | (0.0030) | (0.0012) | (0.0004) | (0.0005) |
| lnsun | 0.1094^***^ | 0.1791^***^ | -0.1140^***^ | -0.3705^***^ | -0.0014 | 0.1455^***^ | -0.1954^***^ |
|  | (0.0049) | (0.0035) | (0.0029) | (0.0193) | (0.0069) | (0.0019) | (0.0033) |
| lnwind | -0.4855^***^ | -0.5471^***^ | -0.2479^***^ | -0.0683^***^ | -0.3688^***^ | -0.5164^***^ | -0.0357^***^ |
|  | (0.0083) | (0.0073) | (0.0032) | (0.0169) | (0.0083) | (0.0031) | (0.0036) |
| cons | -33.1655^***^ | -40.2422^***^ | -44.9616^***^ | -47.1161^***^ | -32.7194^***^ | -34.2112^***^ | -41.7234^***^ |
|  | (0.4085) | (0.4679) | (0.2906) | (2.5436) | (0.4445) | (0.1673) | (0.4139) |
| Month | Yes | Yes | Yes | Yes | Yes | Yes | Yes |
| City | Yes | Yes | Yes | Yes | Yes | Yes | Yes |
| N | 102966 | 84634 | 648337 | 92009 | 55612 | 567098 | 266663 |
| R^2^ | 0.9743 | 0.9137 | 0.8732 | 0.9434 | 0.9215 | 0.9421 | 0.8430 |

**Supplementary Table 17.** Heterogeneity analysis：NO_2_

|  | (1) | (2) | (3) | (4) | (5) | (6) | (7) |
| --- | --- | --- | --- | --- | --- | --- | --- |
|  | A00 | A0 | A | B | C | SUV | MPV |
|  | lnNO_2_ | lnNO_2_ | lnNO_2_ | lnNO_2_ | lnNO_2_ | lnNO_2_ | lnNO_2_ |
| BEVtrips | 0.0010^***^ | 0.0025^***^ | 0.0024^***^ | 0.0016^***^ | 0.0026^***^ | 0.0021^***^ | 0.0033^***^ |
|  | (0.0002) | (0.0003) | (0.0001) | (0.0003) | (0.0004) | (0.0001) | (0.0003) |
| lngdp | 0.0099 | -1.1175^***^ | 0.2897^***^ | 2.6523^***^ | 1.8895^***^ | 0.1899^***^ | 1.6196^***^ |
|  | (0.0437) | (0.0305) | (0.0193) | (0.2237) | (0.0711) | (0.0202) | (0.0241) |
| Industry | -6.9157^***^ | -10.7589^***^ | -6.4706^***^ | -1.5980^***^ | -1.3663^***^ | -6.4370^***^ | -1.5122^***^ |
|  | (0.1046) | (0.0817) | (0.0479) | (0.4531) | (0.2106) | (0.0507) | (0.0706) |
| lnpopula | -0.1869^***^ | -0.2870^***^ | -0.0677^***^ | -0.1103^***^ | 0.0448^***^ | -0.1589^***^ | 0.0200^***^ |
|  | (0.0286) | (0.0278) | (0.0044) | (0.0243) | (0.0171) | (0.0100) | (0.0053) |
| Govern | -0.0360^***^ | -0.0722^***^ | 0.0123^***^ | 0.0799^***^ | 0.0572^***^ | -0.0263^***^ | 0.0743^***^ |
|  | (0.0026) | (0.0012) | (0.0008) | (0.0061) | (0.0042) | (0.0010) | (0.0009) |
| lntemp | -0.0290^***^ | -0.0157^***^ | -0.0257^***^ | -0.0575^***^ | -0.0559^***^ | -0.0317^***^ | -0.0375^***^ |
|  | (0.0015) | (0.0008) | (0.0003) | (0.0025) | (0.0017) | (0.0005) | (0.0005) |
| lnhumi | 0.1318^***^ | 0.0137^**^ | 0.1178^***^ | 0.1668^***^ | -0.0371^***^ | 0.0644^***^ | 0.1130^***^ |
|  | (0.0075) | (0.0064) | (0.0017) | (0.0095) | (0.0083) | (0.0025) | (0.0023) |
| lnprecip | -0.0398^***^ | -0.0247^***^ | -0.0334^***^ | -0.0608^***^ | -0.0629^***^ | -0.0500^***^ | -0.0317^***^ |
|  | (0.0009) | (0.0006) | (0.0003) | (0.0019) | (0.0012) | (0.0003) | (0.0004) |
| lnsun | -0.0855^***^ | -0.0758^***^ | -0.0717^***^ | -0.1429^***^ | -0.1611^***^ | -0.1330^***^ | -0.0517^***^ |
|  | (0.0038) | (0.0023) | (0.0010) | (0.0070) | (0.0048) | (0.0010) | (0.0011) |
| lnwind | -0.4047^***^ | -0.5016^***^ | -0.3605^***^ | -0.3074^***^ | -0.3386^***^ | -0.4213^***^ | -0.3212^***^ |
|  | (0.0049) | (0.0038) | (0.0015) | (0.0093) | (0.0047) | (0.0014) | (0.0020) |
| cons | 6.7062^***^ | 18.9671^***^ | 3.5257^***^ | -18.2328^***^ | -11.6644^***^ | 5.4061^***^ | -9.9254^***^ |
|  | (0.4591) | (0.3756) | (0.1778) | (2.0684) | (0.7054) | (0.2115) | (0.2232) |
| Month | Yes | Yes | Yes | Yes | Yes | Yes | Yes |
| City | Yes | Yes | Yes | Yes | Yes | Yes | Yes |
| N | 102966 | 84634 | 648337 | 92009 | 55612 | 567098 | 266663 |
| R^2^ | 0.9650 | 0.9392 | 0.8941 | 0.9440 | 0.8847 | 0.9427 | 0.8697 |

**Supplementary Table 18.** Heterogeneity analysis：O_3_

|  | (1) | (2) | (3) | (4) | (5) | (6) | (7) |
| --- | --- | --- | --- | --- | --- | --- | --- |
|  | A00 | A0 | A | B | C | SUV | MPV |
|  | O_3_ | O_3_ | O_3_ | O_3_ | O_3_ | O_3_ | O_3_ |
| BEVtrips | -0.0022^***^ | -0.0080^***^ | -0.0079^***^ | -0.0079^***^ | -0.0058^***^ | -0.0043^***^ | -0.0063^***^ |
|  | (0.0003) | (0.0005) | (0.0003) | (0.0007) | (0.0009) | (0.0002) | (0.0005) |
| lngdp | 9.4908^***^ | 9.9234^***^ | 8.7155^***^ | 7.5417^***^ | 7.2649^***^ | 9.2455^***^ | 7.7819^***^ |
|  | (0.0762) | (0.0806) | (0.0463) | (0.3090) | (0.1460) | (0.0350) | (0.0795) |
| Industry | 20.2173^***^ | 17.9473^***^ | 18.8992^***^ | 17.7379^***^ | 16.6126^***^ | 17.9328^***^ | 18.7915^***^ |
|  | (0.1660) | (0.1708) | (0.0853) | (0.5663) | (0.2947) | (0.0878) | (0.1623) |
| lnpopula | 0.3031^***^ | -0.3785^***^ | 0.0688^***^ | 0.2137^***^ | 0.3947^***^ | -0.6008^***^ | 0.3630^***^ |
|  | (0.0760) | (0.0957) | (0.0102) | (0.0587) | (0.0409) | (0.0320) | (0.0098) |
| Govern | 0.3556^***^ | 0.4763^***^ | 0.3116^***^ | 0.2303^***^ | 0.2179^***^ | 0.3775^***^ | 0.2195^***^ |
|  | (0.0042) | (0.0029) | (0.0012) | (0.0081) | (0.0051) | (0.0016) | (0.0011) |
| lntemp | 0.0994^***^ | 0.0002 | 0.1506^***^ | 0.1758^***^ | 0.2118^***^ | 0.0541^***^ | 0.1878^***^ |
|  | (0.0047) | (0.0035) | (0.0007) | (0.0043) | (0.0038) | (0.0018) | (0.0008) |
| lnhumi | -1.5636^***^ | -1.5478^***^ | -1.6449^***^ | -1.8936^***^ | -1.7543^***^ | -1.5491^***^ | -1.5979^***^ |
|  | (0.0233) | (0.0171) | (0.0042) | (0.0195) | (0.0133) | (0.0060) | (0.0063) |
| lnprecip | 0.0022 | 0.0409^***^ | -0.0422^***^ | 0.0360^***^ | 0.0304^***^ | 0.0574^***^ | -0.0873^***^ |
|  | (0.0032) | (0.0029) | (0.0005) | (0.0047) | (0.0033) | (0.0015) | (0.0007) |
| lnsun | -0.1639^***^ | -0.0669^***^ | -0.0707^***^ | 0.0315^**^ | 0.0397^***^ | -0.0289^***^ | -0.1483^***^ |
|  | (0.0082) | (0.0050) | (0.0019) | (0.0139) | (0.0094) | (0.0025) | (0.0019) |
| lnwind | -1.2751^***^ | -1.1378^***^ | -1.5495^***^ | -1.8012^***^ | -1.7032^***^ | -1.2872^***^ | -1.5080^***^ |
|  | (0.0187) | (0.0116) | (0.0051) | (0.0253) | (0.0168) | (0.0047) | (0.0070) |
| cons | -79.4011^***^ | -78.5946^***^ | -70.6028^***^ | -60.2026^***^ | -59.7752^***^ | -70.9731^***^ | -64.6830^***^ |
|  | (0.7203) | (1.0357) | (0.4176) | (2.9514) | (1.3810) | (0.3818) | (0.7608) |
| Month | Yes | Yes | Yes | Yes | Yes | Yes | Yes |
| City | Yes | Yes | Yes | Yes | Yes | Yes | Yes |
| N | 102966 | 84634 | 648337 | 92009 | 55612 | 567098 | 266663 |
| R^2^ | 0.9589 | 0.9270 | 0.8268 | 0.9120 | 0.8275 | 0.9331 | 0.7548 |

**Supplementary information 8：Additional analyses**

**Supplementary Information 8.1: Consider the impact of the pandemic**

Regarding the potential impact of the COVID-19 outbreak on our findings, we acknowledge the significant influence of the pandemic on both gasoline and electric vehicle travel, and have undertaken several steps to address this issue:

**(1)Incorporation of Additional Travel Variables**: We have expanded our analysis to include variables that reflect the characteristics of electric vehicle (EV) travel, such as the number of trips (BEVtrips), miles traveled (lnmileage), and average miles per trip (Avemile). This approach allows for a more comprehensive and nuanced understanding of air quality impacts. We report the updated results in the supplementary materials, and list these tables below.

**(2)Analysis of Travel Data Distribution**: Our month-by-month analysis of EV travel variables indicates an upward trend in the average number of trips, mileage, and average mileage per EV per month, suggesting a growing popularity of EVs as an alternative to fuel vehicles. However, during the initial phase of the COVID-19 outbreak (January to March 2020), there was a noticeable decrease in EV travel variables across the three cities studied. We provide detailed data distributions in Supplementary Table 19 and Supplementary Figure 1 in the supplementary materials, corroborated by policy documents and actual travel data from Beijing during the pandemic.

**(3)Empirical Modeling Analysis**: To further validate the impact of the COVID-19 outbreak, we employed split-sample and excluded-sample regression analyses. By dividing the data into "COVID-break" (March-August 2020) and "Non-break" periods, we found that during the COVID-break, EV trips significantly improved air quality, whereas in the absence of the COVID-break, EV trips had a detrimental effect on air quality. This suggests that the reduction in fuel vehicle travel during the pandemic was a significant factor influencing our study’s findings. Additionally, when excluding data from January to March 2020, our regression results (Supplementary Table 27-29) align with these observations, confirming that the COVID-19 outbreak was a critical factor in the study’s outcomes.

**Supplementary Table 19.** Trends in the three EV travel variables

| **Time** | **BEVtrips** | **lnmileage** | **Avemile** |
| --- | --- | --- | --- |
| 2019.01 | 1.801371 | 4.941609 | 3.140238 |
| 2019.02 | 2.052844 | 5.185718 | 3.132874 |
| 2019.03 | 2.191385 | 5.350130 | 3.158745 |
| 2019.04 | 2.621178 | 5.787902 | 3.166725 |
| 2019.05 | 2.779216 | 5.971129 | 3.191913 |
| 2019.06 | 2.853660 | 5.990043 | 3.136383 |
| 2019.07 | 2.888004 | 5.998397 | 3.110393 |
| 2019.08 | 2.798249 | 5.976533 | 3.178284 |
| 2019.09 | 2.786267 | 5.980731 | 3.194465 |
| 2019.10 | 2.620300 | 5.732984 | 3.112684 |
| 2019.11 | 2.915136 | 6.012850 | 3.097714 |
| 2019.12 | 2.987467 | 5.980006 | 2.992538 |
| 2020.01 | 2.926792 | 5.960495 | 3.033703 |
| 2020.02 | 2.542616 | 5.391347 | 2.848732 |
| 2020.03 | 2.804096 | 5.834983 | 3.030887 |
| 2020.04 | 2.883805 | 5.994811 | 3.111007 |
| 2020.05 | 2.966801 | 6.132517 | 3.165716 |
| 2020.06 | 2.948882 | 6.103435 | 3.154553 |
| 2020.07 | 2.812904 | 6.116290 | 3.303386 |
| 2020.08 | 3.044441 | 6.215949 | 3.171509 |
| 2020.09 | 3.214279 | 6.348314 | 3.134035 |
| 2020.10 | 3.042237 | 6.236663 | 3.194426 |

**Supplementary Figure1.** Trends in the three EV travel variables

**Supplementary Table 20.** Dependent variable: lnAQI

|  | (1) COVID-break | | | (2) Non-COVID-break | | |
| --- | --- | --- | --- | --- | --- | --- |
|  | lnAQI | lnAQI | lnAQI | lnAQI | lnAQI | lnAQI |
| BEVtrips | -0.0006^***^ |  |  | 0.0010^***^ |  |  |
|  | (0.0000) |  |  | (0.0001) |  |  |
| lnmileage |  | -0.0003^***^ |  |  | 0.0005^***^ |  |
|  |  | (0.0000) |  |  | (0.0001) |  |
| Avemile |  |  | 0.0002^**^ |  |  | -0.0006^***^ |
|  |  |  | (0.0001) |  |  | (0.0002) |
| cons | -4.0893^***^ | -4.0786^***^ | -4.0720^***^ | -42.7648^***^ | -42.7725^***^ | -42.7896^***^ |
|  | (0.2967) | (0.2967) | (0.2967) | (0.2356) | (0.2356) | (0.2356) |
| Controls | Yes | Yes | Yes | Yes | Yes | Yes |
| Month | Yes | Yes | Yes | Yes | Yes | Yes |
| City | Yes | Yes | Yes | Yes | Yes | Yes |
| N | 673006 | 673006 | 673006 | 1153089 | 1153089 | 1153089 |
| R^2^ | 0.9788 | 0.9788 | 0.9788 | 0.7922 | 0.7922 | 0.7922 |

**Supplementary Table 21.** Dependent variable: lnPM25

|  | (1) COVID-break | | | (2) Non-break | | |
| --- | --- | --- | --- | --- | --- | --- |
|  | lnPM_2.5_ | lnPM_2.5_ | lnPM_2.5_ | lnPM_2.5_ | lnPM_2.5_ | lnPM_2.5_ |
| BEVtrips | -0.0012^***^ |  |  | 0.0017^***^ |  |  |
|  | (0.0001) |  |  | (0.0002) |  |  |
| lnmileage |  | -0.0005^***^ |  |  | 0.0006^***^ |  |
|  |  | (0.0001) |  |  | (0.0001) |  |
| Avemile |  |  | 0.0007^***^ |  |  | -0.0014^***^ |
|  |  |  | (0.0001) |  |  | (0.0003) |
| cons | -28.2789^***^ | -28.2547^***^ | -28.2472^***^ | -36.6543^***^ | -36.6722^***^ | -36.7003^***^ |
|  | (0.5406) | (0.5406) | (0.5406) | (0.3022) | (0.3023) | (0.3022) |
| Controls | Yes | Yes | Yes | Yes | Yes | Yes |
| Month | Yes | Yes | Yes | Yes | Yes | Yes |
| City | Yes | Yes | Yes | Yes | Yes | Yes |
| N | 673006 | 673006 | 673006 | 1153089 | 1153089 | 1153089 |
| R^2^ | 0.9691 | 0.9691 | 0.9691 | 0.8251 | 0.8251 | 0.8251 |

**Supplementary Table 22.** Dependent variable: lnPM10

|  | (1)COVID-break | | | (2)Non-break | | |
| --- | --- | --- | --- | --- | --- | --- |
|  | lnPM_10_ | lnPM_10_ | lnPM_10_ | lnPM_10_ | lnPM_10_ | lnPM_10_ |
| BEVtrips | -0.0002^***^ |  |  | 0.0005^***^ |  |  |
|  | (0.0000) |  |  | (0.0001) |  |  |
| lnmileage |  | -0.0002^***^ |  |  | 0.0007^***^ |  |
|  |  | (0.0000) |  |  | (0.0001) |  |
| Avemile |  |  | -0.0002^***^ |  |  | 0.0010^***^ |
|  |  |  | (0.0001) |  |  | (0.0002) |
| cons | -95.9893^***^ | -95.9889^***^ | -95.9831^***^ | -57.8471^***^ | -57.8371^***^ | -57.8505^***^ |
|  | (0.1792) | (0.1791) | (0.1791) | (0.2202) | (0.2202) | (0.2203) |
| Controls | Yes | Yes | Yes | Yes | Yes | Yes |
| Month | Yes | Yes | Yes | Yes | Yes | Yes |
| City | Yes | Yes | Yes | Yes | Yes | Yes |
| N | 673006 | 673006 | 673006 | 1153089 | 1153089 | 1153089 |
| R^2^ | 0.9923 | 0.9923 | 0.9923 | 0.8748 | 0.8748 | 0.8748 |

**Supplementary Table 23.** Dependent variable: lnCO

|  | (1)COVID-break | | | (2)Non-break | | |
| --- | --- | --- | --- | --- | --- | --- |
|  | lnCO | lnCO | lnCO | lnCO | lnCO | lnCO |
| BEVtrips | 0.0001^***^ |  |  | 0.0007^***^ |  |  |
|  | (0.0000) |  |  | (0.0001) |  |  |
| lnmileage |  | -0.0000 |  |  | 0.0005^***^ |  |
|  |  | (0.0000) |  |  | (0.0001) |  |
| Avemile |  |  | -0.0002^***^ |  |  | 0.0001 |
|  |  |  | (0.0000) |  |  | (0.0001) |
| cons | 86.4228^***^ | 86.4201^***^ | 86.4213^***^ | -4.4976^***^ | -4.4977^***^ | -4.5112^***^ |
|  | (0.0680) | (0.0680) | (0.0680) | (0.1337) | (0.1338) | (0.1338) |
| Controls | Yes | Yes | Yes | Yes | Yes | Yes |
| Month | Yes | Yes | Yes | Yes | Yes | Yes |
| City | Yes | Yes | Yes | Yes | Yes | Yes |
| N | 673006 | 673006 | 673006 | 1153089 | 1153089 | 1153089 |
| R^2^ | 0.9983 | 0.9983 | 0.9983 | 0.8961 | 0.8961 | 0.8961 |

**Supplementary Table 24.** Dependent variable: lnSO2

|  | (1)COVID-break | | | (2)Non-break | | |
| --- | --- | --- | --- | --- | --- | --- |
|  | lnSO_2_ | lnSO_2_ | lnSO_2_ | lnSO_2_ | lnSO_2_ | lnSO_2_ |
| BEVtrips | -0.0002^***^ |  |  | -0.0012^***^ |  |  |
|  | (0.0000) |  |  | (0.0001) |  |  |
| lnmileage |  | -0.0001^***^ |  |  | -0.0014^***^ |  |
|  |  | (0.0000) |  |  | (0.0001) |  |
| Avemile |  |  | 0.0000 |  |  | -0.0016^***^ |
|  |  |  | (0.0000) |  |  | (0.0001) |
| cons | 24.9646^***^ | 24.9682^***^ | 24.9708^***^ | -39.7224^***^ | -39.7371^***^ | -39.7084^***^ |
|  | (0.1073) | (0.1073) | (0.1073) | (0.1378) | (0.1378) | (0.1376) |
| Controls | Yes | Yes | Yes | Yes | Yes | Yes |
| Month | Yes | Yes | Yes | Yes | Yes | Yes |
| City | Yes | Yes | Yes | Yes | Yes | Yes |
| N | 673006 | 673006 | 673006 | 1153089 | 1153089 | 1153089 |
| R^2^ | 0.9966 | 0.9966 | 0.9966 | 0.9223 | 0.9224 | 0.9223 |

**Supplementary Table 25.** Dependent variable: lnNO2

|  | (1)COVID-break | | | (2)Non-break | | |
| --- | --- | --- | --- | --- | --- | --- |
|  | lnNO_2_ | lnNO_2_ | lnNO_2_ | lnNO_2_ | lnNO_2_ | lnNO_2_ |
| BEVtrips | 0.0006^***^ |  |  | -0.0017^***^ |  |  |
|  | (0.0000) |  |  | (0.0001) |  |  |
| lnmileage |  | 0.0001^***^ |  |  | -0.0006^***^ |  |
|  |  | (0.0000) |  |  | (0.0001) |  |
| Avemile |  |  | -0.0007^***^ |  |  | 0.0014^***^ |
|  |  |  | (0.0001) |  |  | (0.0001) |
| cons | -1.0e+02^***^ | -1.0e+02^***^ | -1.0e+02^***^ | 20.5359^***^ | 20.5539^***^ | 20.5817^***^ |
|  | (0.3248) | (0.3249) | (0.3248) | (0.1558) | (0.1561) | (0.1561) |
| Controls | Yes | Yes | Yes | Yes | Yes | Yes |
| Month | Yes | Yes | Yes | Yes | Yes | Yes |
| City | Yes | Yes | Yes | Yes | Yes | Yes |
| N | 673006 | 673006 | 673006 | 1153089 | 1153089 | 1153089 |
| R^2^ | 0.9543 | 0.9543 | 0.9543 | 0.9055 | 0.9055 | 0.9055 |

**Supplementary Table 26.** Dependent variable: lnO3

|  | (1)COVID-break | | | (2)Non-break | | |
| --- | --- | --- | --- | --- | --- | --- |
|  | lnO_3_ | lnO_3_ | lnO_3_ | lnO_3_ | lnO_3_ | lnO_3_ |
| BEVtrips | 0.0004^***^ |  |  | 0.0049^***^ |  |  |
|  | (0.0000) |  |  | (0.0002) |  |  |
| lnmileage |  | 0.0000 |  |  | 0.0032^***^ |  |
|  |  | (0.0000) |  |  | (0.0002) |  |
| Avemile |  |  | -0.0006^***^ |  |  | -0.0005^*^ |
|  |  |  | (0.0000) |  |  | (0.0003) |
| cons | 76.0995^***^ | 76.0883^***^ | 76.0910^***^ | -1.3e+02^***^ | -1.3e+02^***^ | -1.3e+02^***^ |
|  | (0.2440) | (0.2441) | (0.2440) | (0.4243) | (0.4249) | (0.4252) |
| Controls | Yes | Yes | Yes | Yes | Yes | Yes |
| Month | Yes | Yes | Yes | Yes | Yes | Yes |
| City | Yes | Yes | Yes | Yes | Yes | Yes |
| N | 673006 | 673006 | 673006 | 1153089 | 1153089 | 1153089 |
| R^2^ | 0.9910 | 0.9910 | 0.9910 | 0.8861 | 0.8861 | 0.8860 |

**Supplementary Table 27.** Independent variable：BEVtrips

|  | (1) | (2) | (3) | (4) | (5) | (6) | (7) | (8) |
| --- | --- | --- | --- | --- | --- | --- | --- | --- |
|  | lnAQI | lnAQI | lnPM_2.5_ | lnPM_10_ | lnCO | lnSO_2_ | lnNO_2_ | lnO_3_ |
| BEVtrips | -0.0001 | 0.0024^***^ | 0.0044^***^ | 0.0024^***^ | 0.0010^***^ | -0.0005^***^ | 0.0039^***^ | -0.0055^***^ |
|  | (0.0002) | (0.0001) | (0.0001) | (0.0001) | (0.0001) | (0.0000) | (0.0001) | (0.0002) |
| cons | 4.3249^***^ | -14.7148^***^ | 6.3638^***^ | -32.9454^***^ | 13.2996^***^ | -5.3798^***^ | 15.0275^***^ | -1.1e+02^***^ |
|  | (0.0012) | (0.2633) | (0.3399) | (0.2359) | (0.1041) | (0.0853) | (0.1522) | (0.2529) |
| Controls | No | Yes | Yes | Yes | Yes | Yes | Yes | Yes |
| Month | Yes | Yes | Yes | Yes | Yes | Yes | Yes | Yes |
| City | Yes | Yes | Yes | Yes | Yes | Yes | Yes | Yes |
| N | 1548041 | 1548041 | 1548041 | 1548041 | 1548041 | 1548041 | 1548041 | 1548041 |
| R^2^ | 0.5214 | 0.8486 | 0.8459 | 0.8872 | 0.9095 | 0.9592 | 0.8997 | 0.8689 |

**Supplementary Table 28.** Independent variable：lnmileage

|  | (1) | (2) | (3) | (4) | (5) | (6) | (7) | (8) |
| --- | --- | --- | --- | --- | --- | --- | --- | --- |
|  | lnAQI | lnAQI | lnPM_2.5_ | lnPM_10_ | lnCO | lnSO_2_ | lnNO_2_ | lnO_3_ |
| lnmileage | 0.0024^***^ | 0.0004^***^ | 0.0012^***^ | 0.0005^***^ | 0.0005^***^ | -0.0004^***^ | 0.0014^***^ | -0.0017^***^ |
|  | (0.0002) | (0.0001) | (0.0001) | (0.0001) | (0.0000) | (0.0000) | (0.0001) | (0.0002) |
| cons | 4.3117^***^ | -14.7843^***^ | 6.2521^***^ | -33.0113^***^ | 13.2820^***^ | -5.3782^***^ | 14.9392^***^ | -1.1e+02^***^ |
|  | (0.0014) | (0.2631) | (0.3396) | (0.2358) | (0.1042) | (0.0854) | (0.1519) | (0.2526) |
| Controls | No | Yes | Yes | Yes | Yes | Yes | Yes | Yes |
| Month | Yes | Yes | Yes | Yes | Yes | Yes | Yes | Yes |
| City | Yes | Yes | Yes | Yes | Yes | Yes | Yes | Yes |
| N | 1548041 | 1548041 | 1548041 | 1548041 | 1548041 | 1548041 | 1548041 | 1548041 |
| R^2^ | 0.5214 | 0.8486 | 0.8457 | 0.8871 | 0.9095 | 0.9592 | 0.8996 | 0.8689 |

**Supplementary Table 29.** Independent variable：Avemile

|  | (1) | (2) | (3) | (4) | (5) | (6) | (7) | (8) |
| --- | --- | --- | --- | --- | --- | --- | --- | --- |
|  | lnAQI | lnAQI | lnPM_2.5_ | lnPM_10_ | lnCO | lnSO_2_ | lnNO_2_ | lnO_3_ |
| Avemile | 0.0060^***^ | -0.0031^***^ | -0.0047^***^ | -0.0029^***^ | -0.0006^***^ | -0.0002^***^ | -0.0035^***^ | 0.0054^***^ |
|  | (0.0003) | (0.0002) | (0.0002) | (0.0002) | (0.0001) | (0.0001) | (0.0001) | (0.0003) |
| cons | 4.3067^***^ | -14.8069^***^ | 6.1984^***^ | -33.0368^***^ | 13.2635^***^ | -5.3621^***^ | 14.8817^***^ | -1.1e+02^***^ |
|  | (0.0013) | (0.2630) | (0.3396) | (0.2358) | (0.1041) | (0.0854) | (0.1520) | (0.2532) |
| Controls | No | Yes | Yes | Yes | Yes | Yes | Yes | Yes |
| Month | Yes | Yes | Yes | Yes | Yes | Yes | Yes | Yes |
| City | Yes | Yes | Yes | Yes | Yes | Yes | Yes | Yes |
| N | 1548041 | 1548041 | 1548041 | 1548041 | 1548041 | 1548041 | 1548041 | 1548041 |
| R^2^ | 0.5216 | 0.8486 | 0.8458 | 0.8872 | 0.9095 | 0.9592 | 0.8996 | 0.8689 |

**Supplementary Information 8.2: Season-specific effectiveness of BEVs in reducing emissions**

To address the issue of season-specific effectiveness of Battery Electric Vehicles (BEVs) in reducing emissions, we conducted a focused sub-sample regression analysis using our 2019 data, segmented by season. The decision to exclusively use 2019 data was twofold: firstly, our study period (January 2019 to October 2020) does not encompass the complete four seasons of 2020, and including 2020 data might result in an uneven sample distribution, potentially skewing our conclusions. Secondly, the outbreak of COVID-19 in 2020 introduced significant extraneous variables that could obscure the net effect of seasonal factors on our study.

Consequently, our analysis concentrated on the differential impact of EV trips on air quality across the four seasons of 2019. It’s important to note that due to the presence of numerous seasonal or monthly variables, which could lead to multicollinearity issues, we chose to exclude these control variables, focusing only on time and area fixed effects. The results, detailed in Supplementary Table 30-36, demonstrate a seasonal variation in the air quality improvement attributable to EV travel. Particularly in winter, the regression coefficients for most air quality indicators, except lnSO2, are predominantly positive, indicating a reduced environmental benefit from BEVs during this season compared to others.

Furthermore, our literature review, including studies such as Yang et al. (2018), supports the notion of seasonal variability in BEV environmental benefits. These studies suggest that BEV mileage and efficiency are heavily dependent on ambient temperatures. In winter, the use of heating systems significantly alters battery cycle efficiency, exacerbating BEV degradation and leading to increased greenhouse gas emissions in colder temperatures.

By isolating and analyzing seasonal data, our study aims to provide a nuanced understanding of the environmental performance of BEVs, acknowledging that their benefits can vary with seasonal conditions.

**Supplementary Table 30.** Dependent variable: lnAQI

|  | (1)Spring | | | (2)Summer | | | (3)Autumn | | | (4)Winter | | |
| --- | --- | --- | --- | --- | --- | --- | --- | --- | --- | --- | --- | --- |
|  | lnAQI | lnAQI | lnAQI | lnAQI | lnAQI | lnAQI | lnAQI | lnAQI | lnAQI | lnAQI | lnAQI | lnAQI |
| BEVtrips | 0.0011^***^ |  |  | 0.0116^***^ |  |  | 0.0001 |  |  | 0.0065^***^ |  |  |
|  | (0.0002) |  |  | (0.0008) |  |  | (0.0002) |  |  | (0.0008) |  |  |
| lnmileage |  | 0.0002 |  |  | 0.0017^**^ |  |  | 0.0007^***^ |  |  | 0.0068^***^ |  |
|  |  | (0.0002) |  |  | (0.0007) |  |  | (0.0001) |  |  | (0.0007) |  |
| Avemile |  |  | -0.0021^***^ |  |  | -0.0138^***^ |  |  | 0.0019^***^ |  |  | 0.0064^***^ |
|  |  |  | (0.0004) |  |  | (0.0011) |  |  | (0.0002) |  |  | (0.0011) |
| cons | 4.2189^***^ | 4.2206^***^ | 4.2281^***^ | 3.9632^***^ | 3.9869^***^ | 4.0398^***^ | 4.0184^***^ | 4.0147^***^ | 4.0127^***^ | 4.2714^***^ | 4.2507^***^ | 4.2675^***^ |
|  | (0.0006) | (0.0011) | (0.0012) | (0.0023) | (0.0042) | (0.0035) | (0.0005) | (0.0008) | (0.0007) | (0.0019) | (0.0036) | (0.0034) |
| Month | Yes | Yes | Yes | Yes | Yes | Yes | Yes | Yes | Yes | Yes | Yes | Yes |
| City | Yes | Yes | Yes | Yes | Yes | Yes | Yes | Yes | Yes | Yes | Yes | Yes |
| N | 126704 | 126704 | 126704 | 186924 | 186924 | 186924 | 261319 | 261319 | 261319 | 169961 | 169961 | 169961 |
| R^2^ | 0.7544 | 0.7543 | 0.7544 | 0.4672 | 0.4659 | 0.4671 | 0.8445 | 0.8445 | 0.8445 | 0.2289 | 0.2295 | 0.2286 |

**Supplementary Table 31.** Dependent variable: lnPM_2.5_

|  | (1)Spring | | | (2)Summer | | | (3)Autumn | | | (4)Winter | | |
| --- | --- | --- | --- | --- | --- | --- | --- | --- | --- | --- | --- | --- |
|  | lnPM_2.5_ | lnPM_2.5_ | lnPM_2.5_ | lnPM_2.5_ | lnPM_2.5_ | lnPM_2.5_ | lnPM_2.5_ | lnPM_2.5_ | lnPM_2.5_ | lnPM_2.5_ | lnPM_2.5_ | lnPM_2.5_ |
| BEVtrips | 0.0001 |  |  | 0.0146^***^ |  |  | -0.0010^***^ |  |  | 0.0124^***^ |  |  |
|  | (0.0001) |  |  | (0.0009) |  |  | (0.0002) |  |  | (0.0010) |  |  |
| lnmileage |  | -0.0004^***^ |  |  | 0.0026^***^ |  |  | -0.0006^***^ |  |  | 0.0108^***^ |  |
|  |  | (0.0001) |  |  | (0.0008) |  |  | (0.0002) |  |  | (0.0008) |  |
| Avemile |  |  | -0.0018^***^ |  |  | -0.0162^***^ |  |  | 0.0001 |  |  | 0.0073^***^ |
|  |  |  | (0.0002) |  |  | (0.0014) |  |  | (0.0003) |  |  | (0.0013) |
| cons | 3.7652^***^ | 3.7679^***^ | 3.7712^***^ | 3.3274^***^ | 3.3541^***^ | 3.4202^***^ | 3.4224^***^ | 3.4234^***^ | 3.4195^***^ | 3.8515^***^ | 3.8231^***^ | 3.8582^***^ |
|  | (0.0003) | (0.0006) | (0.0007) | (0.0027) | (0.0050) | (0.0042) | (0.0006) | (0.0009) | (0.0008) | (0.0022) | (0.0042) | (0.0039) |
| Month | Yes | Yes | Yes | Yes | Yes | Yes | Yes | Yes | Yes | Yes | Yes | Yes |
| City | Yes | Yes | Yes | Yes | Yes | Yes | Yes | Yes | Yes | Yes | Yes | Yes |
| N | 126704 | 126704 | 126704 | 186924 | 186924 | 186924 | 261319 | 261319 | 261319 | 169961 | 169961 | 169961 |
| R^2^ | 0.9766 | 0.9766 | 0.9766 | 0.4696 | 0.4682 | 0.4693 | 0.8698 | 0.8698 | 0.8698 | 0.1957 | 0.1963 | 0.1939 |

**Supplementary Table 32.** Dependent variable: lnPM_10_

|  | (1)Spring | | | (2)Summer | | | (3)Autumn | | | (4)Winter | | |
| --- | --- | --- | --- | --- | --- | --- | --- | --- | --- | --- | --- | --- |
|  | lnPM_10_ | lnPM_10_ | lnPM_10_ | lnPM_10_ | lnPM_10_ | lnPM_10_ | lnPM_10_ | lnPM_10_ | lnPM_10_ | lnPM_10_ | lnPM_10_ | lnPM_10_ |
| BEVtrips | 0.0152^***^ |  |  | 0.0118^***^ |  |  | -0.0021^***^ |  |  | 0.0016 |  |  |
|  | (0.0005) |  |  | (0.0009) |  |  | (0.0002) |  |  | (0.0011) |  |  |
| lnmileage |  | 0.0109^***^ |  |  | 0.0016^*^ |  |  | 0.0002 |  |  | 0.0048^***^ |  |
|  |  | (0.0005) |  |  | (0.0008) |  |  | (0.0002) |  |  | (0.0009) |  |
| Avemile |  |  | 0.0007 |  |  | -0.0142^***^ |  |  | 0.0051^***^ |  |  | 0.0088^***^ |
|  |  |  | (0.0009) |  |  | (0.0013) |  |  | (0.0003) |  |  | (0.0015) |
| cons | 4.1070^***^ | 4.0829^***^ | 4.1407^***^ | 3.8259^***^ | 3.8505^***^ | 3.9043^***^ | 3.9436^***^ | 3.9366^***^ | 3.9217^***^ | 4.3015^***^ | 4.2800^***^ | 4.2791^***^ |
|  | (0.0014) | (0.0027) | (0.0030) | (0.0028) | (0.0049) | (0.0042) | (0.0007) | (0.0011) | (0.0011) | (0.0024) | (0.0047) | (0.0046) |
| Month | Yes | Yes | Yes | Yes | Yes | Yes | Yes | Yes | Yes | Yes | Yes | Yes |
| City | Yes | Yes | Yes | Yes | Yes | Yes | Yes | Yes | Yes | Yes | Yes | Yes |
| N | 126704 | 126704 | 126704 | 186924 | 186924 | 186924 | 261319 | 261319 | 261319 | 169961 | 169961 | 169961 |
| R^2^ | 0.3666 | 0.3649 | 0.3602 | 0.4452 | 0.4443 | 0.4451 | 0.9043 | 0.9042 | 0.9044 | 0.2882 | 0.2885 | 0.2887 |

**Supplementary Table 33.** Dependent variable: lnCO

|  | (1)Spring | | | (2)Summer | | | (3)Autumn | | | (4)Winter | | |
| --- | --- | --- | --- | --- | --- | --- | --- | --- | --- | --- | --- | --- |
|  | lnCO | lnCO | lnCO | lnCO | lnCO | lnCO | lnCO | lnCO | lnCO | lnCO | lnCO | lnCO |
| BEVtrips | -0.0056^***^ |  |  | 0.0032^***^ |  |  | 0.0063^***^ |  |  | 0.0049^***^ |  |  |
|  | (0.0001) |  |  | (0.0003) |  |  | (0.0002) |  |  | (0.0002) |  |  |
| lnmileage |  | -0.0043^***^ |  |  | 0.0002 |  |  | 0.0023^***^ |  |  | 0.0024^***^ |  |
|  |  | (0.0001) |  |  | (0.0003) |  |  | (0.0002) |  |  | (0.0002) |  |
| Avemile |  |  | -0.0011^***^ |  |  | -0.0045^***^ |  |  | -0.0061^***^ |  |  | -0.0014^***^ |
|  |  |  | (0.0002) |  |  | (0.0004) |  |  | (0.0004) |  |  | (0.0002) |
| cons | -0.4447^***^ | -0.4345^***^ | -0.4547^***^ | -0.4574^***^ | -0.4491^***^ | -0.4341^***^ | -0.5017^***^ | -0.4979^***^ | -0.4650^***^ | -0.1034^***^ | -0.1050^***^ | -0.0884^***^ |
|  | (0.0003) | (0.0007) | (0.0008) | (0.0010) | (0.0017) | (0.0013) | (0.0006) | (0.0011) | (0.0012) | (0.0006) | (0.0009) | (0.0008) |
| Month | Yes | Yes | Yes | Yes | Yes | Yes | Yes | Yes | Yes | Yes | Yes | Yes |
| City | Yes | Yes | Yes | Yes | Yes | Yes | Yes | Yes | Yes | Yes | Yes | Yes |
| N | 126704 | 126704 | 126704 | 186924 | 186924 | 186924 | 261319 | 261319 | 261319 | 169961 | 169961 | 169961 |
| R^2^ | 0.9053 | 0.9049 | 0.9032 | 0.5426 | 0.5422 | 0.5428 | 0.7350 | 0.7338 | 0.7341 | 0.8655 | 0.8645 | 0.8640 |

**Supplementary Table 34.** Dependent variable: lnSO_2_

|  | (1)Spring | | | (2)Summer | | | (3)Autumn | | | (4)Winter | | |
| --- | --- | --- | --- | --- | --- | --- | --- | --- | --- | --- | --- | --- |
|  | lnSO_2_ | lnSO_2_ | lnSO_2_ | lnSO_2_ | lnSO_2_ | lnSO_2_ | lnSO_2_ | lnSO_2_ | lnSO_2_ | lnSO_2_ | lnSO_2_ | lnSO_2_ |
| BEVtrips | -0.0147^***^ |  |  | 0.0036^***^ |  |  | 0.0046^***^ |  |  | -0.0250^***^ |  |  |
|  | (0.0003) |  |  | (0.0005) |  |  | (0.0003) |  |  | (0.0012) |  |  |
| lnmileage |  | -0.0110^***^ |  |  | 0.0005 |  |  | 0.0007^***^ |  |  | -0.0153^***^ |  |
|  |  | (0.0003) |  |  | (0.0005) |  |  | (0.0003) |  |  | (0.0009) |  |
| Avemile |  |  | -0.0021^***^ |  |  | -0.0043^***^ |  |  | -0.0077^***^ |  |  | 0.0003 |
|  |  |  | (0.0006) |  |  | (0.0008) |  |  | (0.0004) |  |  | (0.0015) |
| cons | 1.6974^***^ | 1.7230^***^ | 1.6692^***^ | 1.4449^***^ | 1.4522^***^ | 1.4686^***^ | 1.2527^***^ | 1.2615^***^ | 1.2897^***^ | 2.1920^***^ | 2.2153^***^ | 2.1345^***^ |
|  | (0.0007) | (0.0015) | (0.0019) | (0.0016) | (0.0029) | (0.0024) | (0.0008) | (0.0015) | (0.0014) | (0.0031) | (0.0050) | (0.0048) |
| Month | Yes | Yes | Yes | Yes | Yes | Yes | Yes | Yes | Yes | Yes | Yes | Yes |
| City | Yes | Yes | Yes | Yes | Yes | Yes | Yes | Yes | Yes | Yes | Yes | Yes |
| N | 126704 | 126704 | 126704 | 186924 | 186924 | 186924 | 261319 | 261319 | 261319 | 169961 | 169961 | 169961 |
| R^2^ | 0.6839 | 0.6820 | 0.6747 | 0.7641 | 0.7640 | 0.7640 | 0.8674 | 0.8672 | 0.8674 | 0.6278 | 0.6262 | 0.6238 |

**Supplementary Table 35.** Dependent variable: lnNO_2_

|  | (1)Spring | | | (2)Summer | | | (3)Autumn | | | (4)Winter | | |
| --- | --- | --- | --- | --- | --- | --- | --- | --- | --- | --- | --- | --- |
|  | lnNO_2_ | lnNO_2_ | lnNO_2_ | lnNO_2_ | lnNO_2_ | lnNO_2_ | lnNO_2_ | lnNO_2_ | lnNO_2_ | lnNO_2_ | lnNO_2_ | lnNO_2_ |
| BEVtrips | 0.0065^***^ |  |  | 0.0012^***^ |  |  | -0.0074^***^ |  |  | 0.0022^***^ |  |  |
|  | (0.0002) |  |  | (0.0001) |  |  | (0.0002) |  |  | (0.0006) |  |  |
| lnmileage |  | 0.0048^***^ |  |  | -0.0000 |  |  | -0.0051^***^ |  |  | 0.0028^***^ |  |
|  |  | (0.0001) |  |  | (0.0001) |  |  | (0.0002) |  |  | (0.0005) |  |
| Avemile |  |  | 0.0007^**^ |  |  | -0.0020^***^ |  |  | -0.0005 |  |  | 0.0035^***^ |
|  |  |  | (0.0003) |  |  | (0.0002) |  |  | (0.0004) |  |  | (0.0008) |
| cons | 3.6036^***^ | 3.5926^***^ | 3.6169^***^ | 3.2461^***^ | 3.2499^***^ | 3.2559^***^ | 3.4528^***^ | 3.4625^***^ | 3.4336^***^ | 3.8743^***^ | 3.8643^***^ | 3.8689^***^ |
|  | (0.0004) | (0.0007) | (0.0009) | (0.0004) | (0.0008) | (0.0006) | (0.0006) | (0.0011) | (0.0013) | (0.0013) | (0.0025) | (0.0024) |
| Month | Yes | Yes | Yes | Yes | Yes | Yes | Yes | Yes | Yes | Yes | Yes | Yes |
| City | Yes | Yes | Yes | Yes | Yes | Yes | Yes | Yes | Yes | Yes | Yes | Yes |
| N | 126704 | 126704 | 126704 | 186924 | 186924 | 186924 | 261319 | 261319 | 261319 | 169961 | 169961 | 169961 |
| R^2^ | 0.8597 | 0.8590 | 0.8568 | 0.5676 | 0.5673 | 0.5678 | 0.9040 | 0.9037 | 0.9032 | 0.8375 | 0.8376 | 0.8376 |

**Supplementary Table 36.** Dependent variable: lnO_3_

|  | (1)Spring | | | (2)Summer | | | (3)Autumn | | | (4)Winter | | |
| --- | --- | --- | --- | --- | --- | --- | --- | --- | --- | --- | --- | --- |
|  | lnO_3_ | lnO_3_ | lnO_3_ | lnO_3_ | lnO_3_ | lnO_3_ | lnO_3_ | lnO_3_ | lnO_3_ | lnO_3_ | lnO_3_ | lnO_3_ |
| BEVtrips | 0.0118^***^ |  |  | 0.0122^***^ |  |  | 0.0020^**^ |  |  | -0.0026^***^ |  |  |
|  | (0.0003) |  |  | (0.0007) |  |  | (0.0010) |  |  | (0.0006) |  |  |
| lnmileage |  | 0.0091^***^ |  |  | 0.0018^***^ |  |  | 0.0111^***^ |  |  | 0.0005 |  |
|  |  | (0.0002) |  |  | (0.0007) |  |  | (0.0008) |  |  | (0.0005) |  |
| Avemile |  |  | 0.0026^***^ |  |  | -0.0145^***^ |  |  | 0.0312^***^ |  |  | 0.0049^***^ |
|  |  |  | (0.0005) |  |  | (0.0011) |  |  | (0.0018) |  |  | (0.0008) |
| cons | 4.0613^***^ | 4.0394^***^ | 4.0811^***^ | 4.5238^***^ | 4.5486^***^ | 4.6044^***^ | 4.4769^***^ | 4.4169^***^ | 4.3846^***^ | 3.5281^***^ | 3.5194^***^ | 3.5076^***^ |
|  | (0.0006) | (0.0013) | (0.0017) | (0.0022) | (0.0040) | (0.0034) | (0.0029) | (0.0052) | (0.0059) | (0.0013) | (0.0025) | (0.0025) |
| Month | Yes | Yes | Yes | Yes | Yes | Yes | Yes | Yes | Yes | Yes | Yes | Yes |
| City | Yes | Yes | Yes | Yes | Yes | Yes | Yes | Yes | Yes | Yes | Yes | Yes |
| N | 126704 | 126704 | 126704 | 186924 | 186924 | 186924 | 261319 | 261319 | 261319 | 169961 | 169961 | 169961 |
| R^2^ | 0.9142 | 0.9138 | 0.9121 | 0.4475 | 0.4460 | 0.4474 | 0.7470 | 0.7473 | 0.7478 | 0.7668 | 0.7668 | 0.7670 |

**Supplementary Information 8.3: Differential impacts of different cities and different vehicle types**

In order to further explore the differential impacts of different cities and different vehicle types, we divided our study sample into the region overall, Beijing, Shanghai and Shenzhen, and then regressed the sample by vehicle type (private passenger cars, rental passenger cars and internet taxis), and the results are shown in Supplementary Table 37-57, and it's worth noting that there are no electric rental passenger cars in Beijing in our study data. According to the regression results, the environmental benefits of the use of electric private passenger cars are significant for the three cities, and overall Shanghai has the best environmental benefits, followed by Shanghai and finally Shenzhen. The environmental benefits of electric rental cars are not significant, but may instead reduce air quality, a feature seen in both Shanghai and Shenzhen, and particularly evident in Shenzhen. The environmental benefits of electric netbook cars are lower than those of electric private passenger cars, but overall significantly higher than those of electric rental passenger cars, and this is best seen in the three cities with electric netbook cars in Shanghai, followed by Beijing and finally Shanghai. Based on the above findings, we suggest that electric private passenger cars be vigorously promoted in Beijing, electric net-journey cars be vigorously promoted in Shanghai, and that electric rental passenger cars and electric net-journey cars be appropriately cited in Shenzhen due to its predominantly clean energy and high environmental quality.

**Supplementary Table 37.** Heterogeneity analysis: Private BEVs for lnAQI

|  | (1)Three cities | | | (2)Beijing | | | (3)Shanghai | | | (4)Shenzhen | | |
| --- | --- | --- | --- | --- | --- | --- | --- | --- | --- | --- | --- | --- |
|  | lnAQI | lnAQI | lnAQI | lnAQI | lnAQI | lnAQI | lnAQI | lnAQI | lnAQI | lnAQI | lnAQI | lnAQI |
| BEVtrips | -0.0241^***^ |  |  | -0.0287^***^ |  |  | 0.0137^***^ |  |  | -0.0026 |  |  |
|  | (0.0002) |  |  | (0.0002) |  |  | (0.0010) |  |  | (0.0037) |  |  |
| lnmileage |  | -0.0288^***^ |  |  | -0.0308^***^ |  |  | -0.0116^***^ |  |  | -0.0140^***^ |  |
|  |  | (0.0002) |  |  | (0.0002) |  |  | (0.0007) |  |  | (0.0026) |  |
| Avemile |  |  | -0.0296^***^ |  |  | -0.0270^***^ |  |  | -0.0511^***^ |  |  | -0.0269^***^ |
|  |  |  | (0.0003) |  |  | (0.0003) |  |  | (0.0012) |  |  | (0.0038) |
| cons | 4.2027^***^ | 4.2992^***^ | 4.2125^***^ | 4.2610^***^ | 4.3553^***^ | 4.2479^***^ | 3.8919^***^ | 4.0102^***^ | 4.0878^***^ | 3.5654^***^ | 3.6196^***^ | 3.6434^***^ |
|  | (0.0007) | (0.0012) | (0.0009) | (0.0007) | (0.0012) | (0.0009) | (0.0032) | (0.0047) | (0.0035) | (0.0040) | (0.0107) | (0.0114) |
| N | 1369978 | 1369978 | 1369978 | 1172398 | 1172398 | 1172398 | 162560 | 162560 | 162560 | 35020 | 35020 | 35020 |
| R^2^ | 0.0099 | 0.0190 | 0.0074 | 0.0157 | 0.0246 | 0.0067 | 0.0024 | 0.0021 | 0.0181 | 0.0000 | 0.0012 | 0.0021 |

**Supplementary Table 38.** Heterogeneity analysis: Taxi BEVs for lnAQI

|  | (1)Shanghai and Shenzhen | | | (2)Shanghai | | | (3)Shenzhen | | |
| --- | --- | --- | --- | --- | --- | --- | --- | --- | --- |
|  | lnAQI | lnAQI | lnAQI | lnAQI | lnAQI | lnAQI | lnAQI | lnAQI | lnAQI |
| BEVtrips | 0.0314^***^ |  |  | 0.0407^***^ |  |  | 0.0285^***^ |  |  |
|  | (0.0015) |  |  | (0.0014) |  |  | (0.0019) |  |  |
| lnmileage |  | 0.0206^***^ |  |  | 0.0126^***^ |  |  | 0.0219^***^ |  |
|  |  | (0.0010) |  |  | (0.0015) |  |  | (0.0012) |  |
| Avemile |  |  | 0.0077^***^ |  |  | -0.0467^***^ |  |  | 0.0164^***^ |
|  |  |  | (0.0014) |  |  | (0.0018) |  |  | (0.0016) |
| cons | 3.6223^***^ | 3.5536^***^ | 3.6366^***^ | 3.7884^***^ | 3.8434^***^ | 4.1506^***^ | 3.5884^***^ | 3.5059^***^ | 3.5506^***^ |
|  | (0.0022) | (0.0058) | (0.0059) | (0.0056) | (0.0128) | (0.0076) | (0.0019) | (0.0060) | (0.0066) |
| N | 141762 | 141762 | 141762 | 21829 | 21829 | 21829 | 119917 | 119917 | 119917 |
| R^2^ | 0.0046 | 0.0038 | 0.0003 | 0.0326 | 0.0035 | 0.0243 | 0.0031 | 0.0039 | 0.0012 |

**Supplementary Table 39.** Heterogeneity analysis: Ride-hailing BEVs for lnAQI

|  | (1)Three cities | | | (2)Beijing | | | (3)Shanghai | | | (4)Shenzhen | | |
| --- | --- | --- | --- | --- | --- | --- | --- | --- | --- | --- | --- | --- |
|  | lnAQI | lnAQI | lnAQI | lnAQI | lnAQI | lnAQI | lnAQI | lnAQI | lnAQI | lnAQI | lnAQI | lnAQI |
| BEVtrips | 0.0072^***^ |  |  | -0.0070^***^ |  |  | 0.0173^***^ |  |  | 0.0250^***^ |  |  |
|  | (0.0006) |  |  | (0.0006) |  |  | (0.0010) |  |  | (0.0019) |  |  |
| lnmileage |  | -0.0066^***^ |  |  | -0.0132^***^ |  |  | -0.0008 |  |  | -0.0006 |  |
|  |  | (0.0005) |  |  | (0.0005) |  |  | (0.0009) |  |  | (0.0012) |  |
| Avemile |  |  | -0.0275^***^ |  |  | -0.0279^***^ |  |  | -0.0385^***^ |  |  | -0.0237^***^ |
|  |  |  | (0.0009) |  |  | (0.0009) |  |  | (0.0015) |  |  | (0.0016) |
| cons | 3.8724^***^ | 3.9306^***^ | 3.9947^***^ | 4.2145^***^ | 4.2875^***^ | 4.2951^***^ | 3.8857^***^ | 3.9561^***^ | 4.1017^***^ | 3.6013^***^ | 3.6248^***^ | 3.7120^***^ |
|  | (0.0015) | (0.0032) | (0.0033) | (0.0022) | (0.0039) | (0.0035) | (0.0037) | (0.0067) | (0.0060) | (0.0015) | (0.0056) | (0.0059) |
| N | 314355 | 314355 | 314355 | 116283 | 116283 | 116283 | 55353 | 55353 | 55353 | 142719 | 142719 | 142719 |
| R^2^ | 0.0005 | 0.0007 | 0.0046 | 0.0015 | 0.0081 | 0.0111 | 0.0061 | 0.0000 | 0.0146 | 0.0022 | 0.0000 | 0.0022 |

**Supplementary Table 40.** Heterogeneity analysis: Private BEVs for lnPM25

|  | (1)Three cities | | | (2)Beijing | | | (3)Shanghai | | | (4)Shenzhen | | |
| --- | --- | --- | --- | --- | --- | --- | --- | --- | --- | --- | --- | --- |
|  | lnPM_2.5_ | lnPM_2.5_ | lnPM_2.5_ | lnPM_2.5_ | lnPM_2.5_ | lnPM_2.5_ | lnPM_2.5_ | lnPM_2.5_ | lnPM_2.5_ | lnPM_2.5_ | lnPM_2.5_ | lnPM_2.5_ |
| BEVtrips | -0.0388^***^ |  |  | -0.0440^***^ |  |  | 0.0031^**^ |  |  | -0.0072 |  |  |
|  | (0.0003) |  |  | (0.0003) |  |  | (0.0013) |  |  | (0.0050) |  |  |
| lnmileage |  | -0.0480^***^ |  |  | -0.0508^***^ |  |  | -0.0262^***^ |  |  | -0.0223^***^ |  |
|  |  | (0.0003) |  |  | (0.0003) |  |  | (0.0011) |  |  | (0.0035) |  |
| Avemile |  |  | -0.0525^***^ |  |  | -0.0514^***^ |  |  | -0.0648^***^ |  |  | -0.0392^***^ |
|  |  |  | (0.0005) |  |  | (0.0005) |  |  | (0.0017) |  |  | (0.0051) |
| cons | 3.7097^***^ | 3.8756^***^ | 3.7398^***^ | 3.7703^***^ | 3.9363^***^ | 3.7789^***^ | 3.4152^***^ | 3.5896^***^ | 3.6162^***^ | 2.9132^***^ | 2.9963^***^ | 3.0232^***^ |
|  | (0.0011) | (0.0017) | (0.0014) | (0.0011) | (0.0018) | (0.0014) | (0.0045) | (0.0069) | (0.0049) | (0.0054) | (0.0144) | (0.0152) |
| N | 1369978 | 1369978 | 1369978 | 1172398 | 1172398 | 1172398 | 162560 | 162560 | 162560 | 35020 | 35020 | 35020 |
| R^2^ | 0.0134 | 0.0277 | 0.0122 | 0.0194 | 0.0351 | 0.0129 | 0.0001 | 0.0052 | 0.0142 | 0.0001 | 0.0017 | 0.0025 |

**Supplementary Table 41.** Heterogeneity analysis: Taxi BEVs for lnPM25

|  | (1)Shanghai and Shenzhen | | | (2)Shanghai | | | (3)Shenzhen | | |
| --- | --- | --- | --- | --- | --- | --- | --- | --- | --- |
|  | lnPM_2.5_ | lnPM_2.5_ | lnPM_2.5_ | lnPM_2.5_ | lnPM_2.5_ | lnPM_2.5_ | lnPM_2.5_ | lnPM_2.5_ | lnPM_2.5_ |
| BEVtrips | 0.0508^***^ |  |  | 0.0807^***^ |  |  | 0.0412^***^ |  |  |
|  | (0.0020) |  |  | (0.0022) |  |  | (0.0025) |  |  |
| lnmileage |  | 0.0274^***^ |  |  | 0.0125^***^ |  |  | 0.0299^***^ |  |
|  |  | (0.0014) |  |  | (0.0022) |  |  | (0.0016) |  |
| Avemile |  |  | 0.0013 |  |  | -0.1175^***^ |  |  | 0.0203^***^ |
|  |  |  | (0.0020) |  |  | (0.0031) |  |  | (0.0022) |
| cons | 2.9808^***^ | 2.9025^***^ | 3.0500^***^ | 3.1202^***^ | 3.3328^***^ | 3.9478^***^ | 2.9438^***^ | 2.8337^***^ | 2.9032^***^ |
|  | (0.0030) | (0.0078) | (0.0082) | (0.0084) | (0.0183) | (0.0134) | (0.0026) | (0.0081) | (0.0089) |
| N | 141762 | 141762 | 141762 | 21829 | 21829 | 21829 | 119917 | 119917 | 119917 |
| R^2^ | 0.0067 | 0.0037 | 0.0000 | 0.0631 | 0.0017 | 0.0759 | 0.0036 | 0.0040 | 0.0010 |

**Supplementary Table 42.** Heterogeneity analysis: Ride-hailing BEVs for lnPm25

|  | (1)Three cities | | | (2)Beijing | | | (3)Shanghai | | | (4)Shenzhen | | |
| --- | --- | --- | --- | --- | --- | --- | --- | --- | --- | --- | --- | --- |
|  | lnPM_2.5_ | lnPM_2.5_ | lnPM_2.5_ | lnPM_2.5_ | lnPM_2.5_ | lnPM_2.5_ | lnPM_2.5_ | lnPM_2.5_ | lnPM_2.5_ | lnPM_2.5_ | lnPM_2.5_ | lnPM_2.5_ |
| BEVtrips | 0.0067^***^ |  |  | -0.0147^***^ |  |  | 0.0245^***^ |  |  | 0.0321^***^ |  |  |
|  | (0.0009) |  |  | (0.0009) |  |  | (0.0015) |  |  | (0.0025) |  |  |
| lnmileage |  | -0.0141^***^ |  |  | -0.0238^***^ |  |  | -0.0099^***^ |  |  | -0.0030^*^ |  |
|  |  | (0.0007) |  |  | (0.0008) |  |  | (0.0013) |  |  | (0.0016) |  |
| Avemile |  |  | -0.0453^***^ |  |  | -0.0457^***^ |  |  | -0.0786^***^ |  |  | -0.0343^***^ |
|  |  |  | (0.0012) |  |  | (0.0014) |  |  | (0.0024) |  |  | (0.0021) |
| cons | 3.2981^***^ | 3.4013^***^ | 3.4872^***^ | 3.7094^***^ | 3.8330^***^ | 3.8287^***^ | 3.3540^***^ | 3.5199^***^ | 3.7544^***^ | 2.9589^***^ | 2.9991^***^ | 3.1158^***^ |
|  | (0.0021) | (0.0044) | (0.0045) | (0.0033) | (0.0059) | (0.0054) | (0.0054) | (0.0098) | (0.0094) | (0.0021) | (0.0074) | (0.0079) |
| N | 314355 | 314355 | 314355 | 116283 | 116283 | 116283 | 55353 | 55353 | 55353 | 142719 | 142719 | 142719 |
| R^2^ | 0.0002 | 0.0016 | 0.0068 | 0.0034 | 0.0133 | 0.0151 | 0.0063 | 0.0014 | 0.0308 | 0.0020 | 0.0000 | 0.0026 |

**Supplementary Table 43.** Heterogeneity analysis: Private BEVs for lnPM10

|  | (1)Three cities | | | (2)Beijing | | | (3)Shanghai | | | (4)Shenzhen | | |
| --- | --- | --- | --- | --- | --- | --- | --- | --- | --- | --- | --- | --- |
|  | lnPM_10_ | lnPM_10_ | lnPM_10_ | lnPM_10_ | lnPM_10_ | lnPM_10_ | lnPM_10_ | lnPM_10_ | lnPM_10_ | lnPM_10_ | lnPM_10_ | lnPM_10_ |
| BEVtrips | -0.0096^***^ |  |  | -0.0139^***^ |  |  | 0.0280^***^ |  |  | -0.0034 |  |  |
|  | (0.0003) |  |  | (0.0003) |  |  | (0.0010) |  |  | (0.0044) |  |  |
| lnmileage |  | -0.0207^***^ |  |  | -0.0225^***^ |  |  | -0.0045^***^ |  |  | -0.0160^***^ |  |
|  |  | (0.0002) |  |  | (0.0002) |  |  | (0.0008) |  |  | (0.0032) |  |
| Avemile |  |  | -0.0370^***^ |  |  | -0.0342^***^ |  |  | -0.0608^***^ |  |  | -0.0304^***^ |
|  |  |  | (0.0004) |  |  | (0.0004) |  |  | (0.0015) |  |  | (0.0045) |
| cons | 4.0458^***^ | 4.1395^***^ | 4.1217^***^ | 4.1125^***^ | 4.2034^***^ | 4.1663^***^ | 3.6434^***^ | 3.7643^***^ | 3.9154^***^ | 3.5397^***^ | 3.6015^***^ | 3.6275^***^ |
|  | (0.0009) | (0.0014) | (0.0012) | (0.0009) | (0.0014) | (0.0012) | (0.0034) | (0.0048) | (0.0043) | (0.0048) | (0.0129) | (0.0135) |
| N | 1369978 | 1369978 | 1369978 | 1172398 | 1172398 | 1172398 | 162560 | 162560 | 162560 | 35020 | 35020 | 35020 |
| R^2^ | 0.0010 | 0.0060 | 0.0071 | 0.0021 | 0.0075 | 0.0062 | 0.0086 | 0.0003 | 0.0225 | 0.0000 | 0.0011 | 0.0019 |

**Supplementary Table 44.** Heterogeneity analysis: Taxi BEVs for lnPM10

|  | (1)Shanghai and Shenzhen | | | (2)Shanghai | | | (3)Shenzhen | | |
| --- | --- | --- | --- | --- | --- | --- | --- | --- | --- |
|  | lnPM_10_ | lnPM_10_ | lnPM_10_ | lnPM_10_ | lnPM_10_ | lnPM_10_ | lnPM_10_ | lnPM_10_ | lnPM_10_ |
| BEVtrips | 0.0215^***^ |  |  | -0.0047^**^ |  |  | 0.0299^***^ |  |  |
|  | (0.0018) |  |  | (0.0021) |  |  | (0.0023) |  |  |
| lnmileage |  | 0.0238^***^ |  |  | 0.0142^***^ |  |  | 0.0254^***^ |  |
|  |  | (0.0012) |  |  | (0.0018) |  |  | (0.0014) |  |
| Avemile |  |  | 0.0238^***^ |  |  | 0.0365^***^ |  |  | 0.0217^***^ |
|  |  |  | (0.0017) |  |  | (0.0025) |  |  | (0.0020) |
| cons | 3.5879^***^ | 3.4868^***^ | 3.5217^***^ | 3.7765^***^ | 3.6407^***^ | 3.5993^***^ | 3.5636^***^ | 3.4649^***^ | 3.5056^***^ |
|  | (0.0026) | (0.0069) | (0.0072) | (0.0081) | (0.0151) | (0.0107) | (0.0023) | (0.0072) | (0.0081) |
| N | 141762 | 141762 | 141762 | 21829 | 21829 | 21829 | 119917 | 119917 | 119917 |
| R^2^ | 0.0015 | 0.0035 | 0.0018 | 0.0004 | 0.0037 | 0.0121 | 0.0023 | 0.0036 | 0.0014 |

**Supplementary Table 45.** Heterogeneity analysis: Ride-hailing BEVs for lnPM10

|  | (1)Three cities | | | (2)Beijing | | | (3)Shanghai | | | (4)Shenzhen | | |
| --- | --- | --- | --- | --- | --- | --- | --- | --- | --- | --- | --- | --- |
|  | lnPM_10_ | lnPM_10_ | lnPM_10_ | lnPM_10_ | lnPM_10_ | lnPM_10_ | lnPM_10_ | lnPM_10_ | lnPM_10_ | lnPM_10_ | lnPM_10_ | lnPM_10_ |
| BEVtrips | 0.0171^***^ |  |  | 0.0139^***^ |  |  | 0.0056^***^ |  |  | 0.0315^***^ |  |  |
|  | (0.0008) |  |  | (0.0008) |  |  | (0.0012) |  |  | (0.0022) |  |  |
| lnmileage |  | 0.0011^*^ |  |  | -0.0006 |  |  | 0.0069^***^ |  |  | 0.0005 |  |
|  |  | (0.0006) |  |  | (0.0006) |  |  | (0.0010) |  |  | (0.0015) |  |
| Avemile |  |  | -0.0237^***^ |  |  | -0.0318^***^ |  |  | 0.0074^***^ |  |  | -0.0276^***^ |
|  |  |  | (0.0010) |  |  | (0.0012) |  |  | (0.0016) |  |  | (0.0019) |
| cons | 3.7736^***^ | 3.8076^***^ | 3.9049^***^ | 4.0468^***^ | 4.1019^***^ | 4.2184^***^ | 3.7382^***^ | 3.7063^***^ | 3.7294^***^ | 3.5790^***^ | 3.6029^***^ | 3.7098^***^ |
|  | (0.0019) | (0.0038) | (0.0040) | (0.0029) | (0.0048) | (0.0047) | (0.0044) | (0.0076) | (0.0061) | (0.0019) | (0.0067) | (0.0071) |
| N | 314355 | 314355 | 314355 | 116283 | 116283 | 116283 | 55353 | 55353 | 55353 | 142719 | 142719 | 142719 |
| R^2^ | 0.0018 | 0.0000 | 0.0023 | 0.0031 | 0.0000 | 0.0076 | 0.0005 | 0.0010 | 0.0004 | 0.0024 | 0.0000 | 0.0020 |

**Supplementary Table 46.** Heterogeneity analysis: Private BEVs for lnCO

|  | (1)Three cities | | | (2)Beijing | | | (3)Shanghai | | | (4)Shenzhen | | |
| --- | --- | --- | --- | --- | --- | --- | --- | --- | --- | --- | --- | --- |
|  | lnCO | lnCO | lnCO | lnCO | lnCO | lnCO | lnCO | lnCO | lnCO | lnCO | lnCO | lnCO |
| BEVtrips | -0.0117^***^ |  |  | -0.0155^***^ |  |  | 0.0211^***^ |  |  | -0.0003 |  |  |
|  | (0.0003) |  |  | (0.0003) |  |  | (0.0006) |  |  | (0.0012) |  |  |
| lnmileage |  | -0.0238^***^ |  |  | -0.0274^***^ |  |  | 0.0066^***^ |  |  | -0.0038^***^ |  |
|  |  | (0.0002) |  |  | (0.0003) |  |  | (0.0005) |  |  | (0.0008) |  |
| Avemile |  |  | -0.0413^***^ |  |  | -0.0445^***^ |  |  | -0.0233^***^ |  |  | -0.0079^***^ |
|  |  |  | (0.0004) |  |  | (0.0005) |  |  | (0.0007) |  |  | (0.0012) |
| cons | -0.4266^***^ | -0.3209^***^ | -0.3448^***^ | -0.4179^***^ | -0.3029^***^ | -0.3397^***^ | -0.4923^***^ | -0.4637^***^ | -0.3537^***^ | -0.5315^***^ | -0.5163^***^ | -0.5083^***^ |
|  | (0.0009) | (0.0014) | (0.0012) | (0.0010) | (0.0015) | (0.0013) | (0.0019) | (0.0031) | (0.0021) | (0.0012) | (0.0034) | (0.0035) |
| N | 1369978 | 1369978 | 1369978 | 1172398 | 1172398 | 1172398 | 162560 | 162560 | 162560 | 35020 | 35020 | 35020 |
| R^2^ | 0.0020 | 0.0114 | 0.0126 | 0.0034 | 0.0144 | 0.0136 | 0.0103 | 0.0013 | 0.0069 | 0.0000 | 0.0009 | 0.0019 |

**Supplementary Table 47.** Heterogeneity analysis: Taxi BEVs for lnCO

|  | (1)Shanghai and Shenzhen | | | (2)Shanghai | | | (3)Shenzhen | | |
| --- | --- | --- | --- | --- | --- | --- | --- | --- | --- |
|  | lnCO | lnCO | lnCO | lnCO | lnCO | lnCO | lnCO | lnCO | lnCO |
| BEVtrips | 0.0151^***^ |  |  | 0.0153^***^ |  |  | 0.0151^***^ |  |  |
|  | (0.0005) |  |  | (0.0009) |  |  | (0.0006) |  |  |
| lnmileage |  | 0.0099^***^ |  |  | 0.0129^***^ |  |  | 0.0094^***^ |  |
|  |  | (0.0004) |  |  | (0.0009) |  |  | (0.0004) |  |
| Avemile |  |  | 0.0037^***^ |  |  | -0.0013 |  |  | 0.0045^***^ |
|  |  |  | (0.0005) |  |  | (0.0011) |  |  | (0.0005) |
| cons | -0.5181^***^ | -0.5513^***^ | -0.5112^***^ | -0.4779^***^ | -0.5249^***^ | -0.4122^***^ | -0.5255^***^ | -0.5580^***^ | -0.5286^***^ |
|  | (0.0008) | (0.0021) | (0.0020) | (0.0035) | (0.0077) | (0.0047) | (0.0007) | (0.0021) | (0.0022) |
| N | 141762 | 141762 | 141762 | 21829 | 21829 | 21829 | 119917 | 119917 | 119917 |
| R^2^ | 0.0070 | 0.0057 | 0.0004 | 0.0087 | 0.0070 | 0.0000 | 0.0066 | 0.0055 | 0.0007 |

**Supplementary Table 48.** Heterogeneity analysis: Ride-hailing BEVs for lnCO

|  | (1)Three cities | | | (2)Beijing | | | (3)Shanghai | | | (4)Shenzhen | | |
| --- | --- | --- | --- | --- | --- | --- | --- | --- | --- | --- | --- | --- |
|  | lnCO | lnCO | lnCO | lnCO | lnCO | lnCO | lnCO | lnCO | lnCO | lnCO | lnCO | lnCO |
| BEVtrips | 0.0061^***^ |  |  | 0.0027^***^ |  |  | 0.0133^***^ |  |  | 0.0070^***^ |  |  |
|  | (0.0004) |  |  | (0.0008) |  |  | (0.0006) |  |  | (0.0005) |  |  |
| lnmileage |  | -0.0007^**^ |  |  | -0.0044^***^ |  |  | 0.0069^***^ |  |  | 0.0006 |  |
|  |  | (0.0003) |  |  | (0.0006) |  |  | (0.0005) |  |  | (0.0004) |  |
| Avemile |  |  | -0.0112^***^ |  |  | -0.0202^***^ |  |  | -0.0085^***^ |  |  | -0.0053^***^ |
|  |  |  | (0.0005) |  |  | (0.0012) |  |  | (0.0009) |  |  | (0.0005) |
| cons | -0.4929^***^ | -0.4739^***^ | -0.4355^***^ | -0.4647^***^ | -0.4217^***^ | -0.3781^***^ | -0.4742^***^ | -0.4781^***^ | -0.3916^***^ | -0.5238^***^ | -0.5207^***^ | -0.4980^***^ |
|  | (0.0010) | (0.0021) | (0.0019) | (0.0028) | (0.0048) | (0.0045) | (0.0023) | (0.0042) | (0.0034) | (0.0004) | (0.0017) | (0.0018) |
| N | 314355 | 314355 | 314355 | 116283 | 116283 | 116283 | 55353 | 55353 | 55353 | 142719 | 142719 | 142719 |
| R^2^ | 0.0010 | 0.0000 | 0.0022 | 0.0002 | 0.0006 | 0.0040 | 0.0071 | 0.0026 | 0.0014 | 0.0017 | 0.0000 | 0.0011 |

**Supplementary Table 49.** Heterogeneity analysis: Private BEVs for lnSO2

|  | (1)Three cities | | | (2)Beijing | | | (3)Shanghai | | | (4)Shenzhen | | |
| --- | --- | --- | --- | --- | --- | --- | --- | --- | --- | --- | --- | --- |
|  | lnSO_2_ | lnSO_2_ | lnSO_2_ | lnSO_2_ | lnSO_2_ | lnSO_2_ | lnSO_2_ | lnSO_2_ | lnSO_2_ | lnSO_2_ | lnSO_2_ | lnSO_2_ |
| BEVtrips | -0.0300^***^ |  |  | -0.0356^***^ |  |  | 0.0156^***^ |  |  | 0.0001 |  |  |
|  | (0.0004) |  |  | (0.0004) |  |  | (0.0009) |  |  | (0.0016) |  |  |
| lnmileage |  | -0.0437^***^ |  |  | -0.0475^***^ |  |  | -0.0155^***^ |  |  | -0.0013 |  |
|  |  | (0.0003) |  |  | (0.0003) |  |  | (0.0007) |  |  | (0.0011) |  |
| Avemile |  |  | -0.0584^***^ |  |  | -0.0594^***^ |  |  | -0.0635^***^ |  |  | -0.0029^**^ |
|  |  |  | (0.0005) |  |  | (0.0006) |  |  | (0.0013) |  |  | (0.0015) |
| cons | 1.4370^***^ | 1.6043^***^ | 1.5109^***^ | 1.3783^***^ | 1.5508^***^ | 1.4359^***^ | 1.7693^***^ | 1.9186^***^ | 2.0081^***^ | 1.7092^***^ | 1.7146^***^ | 1.7181^***^ |
|  | (0.0012) | (0.0019) | (0.0015) | (0.0013) | (0.0021) | (0.0016) | (0.0031) | (0.0042) | (0.0039) | (0.0017) | (0.0045) | (0.0044) |
| N | 1369978 | 1369978 | 1369978 | 1172398 | 1172398 | 1172398 | 162560 | 162560 | 162560 | 35020 | 35020 | 35020 |
| R^2^ | 0.0090 | 0.0259 | 0.0170 | 0.0121 | 0.0292 | 0.0164 | 0.0039 | 0.0048 | 0.0351 | 0.0000 | 0.0001 | 0.0002 |

**Supplementary Table 50.** Heterogeneity analysis: Taxi BEVs for lnSO2

|  | (1)Shanghai and Shenzhen | | | (2)Shanghai | | | (3)Shenzhen | | |
| --- | --- | --- | --- | --- | --- | --- | --- | --- | --- |
|  | lnSO_2_ | lnSO_2_ | lnSO_2_ | lnSO_2_ | lnSO_2_ | lnSO_2_ | lnSO_2_ | lnSO_2_ | lnSO_2_ |
| BEVtrips | -0.0124^***^ |  |  | -0.0184^***^ |  |  | -0.0105^***^ |  |  |
|  | (0.0007) |  |  | (0.0016) |  |  | (0.0008) |  |  |
| lnmileage |  | -0.0016^***^ |  |  | -0.0039^***^ |  |  | -0.0012^**^ |  |
|  |  | (0.0005) |  |  | (0.0014) |  |  | (0.0005) |  |
| Avemile |  |  | 0.0094^***^ |  |  | 0.0246^***^ |  |  | 0.0069^***^ |
|  |  |  | (0.0007) |  |  | (0.0019) |  |  | (0.0007) |
| cons | 1.7298^***^ | 1.7205^***^ | 1.6731^***^ | 1.9057^***^ | 1.8661^***^ | 1.7267^***^ | 1.7001^***^ | 1.6956^***^ | 1.6612^***^ |
|  | (0.0011) | (0.0028) | (0.0028) | (0.0063) | (0.0113) | (0.0083) | (0.0009) | (0.0027) | (0.0029) |
| N | 141762 | 141762 | 141762 | 21829 | 21829 | 21829 | 119917 | 119917 | 119917 |
| R^2^ | 0.0028 | 0.0001 | 0.0016 | 0.0083 | 0.0004 | 0.0085 | 0.0019 | 0.0001 | 0.0009 |

**Supplementary Table 51.** Heterogeneity analysis: Ride-hailing BEVs for lnSO2

|  | (1)Three cities | | | (2)Beijing | | | (3)Shanghai | | | (4)Shenzhen | | |
| --- | --- | --- | --- | --- | --- | --- | --- | --- | --- | --- | --- | --- |
|  | lnSO_2_ | lnSO_2_ | lnSO_2_ | lnSO_2_ | lnSO_2_ | lnSO_2_ | lnSO_2_ | lnSO_2_ | lnSO_2_ | lnSO_2_ | lnSO_2_ | lnSO_2_ |
| BEVtrips | 0.0013^**^ |  |  | -0.0003 |  |  | 0.0013 |  |  | 0.0042^***^ |  |  |
|  | (0.0006) |  |  | (0.0011) |  |  | (0.0009) |  |  | (0.0008) |  |  |
| lnmileage |  | -0.0074^***^ |  |  | -0.0180^***^ |  |  | 0.0015^*^ |  |  | 0.0024^***^ |  |
|  |  | (0.0005) |  |  | (0.0009) |  |  | (0.0008) |  |  | (0.0005) |  |
| Avemile |  |  | -0.0205^***^ |  |  | -0.0579^***^ |  |  | 0.0014 |  |  | 0.0005 |
|  |  |  | (0.0007) |  |  | (0.0017) |  |  | (0.0013) |  |  | (0.0006) |
| cons | 1.5781^***^ | 1.6274^***^ | 1.6598^***^ | 1.3043^***^ | 1.4374^***^ | 1.5230^***^ | 1.8271^***^ | 1.8206^***^ | 1.8267^***^ | 1.7069^***^ | 1.6995^***^ | 1.7086^***^ |
|  | (0.0015) | (0.0030) | (0.0027) | (0.0042) | (0.0068) | (0.0063) | (0.0034) | (0.0060) | (0.0053) | (0.0006) | (0.0023) | (0.0024) |
| N | 314355 | 314355 | 314355 | 116283 | 116283 | 116283 | 55353 | 55353 | 55353 | 142719 | 142719 | 142719 |
| R^2^ | 0.0000 | 0.0014 | 0.0042 | 0.0000 | 0.0059 | 0.0188 | 0.0000 | 0.0001 | 0.0000 | 0.0003 | 0.0002 | 0.0000 |

**Supplementary Table 52.** Heterogeneity analysis: Private BEVs for lnNO2

|  | (1)Three cities | | | (2)Beijing | | | (3)Shanghai | | | (4)Shenzhen | | |
| --- | --- | --- | --- | --- | --- | --- | --- | --- | --- | --- | --- | --- |
|  | lnNO_2_ | lnNO_2_ | lnNO_2_ | lnNO_2_ | lnNO_2_ | lnNO_2_ | lnNO_2_ | lnNO_2_ | lnNO_2_ | lnNO_2_ | lnNO_2_ | lnNO_2_ |
| BEVtrips | 0.0236^***^ |  |  | 0.0205^***^ |  |  | 0.0550^***^ |  |  | 0.0016 |  |  |
|  | (0.0004) |  |  | (0.0005) |  |  | (0.0011) |  |  | (0.0026) |  |  |
| lnmileage |  | -0.0093^***^ |  |  | -0.0120^***^ |  |  | 0.0171^***^ |  |  | -0.0074^***^ |  |
|  |  | (0.0003) |  |  | (0.0004) |  |  | (0.0008) |  |  | (0.0019) |  |
| Avemile |  |  | -0.0726^***^ |  |  | -0.0757^***^ |  |  | -0.0611^***^ |  |  | -0.0180^***^ |
|  |  |  | (0.0006) |  |  | (0.0007) |  |  | (0.0015) |  |  | (0.0026) |
| cons | 3.3245^***^ | 3.4528^***^ | 3.6053^***^ | 3.3073^***^ | 3.4430^***^ | 3.5868^***^ | 3.4432^***^ | 3.5184^***^ | 3.8058^***^ | 3.2098^***^ | 3.2418^***^ | 3.2656^***^ |
|  | (0.0014) | (0.0019) | (0.0017) | (0.0015) | (0.0021) | (0.0019) | (0.0037) | (0.0053) | (0.0044) | (0.0028) | (0.0077) | (0.0079) |
| N | 1369978 | 1369978 | 1369978 | 1172398 | 1172398 | 1172398 | 162560 | 162560 | 162560 | 35020 | 35020 | 35020 |
| R^2^ | 0.0051 | 0.0011 | 0.0242 | 0.0039 | 0.0018 | 0.0257 | 0.0291 | 0.0035 | 0.0198 | 0.0000 | 0.0007 | 0.0019 |

**Supplementary Table 53.** Heterogeneity analysis: Taxi BEVs for lnNO2

|  | (1)Shanghai and Shenzhen | | | (2)Shanghai | | | (3)Shenzhen | | |
| --- | --- | --- | --- | --- | --- | --- | --- | --- | --- |
|  | lnNO_2_ | lnNO_2_ | lnNO_2_ | lnNO_2_ | lnNO_2_ | lnNO_2_ | lnNO_2_ | lnNO_2_ | lnNO_2_ |
| BEVtrips | 0.0180^***^ |  |  | -0.0029 |  |  | 0.0247^***^ |  |  |
|  | (0.0012) |  |  | (0.0020) |  |  | (0.0014) |  |  |
| lnmileage |  | 0.0212^***^ |  |  | 0.0283^***^ |  |  | 0.0200^***^ |  |
|  |  | (0.0008) |  |  | (0.0018) |  |  | (0.0009) |  |
| Avemile |  |  | 0.0223^***^ |  |  | 0.0614^***^ |  |  | 0.0161^***^ |
|  |  |  | (0.0011) |  |  | (0.0025) |  |  | (0.0012) |
| cons | 3.2881^***^ | 3.1963^***^ | 3.2228^***^ | 3.6532^***^ | 3.4078^***^ | 3.3745^***^ | 3.2298^***^ | 3.1533^***^ | 3.1895^***^ |
|  | (0.0017) | (0.0044) | (0.0045) | (0.0078) | (0.0147) | (0.0107) | (0.0015) | (0.0044) | (0.0050) |
| N | 141762 | 141762 | 141762 | 21829 | 21829 | 21829 | 119917 | 119917 | 119917 |
| R^2^ | 0.0027 | 0.0070 | 0.0041 | 0.0001 | 0.0134 | 0.0317 | 0.0044 | 0.0062 | 0.0021 |

**Supplementary Table 54.** Heterogeneity analysis: Ride-hailing BEVs for lnNO2

|  | (1)Three cities | | | (2)Beijing | | | (3)Shanghai | | | (4)Shenzhen | | |
| --- | --- | --- | --- | --- | --- | --- | --- | --- | --- | --- | --- | --- |
|  | lnNO_2_ | lnNO_2_ | lnNO_2_ | lnNO_2_ | lnNO_2_ | lnNO_2_ | lnNO_2_ | lnNO_2_ | lnNO_2_ | lnNO_2_ | lnNO_2_ | lnNO_2_ |
| BEVtrips | 0.0348^***^ |  |  | 0.0449^***^ |  |  | 0.0184^***^ |  |  | 0.0287^***^ |  |  |
|  | (0.0007) |  |  | (0.0011) |  |  | (0.0011) |  |  | (0.0013) |  |  |
| lnmileage |  | 0.0145^***^ |  |  | 0.0203^***^ |  |  | 0.0204^***^ |  |  | 0.0038^***^ |  |
|  |  | (0.0005) |  |  | (0.0008) |  |  | (0.0010) |  |  | (0.0009) |  |
| Avemile |  |  | -0.0176^***^ |  |  | -0.0311^***^ |  |  | 0.0182^***^ |  |  | -0.0189^***^ |
|  |  |  | (0.0009) |  |  | (0.0019) |  |  | (0.0015) |  |  | (0.0011) |
| cons | 3.2941^***^ | 3.2870^***^ | 3.4444^***^ | 3.2341^***^ | 3.2477^***^ | 3.5171^***^ | 3.5634^***^ | 3.4753^***^ | 3.5593^***^ | 3.2369^***^ | 3.2428^***^ | 3.3325^***^ |
|  | (0.0017) | (0.0032) | (0.0035) | (0.0039) | (0.0060) | (0.0073) | (0.0042) | (0.0074) | (0.0059) | (0.0011) | (0.0040) | (0.0042) |
| N | 314355 | 314355 | 314355 | 116283 | 116283 | 116283 | 55353 | 55353 | 55353 | 142719 | 142719 | 142719 |
| R^2^ | 0.0132 | 0.0037 | 0.0022 | 0.0282 | 0.0086 | 0.0063 | 0.0051 | 0.0084 | 0.0024 | 0.0056 | 0.0002 | 0.0027 |

**Supplementary Table 55.** Heterogeneity analysis: Private BEVs for lnO3

|  | (1)Three cities | | | (2)Beijing | | | (3)Shanghai | | | (4)Shenzhen | | |
| --- | --- | --- | --- | --- | --- | --- | --- | --- | --- | --- | --- | --- |
|  | lnO_3_ | lnO_3_ | lnO_3_ | lnO_3_ | lnO_3_ | lnO_3_ | lnO_3_ | lnO_3_ | lnO_3_ | lnO_3_ | lnO_3_ | lnO_3_ |
| BEVtrips | -0.0074^***^ |  |  | -0.0058^***^ |  |  | -0.0237^***^ |  |  | 0.0036 |  |  |
|  | (0.0007) |  |  | (0.0008) |  |  | (0.0012) |  |  | (0.0031) |  |  |
| lnmileage |  | 0.0387^***^ |  |  | 0.0428^***^ |  |  | 0.0085^***^ |  |  | -0.0069^***^ |  |
|  |  | (0.0005) |  |  | (0.0006) |  |  | (0.0009) |  |  | (0.0022) |  |
| Avemile |  |  | 0.1201^***^ |  |  | 0.1316^***^ |  |  | 0.0622^***^ |  |  | -0.0193^***^ |
|  |  |  | (0.0010) |  |  | (0.0011) |  |  | (0.0014) |  |  | (0.0033) |
| cons | 4.1411^***^ | 3.8876^***^ | 3.7748^***^ | 4.1243^***^ | 3.8504^***^ | 3.7320^***^ | 4.3119^***^ | 4.1799^***^ | 4.0499^***^ | 3.9832^***^ | 4.0152^***^ | 4.0452^***^ |
|  | (0.0023) | (0.0031) | (0.0029) | (0.0025) | (0.0035) | (0.0032) | (0.0039) | (0.0055) | (0.0041) | (0.0033) | (0.0091) | (0.0099) |
| N | 1369978 | 1369978 | 1369978 | 1172398 | 1172398 | 1172398 | 162560 | 162560 | 162560 | 35020 | 35020 | 35020 |
| R^2^ | 0.0002 | 0.0061 | 0.0217 | 0.0001 | 0.0070 | 0.0235 | 0.0051 | 0.0008 | 0.0193 | 0.0001 | 0.0004 | 0.0015 |

**Supplementary Table 56.** Heterogeneity analysis: Taxi BEVs for lnO3

|  | (1)Shanghai and Shenzhen | | | (2)Shanghai | | | (3)Shenzhen | | |
| --- | --- | --- | --- | --- | --- | --- | --- | --- | --- |
|  | lnO_3_ | lnO_3_ | lnO_3_ | lnO_3_ | lnO_3_ | lnO_3_ | lnO_3_ | lnO_3_ | lnO_3_ |
| BEVtrips | 0.0118^***^ |  |  | -0.0074^***^ |  |  | 0.0181^***^ |  |  |
|  | (0.0012) |  |  | (0.0015) |  |  | (0.0014) |  |  |
| lnmileage |  | 0.0072^***^ |  |  | -0.0102^***^ |  |  | 0.0102^***^ |  |
|  |  | (0.0009) |  |  | (0.0016) |  |  | (0.0010) |  |
| Avemile |  |  | 0.0019^*^ |  |  | -0.0071^***^ |  |  | 0.0034^***^ |
|  |  |  | (0.0011) |  |  | (0.0017) |  |  | (0.0013) |
| cons | 4.0482^***^ | 4.0252^***^ | 4.0576^***^ | 4.2540^***^ | 4.3091^***^ | 4.2560^***^ | 4.0181^***^ | 3.9847^***^ | 4.0230^***^ |
|  | (0.0017) | (0.0048) | (0.0047) | (0.0060) | (0.0132) | (0.0072) | (0.0015) | (0.0049) | (0.0053) |
| N | 141762 | 141762 | 141762 | 21829 | 21829 | 21829 | 119917 | 119917 | 119917 |
| R^2^ | 0.0009 | 0.0006 | 0.0000 | 0.0008 | 0.0016 | 0.0004 | 0.0017 | 0.0012 | 0.0001 |

**Supplementary Table 57.** Heterogeneity analysis: Ride-hailing BEVs for lnO3

|  | (1)Three cities | | | (2)Beijing | | | (3)Shanghai | | | (4)Shenzhen | | |
| --- | --- | --- | --- | --- | --- | --- | --- | --- | --- | --- | --- | --- |
|  | lnO_3_ | lnO_3_ | lnO_3_ | lnO_3_ | lnO_3_ | lnO_3_ | lnO_3_ | lnO_3_ | lnO_3_ | lnO_3_ | lnO_3_ | lnO_3_ |
| BEVtrips | -0.0150^***^ |  |  | -0.0283^***^ |  |  | -0.0141^***^ |  |  | 0.0082^***^ |  |  |
|  | (0.0011) |  |  | (0.0019) |  |  | (0.0011) |  |  | (0.0015) |  |  |
| lnmileage |  | -0.0039^***^ |  |  | -0.0001 |  |  | -0.0119^***^ |  |  | -0.0049^***^ |  |
|  |  | (0.0008) |  |  | (0.0014) |  |  | (0.0010) |  |  | (0.0010) |  |
| Avemile |  |  | 0.0135^***^ |  |  | 0.0606^***^ |  |  | -0.0036^**^ |  |  | -0.0163^***^ |
|  |  |  | (0.0012) |  |  | (0.0029) |  |  | (0.0016) |  |  | (0.0013) |
| cons | 4.1289^***^ | 4.1171^***^ | 4.0414^***^ | 4.2021^***^ | 4.0995^***^ | 3.8684^***^ | 4.2922^***^ | 4.3310^***^ | 4.2543^***^ | 4.0250^***^ | 4.0546^***^ | 4.0938^***^ |
|  | (0.0025) | (0.0047) | (0.0047) | (0.0068) | (0.0103) | (0.0108) | (0.0042) | (0.0076) | (0.0063) | (0.0012) | (0.0046) | (0.0049) |
| N | 314355 | 314355 | 314355 | 116283 | 116283 | 116283 | 55353 | 55353 | 55353 | 142719 | 142719 | 142719 |
| R^2^ | 0.0012 | 0.0001 | 0.0006 | 0.0033 | 0.0000 | 0.0070 | 0.0030 | 0.0028 | 0.0001 | 0.0003 | 0.0002 | 0.0015 |

**References**

Liang, X. et al. Air quality and health benefits from fleet electrification in China. *Nat. Sustain.* **2**, 962–971 (2019).

Milovanoff, A., Posen, I. D. & MacLean, H. L. Electrification of light-duty vehicle fleet alone will not meet mitigation targets. *Nat. Clim. Chang.* **10**, 1102–1107 (2020).

Lu, Q., Duan, H., Shi, H. et al. Decarbonization scenarios and carbon reduction potential for China’s road transportation by 2060. *npj Urban Sustain.* **2**, 34 (2022).

1. Abdul-Manan, A F.N. et al. Electrifying passenger road transport in India requires near-term electricity grid decarbonization. *Nat Commun.***13**, 1-13(2022).

Milovanoff, A. et al. A dynamic fleet model of US light-duty vehicle lightweighting and associated greenhouse gas emissions from 2016 to 2050. *Environ. Sci. Technol.* **53**, 2199-2208(2019).

Jenn A. Emissions benefits of electric vehicles in Uber and Lyft ride-hailing services. *Nature energy*.**5**, 520–525 (2020).

Böhm, M., Nanni, M. & Pappalardo, L. Gross polluters and vehicle emissions reduction. *Nat Sustain* **5**, 699–707 (2022).

8. Zhang,X., Bai,X. Incentive policies from 2006 to 2016 and new energy vehicle adoption in 2010-2020 in China. *Renew Sustain Energy Rev*.**70**,24-43(2017).

9. Milovanoff, A., Posen, I.D. & MacLean, H.L. Electrification of light-duty vehicle fleet alone will not meet mitigation targets. *Nat. Clim. Chang*. **10**, 1102–1107(2020).

10. Wang K.L., Zheng.L.J., Zhang.J.Z. & Yao.H.J. The impact of promoting new energy vehicles on carbon intensity Causal evidence from China. *Energy Econ*,**114**,106255(2022).

11. Wang, Y.,Wen, Y., Xu, Y., Shi, L. & Yang, X. Health Benefits Quantification for New-Energy Vehicles Promotion: A Case Study of Beijing. *Int. J. Environ. Res. Public Health***19**, 13876(2022).

12. Li, Y., Ha, N. & Li, T. Research on carbon emissions of electric vehicles throughout the life cycle assessment taking into vehicle weight and grid mix composition. *Energies* **12**, 3612(2019).

13. Zhang,X.D., Zou,Y., Fan,J., Guo,H.W. Usage pattern analysis of Beijing private electric vehicles based on real-world data,*Energy* **167**,1074-1085(2019).

14. Hua, C., Jia, X., Chiu, A S.F., Hu, X. & Ming,X. Siting public electric vehicle charging stations in Beijing using big-data informed travel patterns of the taxi fleet. *Transp Res D Transp Environ Dec*.**33**,39-46(2014).

15.Barnes,S.J., Guo,Y. & Borgo,R.Sharing the air: Transient impacts of ride-hailing introduction on pollution in China.*Transp Res D Tran sp Environ.* **86**,102434(2020).

16. Wu，Y. et al. Energy consumption and CO_2_ emission impacts of vehicle electrification in three developed regions of China. *Energy Policy* **48**, 537–550(2012).

17. Abdul-Manan, A F.N. et al. Electrifying passenger road transport in India requires near-term electricity grid decarbonization. *Nat Commun.***13**, 1-13(2022).

18.Ou, S.Q., Yu, R.J., Lin, Z.H., Ren, H.H., He, X., Przesmitzki, S., Bouchard, J. Intensity and daily pattern of passenger vehicle use by region and class in China: estimation and implications for energy use and electrification. *Mitig Adapt Strateg Glob Chang*. **25**,307-327(2020).

19.*National survey on the actual operation of electric vehicles: the average daily driving of private passenger cars is about 65 kilometers*(Autohome,2020); https://chejiahao.autohome.com.cn/info/6344319

20. Lang, J., Cheng, S., Zhou, Y., Zhang, Y. & Wang, G. Air pollutant emissions from on-road vehicles in China, 1999–2011. *Sci. Total Environ* **496**,1-10 (2014).

21. Anenberg, S. et al. Impacts and mitigation of excess diesel-related NOx emissions in 11 major vehicle markets. *Nature* **545**, 467–471 (2017).

22. *COPERT III Computer programme to calculate emissions from road transport* (European Environment Agency，2000); https://www.eea.europa.eu/publications/Technical_report_No_49

23. *Gasoline for motor vehicles* (National standard full text disclosure system, 2016)

https://openstd.samr.gov.cn/bzgk/gb/newGbInfo?hcno=C45A3554980A86E41F5AA4C6F3D48DC1.

24. Calculation method of standard coal conversion coefficient of National Bureau of Statistics (Docin，2015);https://www.docin.com/p-1307077496.html

25. China Statistical Yearbook 2019 (Compiled by National Burcau of Statistics of China,2019); http://www.stats.gov.cn/tjsj/ndsj/2019/indexch.html

26. Standard coal (Baidu Encyclopedia, 2021);https://baike.baidu.com/item/%E6%A0%87%E5%87%86%E7%85%A4/11020648

27. Chen, X.Y.,Zhang, H.C., Xu, Z.W., Nielsen, C.P.,McElroy, M.B.,Lv, J.J. Impacts of fleet types and charging modes for electric vehicles on emissions under different penetrations of wind power. *Nat. Energy*.**3**, 413–421 (2018).

28.*China Energy Statistical Yearbook 2020* (Statistical database of Economic and Social development of Tibet Autonomous Region,2020); http://www.stats.gov.cn/sj/ndsj/2020/indexeh.htm

29. Ou, S., Yu, R., Lin, Z. et al. Intensity and daily pattern of passenger vehicle use by region and class in China: estimation and implications for energy use and electrification. *Mitig Adapt Strateg Glob Change* **25**, 307–327 (2020).

30. *Notice of the State Council on Printing and Distributing ‘the Action Plan for Carbon Peak by 2030’* (The Central People's Government of the People's Republic of China,2021); http://www.gov.cn/zhengce/content/2021-10/26/content_5644984.htm

31. Ferrero, E., Alessandrini, S. & Balanzino, A. Impact of the electric vehicles on the air pollution from a highway. *Appl. Energy* **169**, 450–459 (2016).

32. Wu, X. K., Freese, D., Cabrera, A. & Kitch, W. A. Electric vehicles’ energy consumption measurement and estimation. *Transp. Res. D Transp. Environ.* **34**, 52–67, 1361–9209 (2015).

33. *‘PM2.5 in the Air’ 20 questions* (Chinese Academy of Sciences，2012); https://www.cas.cn/kxcb/kpwz/201303/t20130318_3795074.shtml
